# Use of Measured Residual Dipolar Couplings to Calculate Residual Dipolar Couplings for a Protein Structure: A Case Study Using Hen Egg-White Lysozyme

**DOI:** 10.1021/acs.jcim.5c01428

**Published:** 2025-09-26

**Authors:** Maria Pechlaner, Wilfred F. van Gunsteren, Niels Hansen, Lorna J. Smith

**Affiliations:** † Institute of Molecular Physical Sciences, ETH Zurich, CH-8093 Zurich, Switzerland; ‡ Institute of Thermodynamics and Thermal Process Engineering, 9149 University of Stuttgart, D-70569 Stuttgart, Germany; § Department of Chemistry, Inorganic Chemistry Laboratory, 6396 University of Oxford, South Parks Road, Oxford OX1 3QR, U.K.

## Abstract

Five sets of RDC values for the backbone of [^13^C,^15^N]-labeled Hen Egg-White Lysozyme (HEWL, 320 RDCs), obtained from NMR experiments of the protein in an ether bicelle solution at a temperature of 308 K and pH 3.8, were used to calculate RDC values by application of two methods, the alignment-tensor (AT) method and the method of magnetic-field rotational sampling (HRS), applied to five X-ray structures of HEWL, to investigate the relevance of measured RDC values for the structure determination or refinement of proteins. In contrast to other quantities *Q* observable by NMR, such as NOE intensities or ^3^ *J*-couplings, for which a relation *Q*(*r*) between the quantity *Q* and a single structure *r* of a protein can be used to calculate average values ⟨*Q*(*r*)⟩, averaged over the Boltzmann-weighted structural ensemble of the protein at finite temperature in solution, an RDC is not defined in terms of a single structure but as an average over a slightly nonuniform rotational and orientation distribution of the protein. This averaging between large positive and negative values reduces the kHz size of a dipolar coupling (DC) by a factor of 10^3^ to 10^4^ to the Hz range of a residual dipolar coupling (RDC). Since the nonuniform orientation distribution can neither be measured nor faithfully mimicked at atomic resolution on a computer, RDC values for a given protein structure are commonly calculated by minimizing the difference between calculated and measured RDC values for a given set of measured target RDC values by varying the orientation distribution of the protein in one way or the other. These three features of RDCs, a very large reduction of size as a result of averaging over orientations, their definition in terms of an unknown, immeasurable orientation distribution, and their calculation using a set of target RDC values, lead to a sensitivity of the calculated RDC values to the size and type of the particular set of RDCs used in the calculation. This reduces the usefulness of measured RDCs for structure determination or refinement of proteins compared to NOE intensities or ^3^ *J*-couplings.

## Graphical abstract

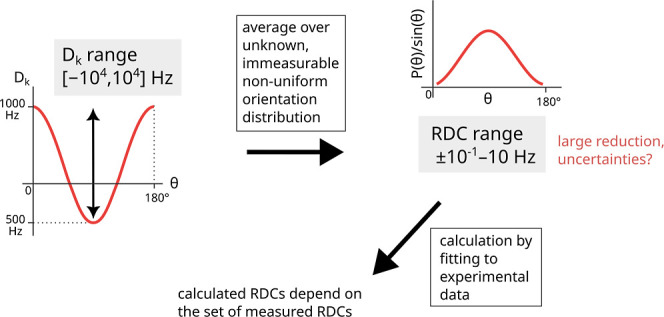

## 1 Introduction

Over the past 30 years, structure determination of proteins based on Nuclear Magnetic Resonance (NMR) spectroscopic measurements of a variety of observable quantities has become a standard technique in biochemistry and molecular biology.^1–4^ Some observable quantities, such as nuclear Overhauser enhancement intensities (NOEs), ^3^ *J*-couplings or chemical shifts, only provide local structural information. By contrast, residual dipolar couplings (RDCs) provide longer-range information in terms of the average (relative) direction of bond vectors throughout a protein.^5–7^

A dipolar coupling *D*_*k*_1_ *k*_2__ or *D*_*k*_, for short, between two nuclear spins *k*_1_ and *k*_2_ in a homogeneous magnetic field *H⃗* depends on^5–7^ (i) the length $r_{k_{1}k_{2}}\equiv \left|{\mathrm{r⃗}}_{k_{1}k_{2}}\right|$ of the internuclear vector ${\mathrm{r⃗}}_{k_{1}k_{2}}\equiv {\mathrm{r⃗}}_{k_{1}}-{\mathrm{r⃗}}_{k_{2}}$ and (ii) the angle θ_*k*_ of this vector with the static magnetic field *H⃗*, e.g. $\cos\left(\theta_{k}\right)=\left({\mathrm{r⃗}}_{h_{1}h_{2}}\cdot {\mathrm{r⃗}}_{k_{1}k_{2}}\right)/\left({\mathrm{r⃗}}_{h_{1}h_{2}}\cdot {\mathrm{r⃗}}_{k_{1}k_{2}}\right)$ , where *H⃗* is represented by a two-atomic molecule or two-particle vector rigidly connecting atoms or particles *h*_1_ and *h*_2_, *r⃗*_*h*_1_ *h*_2__ ≡ *r⃗*_*h*_1__ – *r⃗*_*h*_2__, (*r⃗*_*h*_1_ *h*_2__ *r⃗*_*k*_1_ *k*_2__) is the scalar product of the two vectors *r⃗*_*h*_1_ *h*_2__ and *r⃗*_*k*_1_ *k*_2__, and

$$D_{k}=\frac{-\mu_{0}h\gamma_{k_{1}}\gamma_{k_{2}}}{8\pi^{3}}\left(\frac{3\;\cos^{2}\left(\theta_{k}\right)-1}{2r_{k}^{3}}\right)$$ 1

Here, the shorthand notation *r*_*k*_ ≡ *r*_*k*_1_ *k*_2__ is used, μ_0_ is the magnetic susceptibility of vacuum (ε_0_ *c* ^2^)^−1^ = 4π 10^–7^ J s^2^/(C^2^ m) or 1.9425913 × 10^–8^ kJ mol^–1^ ps^2^/(e^2^ nm), *h* is Planck’s constant, 6.626176 × 10^–34^ J s or 0.3990313 kJ mol^–1^ ps, and γ_*i*_ is the gyromagnetic ratio^8,9^ of nucleus *i*.

The constant and variable factors in eq 1 can be separated as follows

2 $$D_{k}\left(t\right)=D_{k}^{c}{\left(\frac{r^{0}}{r_{{k_{1}k}_{2}}\left(t\right)}\right)}^{3}\left(\frac{1}{2}\left(3\;\cos^{2}\left(\theta_{k}\left(t\right)\right)-1\right)\right)=D_{k}^{c}R_{k}\left(t\right)P_{k}\left(t\right)$$

with

$$D_{k}^{c}\equiv \frac{-\mu_{0}h}{2\pi {\left(r^{0}\right)}^{3}}\left(\frac{\gamma_{k_{1}}}{2\pi }\right)\left(\frac{\gamma_{k_{2}}}{2\pi }\right)$$ 3

4 $$R_{k}\left(t\right)\equiv {\left(\frac{r^{0}}{r_{{k_{1}k}_{2}}\left(t\right)}\right)}^{3}$$

5 $$P_{k}\left(t\right)\equiv \frac{1}{2}\left(3\;\cos^{2}\left(\theta_{k}\left(t\right)\right)-1\right)$$

The second-order Legendre function *P*_*k*_(θ_*k*_(*t*)) has two zeros, at the so-called magic angles (|cos­(θ)| = 3^–1/2^) 54° and 125°, two maxima of 1 at 0° and 180°, and a minimum of −1/2 at 90°. It is symmetric around θ = 90°. Since the distance between nuclei *k*_1_ and *k*_2_ is generally of the order of 0.1 nm, one may choose *r* ^0^ = 0.1 nm, which yields −μ_0_ *h*/(2π­(*r* ^0^)^3^) = −1.3252 × 10^–10^ J^2^ s^3^ C^–2^ m^–4^ or −1.2336 × 10^–6^ (kJ mol^–1^)^2^ ps^3^ e^–2^ nm^–4^. Approximate values of the gyromagnetic ratio for some nuclei can be found in refs 8,9 . The ones relevant for the current study are given in Table 1 of ref 10. For an N–H pair of nuclei, we find, for example, *D*_*k*_^*c*^ (^14^N–^1^H) = −17.37 kHz and *D*_*k*_^*c*^ (^15^N–^1^H) = +24.36 kHz. We note, however, that a DC is also influenced by external factors, such as neighboring nuclei.^11^ eq 1 is thus an approximation.

In a measurement of RDCs, the value of *D*_*k*_ in eq 1 is averaged over molecules and an experimentally determined time period. A DC *D*_*k*_ generally involves atoms in a molecule that are covalently bound to each other and so move at a relatively high frequency compared to that of the rotational motion of the molecule because the sampling of the molecular rotational degrees of freedom occurs on a much longer time scale. Thus, one may use the assumption that the fluctuation of the distance *r*_*k*_1_ *k*_2__ is not coupled to the rotational motion of vector *r⃗*_*k*_1_ *k*_2__ with respect to magnetic field direction *H⃗*. So, the averaging of *D*_*k*_ may be separated into an average over (*r*_*k*_1_ *k*_2__)^−3^ and one over the second-order Legendre function of θ_*k*_

6 $$\left\langle D_{k}\right\rangle =D_{k}^{c}\left\langle {\left(\frac{r^{0}}{r_{{k_{1}k}_{2}}}\right)}^{3}\right\rangle \left\langle \left(\frac{1}{2}\left(3\;\cos^{2}\left(\theta_{k}\right)-1\right)\right)\right\rangle$$

The average ⟨(*r* ^0^/*r*_*k*_1_ *k*_2__)^3^⟩ will be close to one where *r*_*k*_1_ *k*_2__ is a fluctuating bond length and *r* ^0^ is a value close to its average. For an iso-tropically tumbling molecule, the average over the function *P*_*k*_(θ_*k*_) will be zero

7 $$\left\langle P_{k}\left(\theta_{k}\right)\right\rangle \equiv \left\langle \frac{1}{2}\left(3\;\cos^{2}\left(\theta_{k}\right)-1\right)\right\rangle =0$$

leading to ⟨*D*_*k*_⟩ = 0.

However, by introducing an anisotropy in the rotational tumbling of the molecule, one would have ⟨*D*_*k*_⟩ ≠ 0, and the values of ⟨*D*_*k*_⟩ would yield values of the average ⟨*P*_*k*_(θ_*k*_)⟩ and some information about values of the angles θ_*k*_.

Experimentally, such an anisotropy can be induced in different ways,^7^ (i) using the paramagnetic susceptibility of a molecule, (ii) using electrostatic interactions with a molecule, or (iii) by immersing the molecule in a medium that contains some order that will influence the angular distribution *P*(θ) of the angle θ of some axis in the molecule with the direction of the magnetic field. In this way, small values of ⟨*D*_*k*_⟩, called RDC*s*, of the order of Hz, that is 10^3^–10^4^ smaller than the values of the dipolar couplings *D*_*k*_ themselves, which are of the order of kHz, can be obtained. Unfortunately, the size and shape of the experimentally induced anisotropy in the rotational distribution *P*(θ) cannot be determined. This anisotropy will be different for different media, leading to rather different ⟨*D*_*k*_⟩ values for different media.^12^

The common way to handle this problem is to exclude the rotational degrees of freedom of the molecule from the sampling in a calculation of RDCs by assuming a particular shape of the orientation distribution of the molecule with respect to the magnetic field. This involves the assumption that the overall rotation of the molecule is decoupled from its internal motions. In addition, it is often assumed that the orientation or alignment distribution of the molecule has the same effect on different ⟨*D*_*k*_⟩ values (RDCs), i.e., that the molecule is rigid.

A recently proposed alternative approach^10,13,14^ to use measured RDC values for structure determination or refinement of proteins is based on extensively sampling the rotational degrees of freedom by molecular dynamics (MD) or stochastic dynamics (SD) simulation in combination with sampling the molecule-internal and solvent degrees of freedom. By using rotational sampling, as is occurring in the experiment leading to observable RDCs, the algorithms stay close to the experiment and avoid the use of an alignment tensor (AT), and thus the assumptions that the orientation distribution has a particular shape, that the overall rotation of the molecule is decoupled from its internal motions, and that the molecule is rigid. It thus allows for molecular flexibility but requires MD or SD simulations of the molecule that are sufficiently long to sample the rotational distribution well, i.e., long enough to reduce the ⟨*D*_*k*_⟩ values, which should for infinite sampling become zero (if there is no orientation bias), to beyond a value that is about 10^–3^ to 10^–4^ of the values of *D*_*k*_^*c*^. Use of the method of ref 10, which is based on sampling of the rotational degrees of freedom of the molecule by simulating the rotation of the whole molecule (molecule rotational sampling: MRS), generating an orientation distribution without assumptions on its shape, requires MD or SD simulations of microseconds or longer.^10^ Use of the method of ref 14, which is based on sampling the rotational motion of a magnetic-field (*H*) vector in the form of two atoms or particles connected by a rigid bond by SD simulation (magnetic-field rotational sampling: HRS), requires less long simulations,^14^ and it also does not assume a particular shape of the orientation distribution. In both cases, inclusion of a penalty function that drives the calculated RDCs toward the measured ones in the potential energy function of such a simulation then generates an orientation distribution of the molecule (MRS) or of the magnetic-field vector (HRS) and a conformational ensemble of the molecule more or less compatible with the given set of (measured) target RDC values.

The basic idea of the method of rotational sampling of magnetic-field directions by SD simulation (magnetic-field rotational sampling: HRS) is a hybrid simulation scheme in which simulation of the molecular system (*msy*), e.g., MD simulation of a protein in explicit solvent, with RDC restraining alternates with SD simulation of a magnetic-field vector (*mfv*) in vacuo with RDC restraining. From the *mfv* SD simulations, rotationally sampled RDC values for each MD configuration of the molecule in solution in the *msy* simulation are obtained, which are then used to calculate time-averaged RDC values in the *msy* MD simulation of the molecule. The RDC restraining in the *mfv* SD simulations generates a nonuniform rotational distribution of the magnetic-field vector (equivalent to the anisotropic rotational distribution of the solute molecule in the MRS method of ref 10), leading to nonzero RDC values for each molecular configuration. The latter RDC values can then, time-averaged over the molecular configurations, be used to calculate the RDC-restraining forces in the *msy* MD simulation. Thus, the rotational averaging of the molecule is accounted for by time-averaging over magnetic-field orientations in the *mfv* SD simulation, while the configurational averaging of the molecule, important when the molecule is flexible, is accounted for in the *msy* MD simulation of the molecule in explicit solvent.^15^

The *mfv* part of the algorithm comprises an SD simulation of *N*_*mfv*_ time steps of the two-atom magnetic-field vector *r⃗*_*h*_1_ *h*_2__, in which RDC restraining is applied to this vector to minimize the difference between calculated *D*_*k*_ and target *D*_*k*_^0^ values with a force constant *K* ^RDC,*mfv*^ for a given molecular configuration or structure. The *msy* part of the algorithm consists of an SD or MD simulation of the molecule of *N*_*msy*_ time steps, in which RDC-restraining is applied to the molecule to further minimize the difference between calculated *D*_*k*_ and target *D*_*k*_^0^ values with a force constant *K* ^RDC,*msy*^. Choosing *N*_*mfv*_ ≫ 1, *K* ^RDC,*msy*^ = 0 and *N*_*msy*_ = 1 offers the possibility to calculate an anisotropic distribution of the magnetic-field vector that is consistent with a set of RDCs used as target values while at the same time allowing the calculation of RDC values for bond vectors not used as targets.

Application of the commonly used AT method to a beta-heptapeptide solvated in methanol^15^ illustrated the limitations of the assumptions on which the alignment-tensor approach in structure determination or refinement of molecular systems based on measured RDC values rests: (i) an orientation distribution of the molecule in terms of spherical harmonic functions of order 2, (ii) rigidity of the molecule, and (iii) the absence of coupling between rotational and internal motions of the molecule. In addition,^15^ the application of the more general magnetic-field rotational sampling (HRS) algorithm illustrated that experimentally measured RDCs are less useful for molecular structure determination or refinement than other observable quantities measurable by NMR techniques, such as NOE intensities, ^3^ *J*-coupling constants, and *S* ^2^ motional order parameters, because of the particular characteristics of their definition in terms of an unknown orientation distribution of the molecule of interest:

1. The real (anisotropic) orientation distribution of the solute molecule of interest, which is needed to properly calculate the average, that is, the RDC, is not measurable.
2. The real (anisotropic) orientation distribution of the solute molecule of interest, which is needed to properly calculate the average, that is, the RDC, cannot properly be modeled or simulated because of the difficulty of realistically and accurately representing macroscopic conditions at the molecular level of resolution.
3. Therefore, the common way to obtain this orientation distribution is by fitting, for a particular set of RDCs, the RDC values (*D*_*k*_ or ⟨*D*_*k*_⟩) calculated for one or more (modeled or MD-generated trajectory) structures to the (measured) target RDC values (*D*_*k*_^0^).
4. This procedure leads to a dependence of the obtained (anisotropic) orientation distribution and thus of the RDC values calculated from it upon the particular set of target *D*_*k*_^0^ values used in the minimization procedure.

In the current article, both approaches, the alignment-tensor (AT) and the magnetic-field rotational sampling (HRS) method, are applied to five single X-ray structures of the protein hen egg-white lysozyme (HEWL) using RDC values derived from experiment as RDC restraints. Experimentally, 320 RDC values for ^13^C–^15^N labeled HEWL measured at pH = 3.8 and 308 K are available,^16^ 38 and 39 ^13^C^α^–^1^H^α^, 101 ^15^N–^1^H^N^, 97 ^13^C^α^–^13^C′, and 45 ^13^C′–^15^N RDC values.

For the protein HEWL, two issues in particular are addressed: (i) how sensitive are calculated RDC values to the particular (sub)­set (size, type, and distribution over the molecule) of measured RDC values that are used as target values in the minimization or restraining procedure? In other words, how useful are measured RDC values for protein structure determination or refinement? (ii) How may artifacts in the calculation of RDC values due to the minimization or restraining procedure used and due to their definition in terms of an average over an anisotropic rotational distribution of the protein be detected? An RDC value is not defined in terms of a single molecular structure, such as is the case for a ^3^ *J*-coupling, but in terms of a complete (anisotropic) rotational distribution and sampling of the magnetic field direction or vector (in the HRS method) or of the molecule (in the MRS method). This allows quite some freedom to the shape of the rotational distribution that minimizes the difference between calculated ⟨*D*_*k*_⟩ values and target (measured) *D*_*k*_^0^ values but may lead to artifacts such as RDC-(bond)­vector directions biased toward the magic angles.

## 2 RDCs as Restraints in Molecular Simulation or Modeling

### 2.1 Calculation of RDCs Using the Alignment-Tensor Approach

The formulas to calculate RDCs and the RDC-restraining forces when applying the alignment-tensor approach have been given in refs 13,17 . There it is assumed that the bonds of the molecule are kept rigid, as is the case in the present study. In ref 13, the alignment-tensor approach was extended with the possibility to allow time-averaging of five quantities determining the AT, using a memory relaxation time τ_AT_^RDC^, and to allow time-averaging of RDCs, using a memory relaxation time τ_D_^RDC^. In the present study only the standard, commonly used alignment-tensor approach is applied, i.e., without any time-averaging (τ_AT_^RDC^ = τ_D_^RDC^ = 0 ps).

### 2.2 Choice of Restraining Function in the HRS Method

A simple function to apply RDC-restraining to the two-atom magnetic-field vector motion, which is continuous with a continuous derivative, is a quadratic one with a flat bottom of size 2Δ*D* ^fb^ that allows for a penalty-free range of deviations ±Δ*D* ^fb^ of the instantaneous *D*_*k*_ value or the average ⟨*D*_*k*_⟩-value from its target (measured) value *D*_*k*_^0^. In the SD simulation of the magnetic-field vector the time average, ${\overset{®}{D_{k}\;}}^{t}$ ≡ ⟨*D*_*k*_⟩_*t*_, is used. For large deviations of ${\overset{®}{D_{k}\;}}^{t}$ from *D*_*k*_^0^, beyond Δ*D* ^fb^ + Δ*D* ^h^, the restraining function is chosen to be linear in order to avoid large restraining forces and energies. In analogy to the flat-bottom restraining function for NOEs, ^3^ *J*-couplings and *S* ^2^ order parameters,^18^ the corresponding function for RDCs would be for ${\overset{®}{D_{k}\;}}^{t}$ > *D*_*k*_^0^

8a $$\begin{array}{l} V_{k}^{RDC}\left({\overset{®}{D_{k}\left({\mathrm{r⃗}}^{N}\right)\;}}^{t};D_{k}^{0},K^{RDC},\Delta D^{fb},\Delta D^{h}\right)=\frac{1}{2}K^{RDC}{\left({\overset{®}{D_{k}\left({\mathrm{r⃗}}^{N}\right)\;}}^{t}-D_{k}^{0}-\Delta D^{fb}\right)}^{2}\\ H\left({\overset{®}{D_{k}\left({\mathrm{r⃗}}^{N}\right)\;}}^{t};D_{k}^{0}+\Delta D^{fb}\right)\left(1-H\left({\overset{®}{D_{k}\left({\mathrm{r⃗}}^{N}\right)\;}}^{t};D_{k}^{0}+\Delta D^{fb}+\Delta D^{h}\right)\right)\\ +K^{RDC}\left({\overset{®}{D_{k}\left({\mathrm{r⃗}}^{N}\right)\;}}^{t}-D_{k}^{0}-\Delta D^{fb}-\frac{1}{2}\Delta D^{h}\right)\Delta D^{h}H\left({\overset{®}{D_{k}\left({\mathrm{r⃗}}^{N}\right)\;}}^{t};D_{k}^{0}+\Delta D^{fb}+\Delta D^{h}\right) \end{array}$$

and for ${\overset{®}{D_{k}\;}}^{t}$ < *D*_*k*_^0^

8b $$\begin{array}{l} V_{k}^{RDC}\left({\overset{®}{D_{k}\left({\mathrm{r⃗}}^{N}\right)\;}}^{t};D_{k}^{0},K^{RDC},\Delta D^{fb},\Delta D^{h}\right)=\frac{1}{2}K^{RDC}{\left({\overset{®}{D_{k}\left({\mathrm{r⃗}}^{N}\right)\;}}^{t}-D_{k}^{0}+\Delta D^{fb}\right)}^{2}\\ \left(1-H\left({\overset{®}{D_{k}\left({\mathrm{r⃗}}^{N}\right)\;}}^{t};D_{k}^{0}-\Delta D^{fb}\right)\right)H\left({\overset{®}{D_{k}\left({\mathrm{r⃗}}^{N}\right)\;}}^{t};D_{k}^{0}-\Delta D^{fb}-\Delta D^{h}\right)\\ -K^{RDC}\left({\overset{®}{D_{k}\left({\mathrm{r⃗}}^{N}\right)\;}}^{t}-D_{k}^{0}+\Delta D^{fb}+\frac{1}{2}\Delta D^{h}\right){\Delta D}^{h}\left(1-H\left({\overset{®}{D_{k}\left({\mathrm{r⃗}}^{N}\right)\;}}^{t};D_{k}^{0}-\Delta D^{fb}-\Delta D^{h}\right)\right) \end{array}$$

with the Heaviside step function *H* (*x*; *x*_0_) defined by

9a $$H\left(x;x_{0}\right)=0,\mathrm{for},x<x_{0}$$

9b $$H\left(x;x_{0}\right)=1,\mathrm{for},x\geq x_{0}$$

The corresponding force on ${\overset{®}{D_{k}\;}}^{t}$ , that is, the negative of the derivative of *V*_*k*_^RDC^ with respect to ${\overset{®}{D_{k}\;}}^{t}$ is then for ${\overset{®}{D_{k}\;}}^{t}$ > *D*_*k*_^0^

10a $$f^{RDC}\left({\overset{®}{D_{k}\left({\mathrm{r⃗}}^{N}\right)\;}}^{t}\right)=0,\mathrm{for},{\overset{®}{D_{k}\;}}^{t}<D_{k}^{0}+\Delta D^{fb}$$

10b $$f^{RDC}\left({\overset{®}{D_{k}\left({\mathrm{r⃗}}^{N}\right)\;}}^{t}\right)=-K^{RDC}\left({\overset{®}{D_{k}\left({\mathrm{r⃗}}^{N}\right)\;}}^{t}-D_{k}^{0}-\Delta D^{fb}\right),\mathrm{for},D_{k}^{0}+\Delta D^{fb}\leq {\overset{®}{D_{k}\;}}^{t}\leq D_{k}^{0}+\Delta D^{fb}+\Delta D^{h}$$

10c $$f^{RDC}\left({\overset{®}{D_{k}\left({\mathrm{r⃗}}^{N}\right)\;}}^{t}\right)=-K^{RDC}\Delta D^{h},\mathrm{for},{\overset{®}{D_{k}\;}}^{t}>D_{k}^{0}+\Delta D^{fb}+\Delta D^{h}$$

and for ${\overset{®}{D_{k}\;}}^{t}$ < *D*_*k*_^0^

10d $$f^{RDC}\left({\overset{®}{D_{k}\left({\mathrm{r⃗}}^{N}\right)\;}}^{t}\right)=0,\mathrm{for},{\overset{®}{D_{k}\;}}^{t}<D_{k}^{0}-\Delta D^{fb}$$

10e $$f^{RDC}\left({\overset{®}{D_{k}\left({\mathrm{r⃗}}^{N}\right)\;}}^{t}\right)=-K^{RDC}\left({\overset{®}{D_{k}\left({\mathrm{r⃗}}^{N}\right)\;}}^{t}-D_{k}^{0}+\Delta D^{fb}\right),\mathrm{for},D_{k}^{0}-\Delta D^{fb}-\Delta D^{h}\leq {\overset{®}{D_{k}\;}}^{t}\leq D_{k}^{0}-\Delta D^{fb}$$

10f $$f^{RDC}\left({\overset{®}{D_{k}\left({\mathrm{r⃗}}^{N}\right)\;}}^{t}\right)=+K^{RDC}\Delta D^{h},\mathrm{for},{\overset{®}{D_{k}\;}}^{t}<D_{k}^{0}-\Delta D^{fb}-\Delta D^{h}$$

To obtain the force on the two atoms or particles *h*_1_ and *h*_2_ representing the magnetic-field vector, these expressions are multiplied by $\partial {\overset{®}{D_{k}\left(r^{N}\left(t\right)\right)\;}}^{t}/\partial {\mathrm{r⃗}}_{h}\left(t\right)$ , the derivative of ${\overset{®}{D_{k}\;}}^{t}$ with respect to *r⃗*_*h*_(*t*).^14^

For a set of *N*_RDC_ *D*_*k*_ values we have

11 $$V^{RDC}\left({\overset{®}{D\left({\mathrm{r⃗}}^{N}\right)}}^{t}\right)=\sum_{k=1}^{N_{RDC}}V_{k}^{RDC}\left(\overset{®}{D_{k}{\left({\mathrm{r⃗}}^{N}\right)}^{t}\;};D_{k}^{0},K^{RDC,mfv},\Delta D^{fb},\Delta D^{h}\right)$$

Here, *r⃗* ^*N*^ ≡ (*r⃗*_1_,*r⃗*_2_,···,*r⃗*_*N*_) are the coordinates of the protein, and *K* ^RDC*,mfv*^ is the restraining force constant.

### 2.3 Accounting for Time Averaging in the HRS Method

An RDC is intrinsically an average (over the rotation of the molecule) quantity, ⟨*D*_*k*_⟩, that cannot be linked to a single configuration. When restraining an averaged quantity in an SD or MD simulation, the averaging cannot be over the whole simulation period *t* because the contribution of the configuration at time *t* to the average would tend to zero for *t* approaching infinity, which implies that the restraining force, which can be applied only to the current configuration at time *t*, would also tend to zero for *t* approaching infinity. For this reason, instead of using the time average ${\overset{®}{D_{k}\;}}^{t}$ ≡ ⟨*D*_*k*_⟩_*t*_, an exponential memory function is used in the time average, ${\overset{®}{D_{k}\;}}^{t,\exp}$ , that is used in the restraining function^19,20^

12 $${\overset{®}{D_{k}\left({\mathrm{r⃗}}^{N}\left(t\right)\right)\;}}^{t}={\left(\tau_{D}^{RDC}\left(1-\exp\left(-t/\tau_{D}^{RDC}\right)\right)\right)}^{-1}\int_{0}^{t}\exp\left(-\left(t-t^{\prime }\right)/\tau_{D}^{RDC}\right)D_{k}\left({\mathrm{r⃗}}^{N}\left(t^{\prime }\right)\right)dt^{\prime }$$

where τ_D_^RDC^ is the memory relaxation time, which determines the averaging time period. As discussed before, the averaging over the two time-dependent factors in eq 2 may be well approximated, due to the different relevant time scales, by a separation of the averaging, see eq 6, so

13 $${\overset{®}{D_{k}\left({\mathrm{r⃗}}^{N}\left(t\right)\right)\;}}^{t}=D_{k}^{c}{\overset{®}{{\left(\frac{r^{0}}{r_{k_{1}k_{2}}\left(t\right)}\right)}^{3}\;}}^{t}{\overset{®}{\left(\frac{1}{2}\left(3\;\cos^{2}\left(\theta_{k}\left(t\right)\right)-1\right)\right)}}^{t}=D_{k}^{c}{\overset{®}{R_{k}\left(t\right)\;}}^{t}{\overset{®}{P_{k}\left(t\right)\;}}^{t}$$

Since the magnetic-field vector is represented by two atoms connected by a rigid bond and the RDC-restraining force on it only depends on the angles θ_*k*_, only the time-averaged quantity ${\overset{®}{P_{k}\left(t\right)\;}}^{t}$ in eq 13 is used either as a standard time average ${\overset{®}{P_{k}\left(t\right)\;}}^{t}$ or as an exponentially damped one

14 $${\overset{®}{P_{k}\left({\mathrm{r⃗}}^{N}\left(t\right)\right)\;}}^{t}={\left(\tau_{\theta }^{RDC,mfv}\left(1-\exp\left(-t/\tau_{\theta }^{RDC,mfv}\right)\right)\right)}^{-1}\int_{0}^{t}\exp\left(-\left(t-t^{\prime }\right)/\tau_{\theta }^{RDC,mfv}\right)P_{k}\left({\mathrm{r⃗}}^{N}\left(t^{\prime }\right)\right)dt^{\prime }$$

with **τ**_θ_^RDC,*mfv*^ its memory relaxation time.

### 2.4 Calculation of the Restraining Force in the HRS Method

The restraining force on atom *h* of the magnetic-field vector with position vector *r⃗*_*h*_ is, using $f^{RDC}\left({\overset{®}{D_{k}\left({\mathrm{r⃗}}^{N}\right)\;}}^{t}\right)$ from eq 10a

15 $${\mathrm{f⃗}}_{h}\left(t\right)=-\frac{\partial V^{RDC}\left(t\right)}{\partial {\mathrm{r⃗}}_{h}\left(t\right)}=\sum_{k=1}^{N_{RDC}}f^{RDC}\left({\overset{®}{D_{k}\left({\mathrm{r⃗}}^{N}\right)\;}}^{t}\right)\frac{\partial {\overset{®}{D_{k}\left(r^{N}\left(t\right)\right)\;}}^{t}}{\partial {\mathrm{r⃗}}_{h}\left(t\right)}$$

or in terms of the function *P*_*k*_

16 $${\mathrm{f⃗}}_{h}\left(t\right)=\sum_{k=1}^{N_{RDC}}f^{RDC}\left(D_{k}^{c}R_{k}\left(r_{k_{1}k_{2}}\right){\overset{®}{P_{k}\left({\mathrm{r⃗}}^{N}\left(t\right)\right)\;}}^{t}\right)D_{k}^{c}R_{k}\left(r_{k_{1}k_{2}}\right)\left\{\frac{\partial {\overset{®}{P_{k}\left({\mathrm{r⃗}}^{N}\left(t\right)\right)\;}}^{t}}{\partial P_{k}\left({\mathrm{r⃗}}^{N}\left(t\right)\right)}\frac{\partial P_{k}\left({\mathrm{r⃗}}^{N}\left(t\right)\right)}{\partial {\mathrm{r⃗}}_{h}\left(t\right)}\right\}$$

In a simulation, the atomic configurations are separated by a time step or interval Δ*t*, so in discretized form we have for the *n*th time step for the exponentially damped time-averaged quantity in eq 16

17 $${\overset{®}{P_{k}\left(n\Delta t\right)\;}}^{n\Delta t,\exp}=P_{k}\left(n\Delta t\right)\left(1-\exp\left(-\Delta t/\tau_{\theta }^{RDC,mfv}\right)\right)+\exp\left(-\Delta t/\tau_{\theta }^{RDC,mfv}\right){\overset{®}{P_{k}\left(\left(n-1\right)\Delta t\right)\;}}^{\left(n-1\right)\Delta t,\exp}$$

and using this equation

18 $$\frac{\partial {\overset{®}{P_{k}\left(n\Delta t\right)\;}}^{t}}{\partial P_{k}\left(n\Delta t\right)}=1-\exp\left(-\Delta t/\tau_{\theta }^{RDC,mfv}\right)$$

Generally, the memory relaxation time **τ**_θ_^RDC,*mfv*^ is chosen to be much longer than the SD integration time step Δ*t*

$$\Delta t\ll \tau_{\theta }^{RDC,mfv}\ll t^{SD}$$ 19

where *t* ^SD^ is the length of the SD simulation. This means that the derivative eq 18 can be approximated by Δ*t*/**τ**_θ_^RDC,*mfv*^.

For the derivative of *P*_*k*_ with respect to the position of atom *h* of the magnetic-field vector using eq 5 and the definition

20 $$\theta_{k}\equiv \arccos\left(\frac{x_{h_{1}h_{2}}x_{k_{1}k_{2}}+y_{h_{1}h_{2}}y_{k_{1}k_{2}}+z_{h_{1}h_{2}}z_{k_{1}k_{2}}}{r_{h_{1}h_{2}}r_{k_{1}k_{2}}}\right)$$

we find

21a $$\frac{\partial P_{k}\left({\mathrm{r⃗}}^{N}\left(t\right)\right)}{\partial x_{h}\left(t\right)}=\left(\delta_{hh_{1}}-\delta_{hh_{2}}\right)3\left(\frac{x_{h_{1}h_{2}}\left(t\right)x_{k_{1}k_{2}}\left(t\right)+y_{h_{1}h_{2}}\left(t\right)y_{k_{1}k_{2}}\left(t\right)+z_{h_{1}h_{2}}\left(t\right)z_{k_{1}k_{2}}\left(t\right)}{r_{h_{1}h_{2}}r_{k_{1}k_{2}}}\right)\left(\frac{x_{k_{1}k_{2}}\left(t\right)}{r_{h_{1}h_{2}}r_{k_{1}k_{2}}}\right)$$

21b $$\frac{\partial P_{k}\left({\mathrm{r⃗}}^{N}\left(t\right)\right)}{\partial y_{h}\left(t\right)}=\left(\delta_{hh_{1}}-\delta_{hh_{2}}\right)3\left(\frac{x_{h_{1}h_{2}}\left(t\right)x_{k_{1}k_{2}}\left(t\right)+y_{h_{1}h_{2}}\left(t\right)y_{k_{1}k_{2}}\left(t\right)+z_{h_{1}h_{2}}\left(t\right)z_{k_{1}k_{2}}\left(t\right)}{r_{h_{1}h_{2}}r_{k_{1}k_{2}}}\right)\left(\frac{y_{k_{1}k_{2}}\left(t\right)}{r_{h_{1}h_{2}}r_{k_{1}k_{2}}}\right)$$

21c $$\frac{\partial P_{k}\left({\mathrm{r⃗}}^{N}\left(t\right)\right)}{\partial z_{h}\left(t\right)}=\left(\delta_{hh_{1}}-\delta_{hh_{2}}\right)3\left(\frac{x_{h_{1}h_{2}}\left(t\right)x_{k_{1}k_{2}}\left(t\right)+y_{h_{1}h_{2}}\left(t\right)y_{k_{1}k_{2}}\left(t\right)+z_{h_{1}h_{2}}\left(t\right)z_{k_{1}k_{2}}\left(t\right)}{r_{h_{1}h_{2}}r_{k_{1}k_{2}}}\right)\left(\frac{z_{k_{1}k_{2}}\left(t\right)}{r_{h_{1}h_{2}}r_{k_{1}k_{2}}}\right)$$

and

22 $$\frac{\partial P_{k}}{\partial {\mathrm{r⃗}}_{h_{1}}}=-\frac{\partial P_{k}}{\partial {\mathrm{r⃗}}_{h_{2}}}$$

### 2.5 Calculation of RDCs Using the HRS Method

The calculation of RDC values is straightforward using ${\overset{®}{P_{k}\left(t\right)\;}}^{t}$ in eq 6 and averaging over SD trajectory structures. In the RDC restraining simulations, the RDC-restraining forces on the magnetic-field vector are calculated at every time step using an exponential damping factor in the average, using ${\overset{®}{P_{k}\left(t\right)\;}}^{t,\exp}$ for ${\overset{®}{P_{k}\left(t\right)\;}}^{t}$ in eq 16. These two types of RDC values, calculated from ${\overset{®}{P_{k}\left(t\right)\;}}^{t,\exp}$ on the one hand and from ${\overset{®}{P_{k}\left(t\right)\;}}^{t}$ on the other, will differ. In order to analyze all magnetic-field vector trajectories in the same way, ${\overset{®}{P_{k}\left(t\right)\;}}^{t}$ or rather ${\overset{®}{D_{k}\left(t\right)\;}}^{t}$ is used when reporting RDC values calculated from the trajectories.

When using the rotational sampling method in the presence of a single RDC restraint, the orientation distribution of the molecule may show two peaks at the two magic angles.^10^ Instead of reducing the rotationally averaged DC by sampling the complete 180° range of angles between the magnetic-field direction and some axis or an RDC vector in the molecule, such a reduction may more easily be achieved by sampling angles close to the magic-angle values. When applying more than one RDC restraint, of which the bond vectors appear to have different orientations in the molecule, this artifact may disappear.^10^ However, in case the various target RDC values of a set of more than a few RDC restraints are inconsistent with a molecular structure, the rotational-sampling algorithms may also lead to an enhanced sampling of orientation angles around the magic-angle values, i.e., to restricted sampling of the rotational degrees of freedom of the molecule.^14^ Thus, the orientation distribution of the molecule resulting from RDC-restraining should not be dominated by angle values around the magic angles.

## 3 Comparison of AT, MRS, and HRS Methods

An RDC is defined as an average over an orientationally biased rotational distribution of the molecule. At the microscopic level of resolution, the real biasing forces and their influence on the rotational distribution are unknown. Unfortunately, the real biased orientation distribution cannot be measured experimentally. Because of the lack of information on the orientation distribution at the microscopic level of resolution, the real biased orientation distribution cannot be faithfully approximated using modeling on a computer. As a consequence, currently, the only approach to calculate RDC values for a structure of a molecule is (i) by postulating the form of the (biased) orientation distribution (AT method) or (ii) by generating a (biased) orientation distribution by simulation of the rotational motion of the molecule (MRS method) or of the rotational motion of a magnetic-field vector (HRS method), while minimizing the difference between a set of RDC values calculated from the (biased) orientation distribution and the corresponding (measured) target RDC values for a chosen set of RDCs and a chosen molecular structure. This procedure makes calculated RDC values vary with a variation of the set of target RDC values and with the structure used in their calculation.

In the AT method, the orientation distribution is a linear combination of the five spherical harmonic functions of order 2 and it depends on the five coefficients of the linear combination that are varied to minimize the difference between calculated and target RDC values. This limits the variability of the orientation distribution. This is not surprising, considering the procedure to determine the coefficients of the linear combination and thus the alignment-tensor elements. Contributions from spherical harmonics of order different from 2, tensor ranks other than 2, do not play a role in the measurement of RDCs. This implies that using the AT formalism, the orientation distribution of the molecule is likely to contain only components that are representable with spherical harmonics of order 2. In contrast, MD or SD simulation may generate a more general orientation distribution, which can be reproduced^13^ using the molecular rotational-sampling MRS method for structure determination or refinement of molecules based on RDC data. Thus, even in the case the RDC target values are reproduced in AT calculations, these values do not necessarily represent the underlying nonuniform rotational distribution of the molecule.

In the MRS and HRS methods, the scaling down from the kHz to the Hz size is not achieved by scaling down the size of the coefficients of the linear combination of the five spherical harmonic functions of order 2, as in the AT method, but by averaging over sufficiently many orientation directions of the molecule (MRS method) or of the magnetic-field vector (HRS method) that are simulated. This requires many (opposite) directions to be simulated, so very long simulations are needed in order to reach a reduction of the average (RDC) by a factor of 1000 to 10,000. Yet, the representation of the orientation distribution of the molecule (MRS) or of the magnetic-field vector (HRS) in terms of delta functions dependent on time, that is, individual trajectory directions (MRS: molecule; HRS: magnetic field) generated in MD (MRS) or SD (HRS) simulation, allows a larger degree of nonuniformity or anisotropy when varying the orientation distribution to minimize the difference between calculated and target RDC values, larger than in the AT method.

The two mentioned features characteristic for RDCs, (i) being sensitive to the form and extent of the unknown and immeasurable orientation distribution which is varied to bring calculated RDC values close to given (experimental) target RDC values, and (ii) being the result of an averaging over an orientation distribution that scales down the averaged DC values over 4 orders of magnitude, leading to RDC values, while the orientation distribution contains different molecular structures, makes calculated RDC values sensitive to (i) the size of the set of RDC target values used, (ii) their consistency with the set of molecular structures present in the measurement of RDC values or used in the calculation of RDC values, and (iii) the particular structure or the variation of structures used in the calculation.

In the conventional AT method and when no simulation of the molecule is allowed in the HRS method (only magnetic-field vector SD simulation), there is no influence of molecular structural variation on the calculated RDC values. The (anisotropic) orientation distribution is generated for a single molecular structure. Inclusion of a variation of molecular structures when calculating RDC values is done differently in the three methods. In the AT method it can be done by time-averaging the five coefficients *a*_m_ that determine the five alignment-tensor elements or by time-averaging the calculated RDC values^13^ in a simulation or by averaging over different molecular conformations. This, of course, brings calculated RDC values closer to the target ones. In the MRS method, variation of the molecular structure is intrinsically present. In the HRS method variation of the molecular structure is included when *K* ^RDC,*msy*^ > 0 and when the intramolecular motion is simulated.

When analyzing the effects of the various factors, assumptions, and approximations influencing the calculated RDC values, (i) method (AT, MRS, HRS), (ii) set of target RDC values used, (iii) molecular model or force field used, and (iv) internal motions of the molecule, one would like to vary one factor at a time, or even better, eliminate, if possible, other factors from the analysis. The approach used here, based on single X-ray structures, allows elimination of force-field or molecular model effects and effects of structural variation. The use of different single X-ray structures offers an impression of the effects of structural variation used in a calculation of RDC values.

Each of the three mentioned methods has advantages and disadvantages:

1. The AT method postulates the form of the orientation distribution and thus does not require any sampling of the molecular orientations or configurations. The computational effort is very low. It can be applied not only to a single molecular structure but also to each structure of a molecular trajectory while time-averaging.^13^ The latter, of course, improves the agreement between calculated and target RDC values but is physically a bit inconsistent. It allows a wholly new and thus possibly rather different orientation distribution for structures of a trajectory that are very close, of the order of fs, in time, and there is no physically correct coupling between rotational and internal motions of the molecule.
2. The MRS method approximates the physical basis of RDCs best. It allows coupling between rotational and internal motions of the molecule. However, to obtain well-converged RDC values, it requires currently prohibitively long MD simulation times for explicitly solvated large molecules such as proteins because of the necessary reduction of the RDC values by more than 3 orders of magnitude as a result of the averaging. It also introduces a force-field or molecular-model (including solvent representation) dependence of the calculated RDC values.
3. In comparison to the MRS method, the HRS method reduces the computational effort much by simulating the rotational motion of a magnetic-field vector instead of the whole molecule, but at the expense of reducing the coupling between rotational and internal motion of the molecule. Yet, this method has an advantage. If only the rotational motion of the magnetic-field vector is simulated, *K* ^RDC,*msy*^ = 0 and *N*_*msy*_ = 1, i.e. no simulation of intramolecular motion, there is no force-field or molecular-model (including solvent representation) dependence of the calculated RDC values, and RDC values can be calculated for a single structure.

Regarding the current investigation, it was decided to refrain from simulation of the motion of the molecule in order to avoid distortive effects due to the molecular model or force field that must be used in a simulation.

## 4 Molecular Structures, Magnetic-Field Simulation Setup, and Analysis

The calculations and simulations were performed using the GROMOS simulation software package.^21–26^ When realistic molecular systems are treated, the use of Standard International (SI) units is recommended. Apart from restrictions when storing or printing data in nonexponential format, the GROMOS programs are independent of the chosen units. The units are defined by the ones used for the physical constants and atomic and molecular quantities to be specified in the PHYSICALCONSTANTS block^21^ in the GROMOS data files.^21^ It is recommended that the following basic units be used: nanometer (nm) for length, atomic mass unit (u) for mass, picosecond (ps) for time, Kelvin (K) for temperature, and electronic charge (e) for charge. These basic units then determine the units of other quantities, e.g., kJ/mol for energy, kJ/(mol nm) for force, kJ/(mol nm^3^) for pressure, and THz for frequency. If, for example, the non-GROMOS-recommended unit Hz instead of THz is to be used as the input unit for dipolar couplings and kJ mol^–1^ Hz^–2^ for the RDC-restraining force constant, the scaling factor 10^12^ should be specified in the RDCRESTRAINTS block.^21^

### 4.1 Structures of the Protein HEWL

Five X-ray crystal structures, differing in the crystal unit cell and temperature, were used:

1. Structure “4LZT” of the Protein Data Bank (PDB),^27^ derived from a triclinic unit cell at 0.095 nm resolution (space group *P*1), *T* = 295 K, pH = 4.5, refined by using the SHELXL-96 software.^28^ It contains multiple side-chain conformations for 8 residues.
2. Structure “2VB1” of the Protein Data Bank (PDB),^27^ derived from a triclinic unit cell at 0.065 nm resolution (space group *P*1), *T* = 100 K, pH = 4.7, refined by using the SHELXL-97 software.^28^ It contains multiple side-chain conformations for 46 residues.
3. Structure “1IEE” of the Protein Data Bank (PDB),^27^ derived from a tetragonal unit cell at 0.094 nm resolution (space group *P*4_3_2_1_2), *T* = 110 K, pH = 4.5, refined by using the SHELXL-97 software.^28^ It contains multiple side-chain conformations for 33 residues.
4. Structure “1AKI” of the Protein Data Bank (PDB),^27^ derived from an orthorhombic unit cell at 0.15 nm resolution (space group *P*2_1_2_1_2_1_), *T* = 298 K, pH = 4.48, refined by using the X-PLOR software.^29^ It contains no multiple side-chain conformations.
5. Structure “1HF4” of the Protein Data Bank (PDB),^27^ derived from a monoclinic unit cell (space group *P*2_1_) at 0.145 nm resolution, *T* = 293 K, pH = 4.5, and refined by using the X-PLOR software.^29^ Of the two structures present in the asymmetric unit, the first one, “1HF4a”, was chosen. It contains multiple side-chain conformations for 2 residues.

For the five X-ray structures, the side-chain conformation with the highest occupancy was chosen. The positions of the H^N^ hydrogens bound to the backbone N atoms were generated using the GROMOS software,^21^ as were the positions of the virtual H^α^ hydrogens bound to the backbone C^α^ atoms. The bond lengths relevant to the calculation of the RDC values are *b* ^0^(N–H^N^) = 0.1 nm, *b* ^0^(C^α^–H^α^) = 0.109 nm, *b* ^0^(C^α^–C′) = 0.153 nm, and *b* ^0^(C′–N) = 0.133 nm.^21^ Using *r* ^0^ = 0.1 nm, one finds for *R*_*k*_(*t*), see eq 4: *R*_N–HN_(*t*) = 1.0, *R*_Cα–Hα_(*t*) = 0.7722, *R*_Cα–C′_(*t*) = 0.2792, and *R*_C′–N_(*t*) = 0.4251.

### 4.2 Model for the Magnetic-Field Vector SD Simulations in the HRS Method

A most simple model for the two-particle magnetic-field vector is a molecule of two united atoms, ethane,^10^ with a rigid bond. The masses of the two atoms are 15.035 u (a CH_3_ united atom in GROMOS), and the length of the bond is 0.153 nm. The length of the vector is kept constant using the SHAKE algorithm^30^ with a relative geometric precision of 10^–4^. The only potential energy term used is the RDC-restraining one (eqs 8a and 11), so the motion of the vector is determined by the Langevin equation^31^ and the RDC-restraining forces. A friction coefficient γ_*i*_^*mfv*^ = 2.4 ps^–1^ is used for the two atoms, because this value optimizes the rotational sampling of the magnetic-field vector.^10^

The initial position of the magnetic-field vector is arbitrary, e.g., along the *z*-axis. The *mfv* SD simulations of the magnetic-field vector *r⃗*_*h*_1_ *h*_2__ were carried out at 308 K and with a time step of Δ*t* = 2 fs. The three *mfv* SD simulations of 5 × 10^6^ time steps of 2 fs each yield 30 ns of sampling.

### 4.3 Measured RDC Values Serving as Target RDC Values

For HEWL at pH = 3.8 and *T* = 308 K, five different sets of experimentally determined RDC values are available^16^ (Figure 1):

1. Two sets of 38 and 39 ^13^C^α^–^1^H^α^ RDCs, Tables 4.2 and 4.3 of ref 16 for 59 residues, see Table 1, with values ranging from −30.56 Hz to +21.03 Hz. The value of *D*_*k*_^*c*^ (^13^C–^1^H) = −46.66 kHz (with *r* ^0^ = 0.109 nm). The set of 59 RDCs is indicated as set RDC_*CAH59*_.
2. One set of 101 ^15^N–^1^H^N^ RDCs, Table 4.1 of ref 16 see Table 2, with values ranging from −8.88 Hz to +16.06 Hz. The value of *D*_*k*_^*c*^ (^15^N–^1^H) = +24.36 kHz (with *r* ^0^ = 0.1 nm). This set is indicated as set RDC_*NH101*_.
3. One set of 97 ^13^C^α^–^13^C′ RDCs, Table 4.4 of ref 16, see Table 3, with values ranging from −3.36 to +1.95 Hz. The value of *D*_*k*_^*c*^ (^13^C–^13^C) = −4.241 kHz (with *r* ^0^ = 0.153 nm). This set is indicated as set RDC_*CAC97*_.
4. One set of 45 ^13^C′–^15^N RDCs, Table 4.5 of ref 16, see Table 4, with values ranging from −1.41 to +2.41. The value of *D*_*k*_^*c*^ (^13^C–^15^N) = +2.606 kHz (with *r* ^0^ = 0.133 nm). This set is indicated as set RDC_*CN45*_.

**Figure 1:**
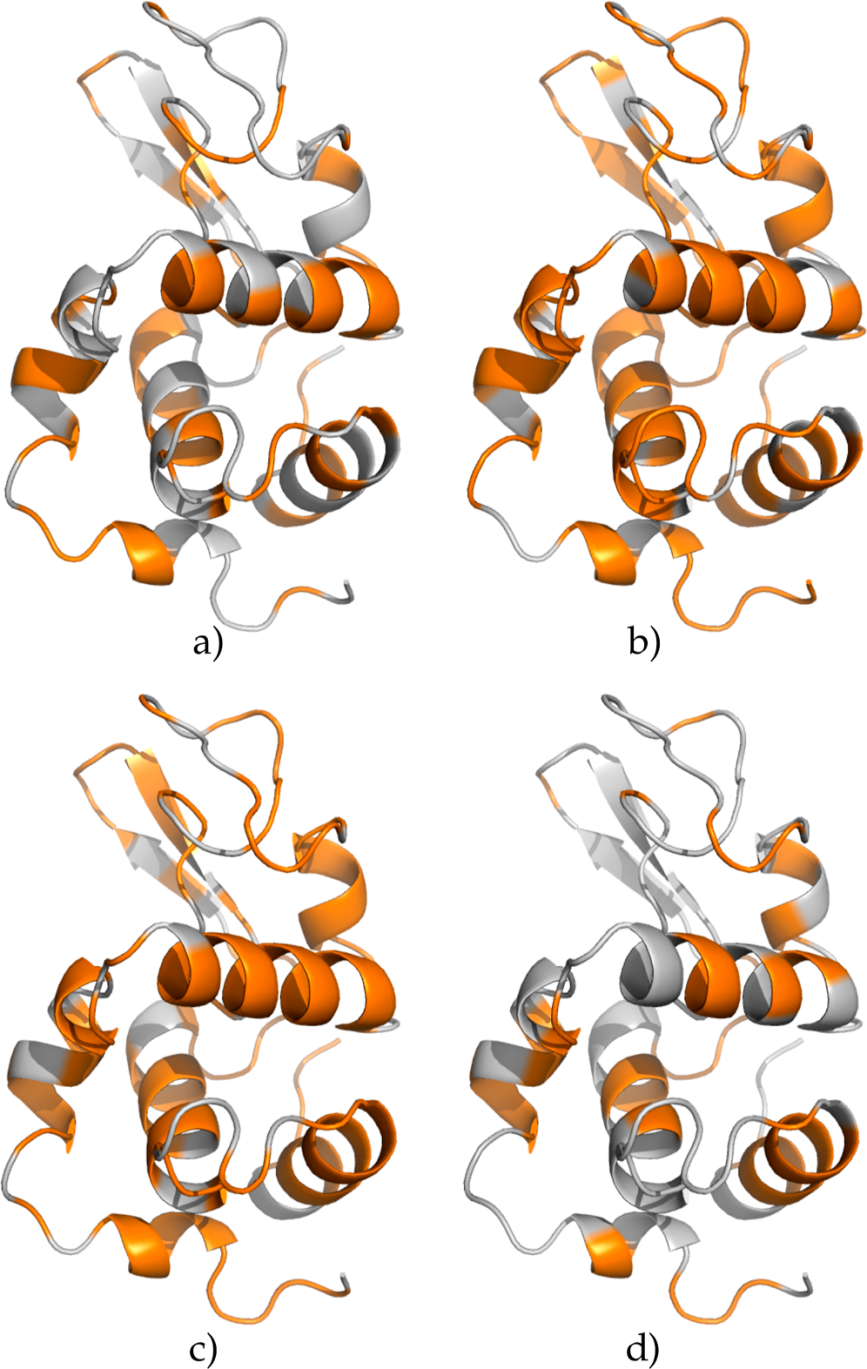
Ribbon picture of the 2VB1 X-ray structure of HEWL residues for which measured (backbone) RDC values are available are colored orange. The four panels show four types (sets) of measured RDCs: (a) set RDC_*CAH59*_ (59 ^13^C^α^–^1^H^α^ RDCs); (b) set RDC_*NH101*_ (101 ^15^N–^1^H^N^ RDCs); (c) set RDC_*CAC97*_ (97 ^13^C^α^–^13^C′ RDCs); and (d) set RDC_*CN45*_ (45 ^13^C′–^15^N RDCs).

**Table 1:**
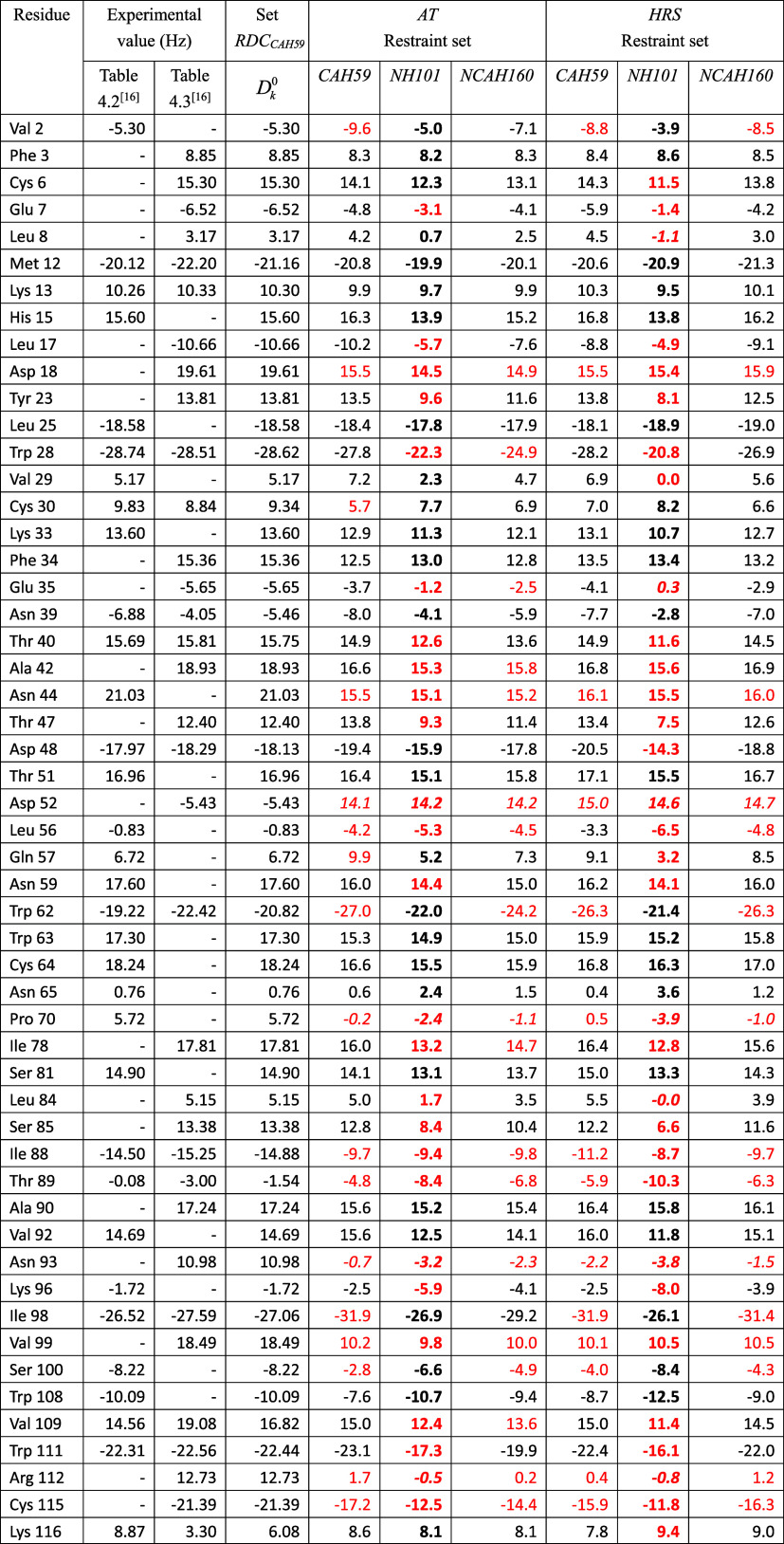

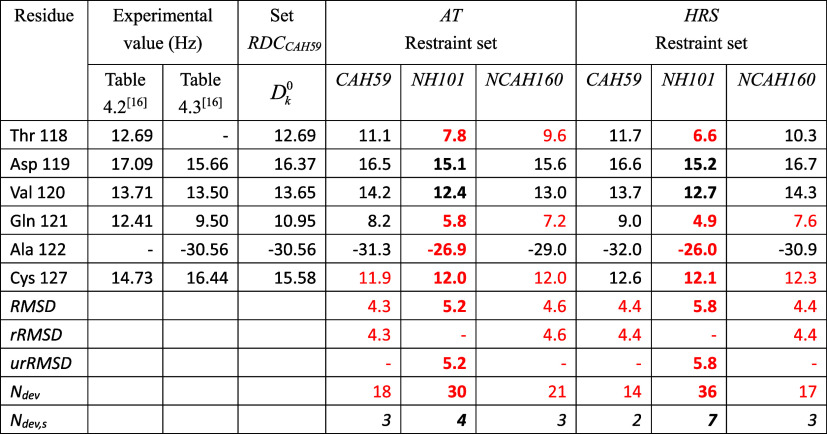
^13^C^α^–^1^H^α^ RDC Values (38, 39) in Hz for HEWL, as Obtained from NMR Measurements at 308 K and pH = 3.8 Using Two Different Pulse Sequences as Given in Tables 4.2 and 4.3 of ref 16 and as Calculated Using Three Different Sets of RDC Restraints (RDC_*CAH59*_, RDC_*NH101*_, RDC_*NCAH160*_) for the X-ray Structure 4LZT by Applying the Alignment-Tensor Method (AT: τ_D_^RDC^ = 0, τ_AT_^RDC^ = 0)^13^ or the HRS (*K* ^RDC,*msy*^ = 0) Method,^14^ *K* ^RDC,*mfv*^ = 100 kJ mol^–1^ Hz^–2^, τ_θ_^RDC,*mfv*^ = 10 ns, in 30 ns of SD Simulation of the Magnetic-Field Vector^a^

a The (RDC restraint) set RDC_*CAH59*_ contains the RDC values *D*_*k*_^0^ given in the fourth column. The RDC restraint set RDC_*NH101*_ contains the RDC values *D*_*k*_^0^ given in the second column of Table 2. The RDC restraint set RDC_*NCAH160*_ is obtained by combining the sets of RDC restraints RDC_*CAH59*_ and RDC_*NH101*_. In case two experimental values are available, the *D*_*k*_^0^ RDC values used in the calculations are the average of the two experimental values. The values for the RDCs that are not part of the (sub)­set of RDC restraints applied are in bold. RMSD: Root-mean-square difference (RMSD) between calculated *D*_*k*_ and *D*_*k*_^0^ RDC values calculated over all, *mfv*-restrained and unrestrained, RDCs. rRMSD: RMSD values calculated over the particular (sub)­set of *mfv*-restrained RDCs. urRMSD: RMSD values calculated over the unrestrained RDCs. Deviations of RDC values *D*_*k*_1_ *k*_2__ (AT) or averaged ⟨*D*_*k*_1_ *k*_2__⟩_*t*_^*mfv*^ (HRS) from the *D*_*k*_^0^ values larger than 3 Hz are in red. *N*_dev_: Number of such deviations. *N*_dev,s_: Number of RDCs for which the calculated *D*_*k*_ and the *D*_*k*_^0^ values have a different sign. These RDC values are in italics.

**Table 2:**
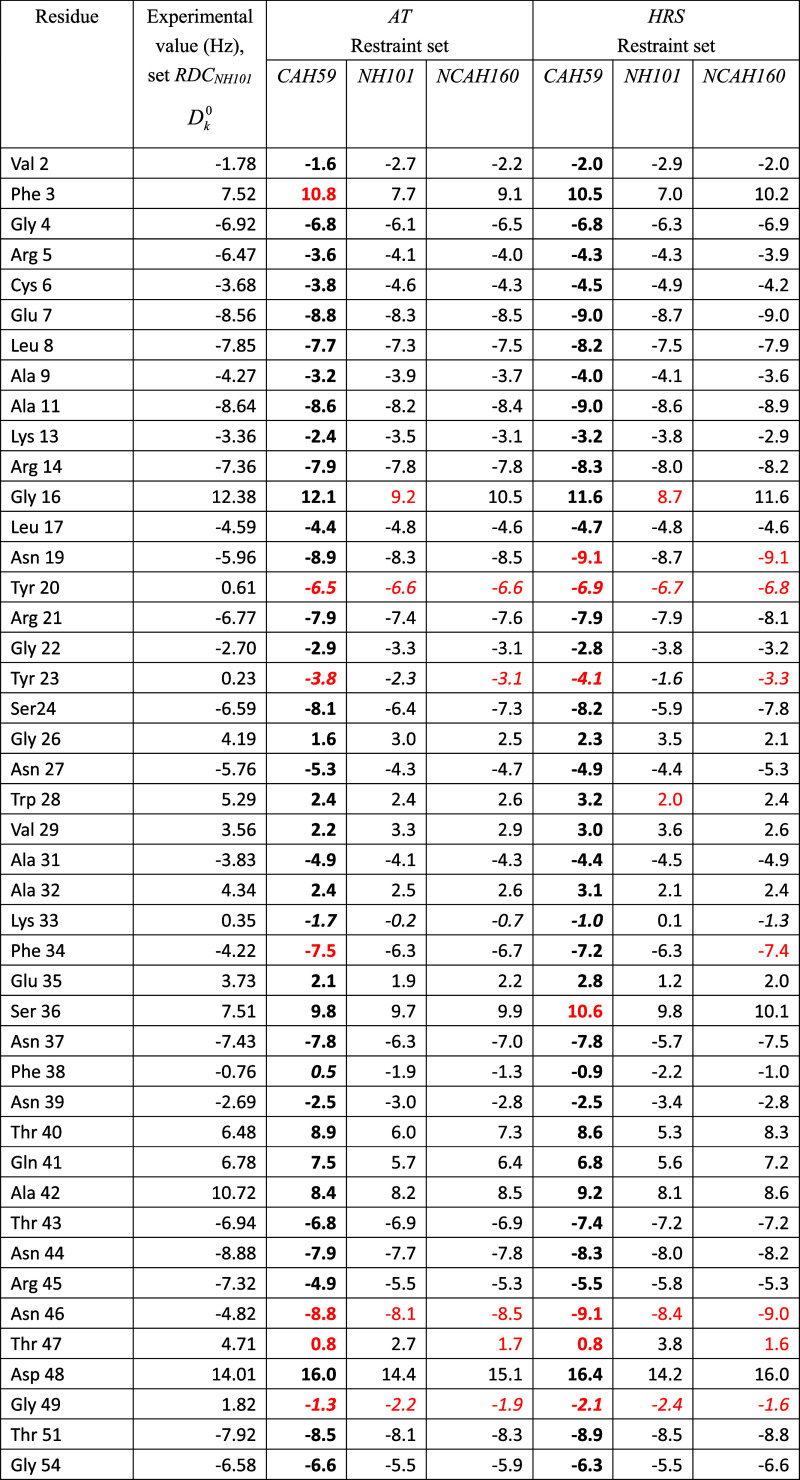

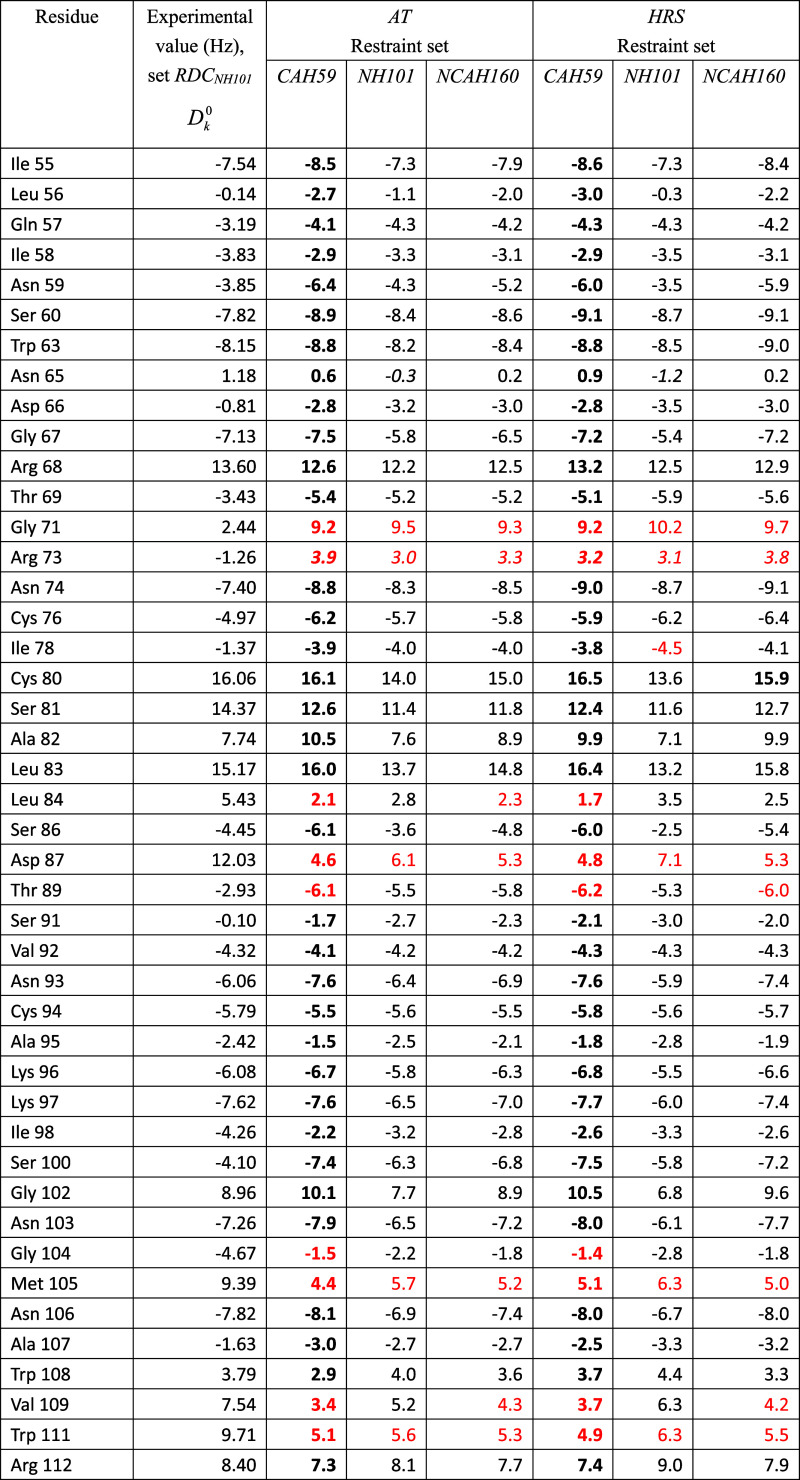

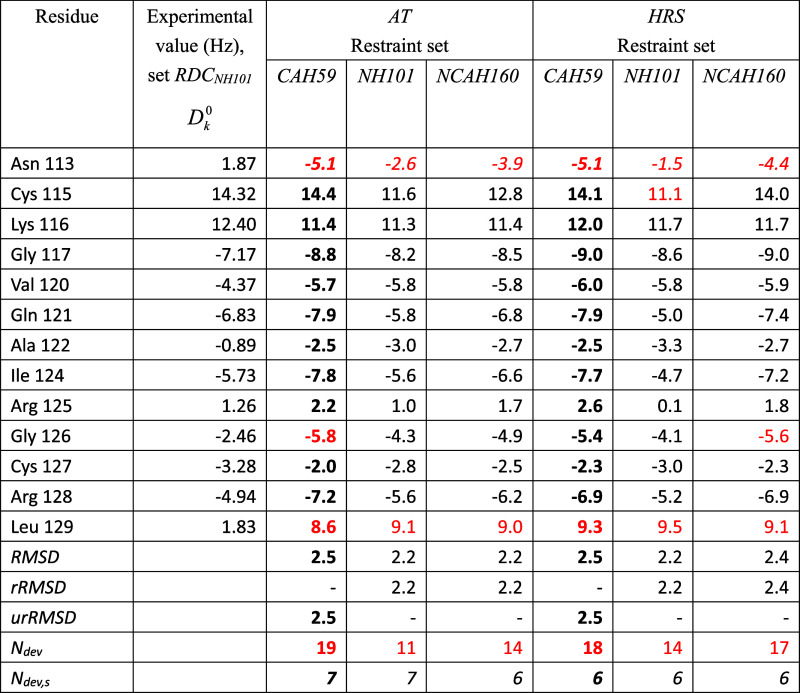
^15^N–^1^H^N^ RDC Values (101) in Hz for HEWL, as Obtained From NMR Measurements at 308 K and pH = 3.8, Table 4.1 of ref 16, and as Calculated Using Three Different Sets of RDC Restraints (RDC_*CAH59*_, RDC_*NH101*_, RDC_*NCAH160*_) for the X-ray structure 4LZT by Applying the Alignment-Tensor Method (AT: τ_D_^RDC^ = 0, τ_AT_^RDC^ = 0)^13^ or the HRS (*K* ^RDC,*msy*^ = 0) Method,^14^ *K* ^RDC*,mfv*^ = 100 kJ mol^–1^ Hz^–2^, τ_θ_^RDC,*mfv*^ = 10 ns, in 30 ns of SD Simulation of the Magnetic-Field Vector^a^

a The RDC restraint set RDC_*CAH59*_ contains the RDC values *D*_*k*_^0^ given in the fourth column of Table 1. The RDC restraint set RDC_*NH101*_ contains the RDC values *D*_*k*_^0^ given in the second column. The RDC restraint set RDC_*NCAH160*_ is obtained by combining the sets of RDC restraints RDC_*CAH59*_ and RDC_*NH101*_. The values for the RDCs that are *not* part of the (sub)­set of RDC restraints applied are in bold. RMSD: Root-mean-square difference (RMSD) between calculated *D*_*k*_ and *D*_*k*_^0^ RDC values calculated over all, *mfv*-restrained and unrestrained, RDCs. rRMSD: RMSD values calculated over the particular (sub)­set of *mfv*-restrained RDCs. urRMSD: RMS values calculated over the unrestrained RDCs. Deviations of RDC values *D*_*k*_1_ *k*_2__ (AT) or averaged ⟨*D*_*k*_1_ *k*_2__⟩_*t*_^*mfv*^ (HRS) from the *D*_*k*_^0^ values larger than 3 Hz are in red. *N*_dev_: Number of such deviations. *N*_dev,s_: Number of RDCs for which the calculated *D*_*k*_ and the *D*_*k*_^0^ values have a different sign. These RDC values are in italics.

**Table 3:**
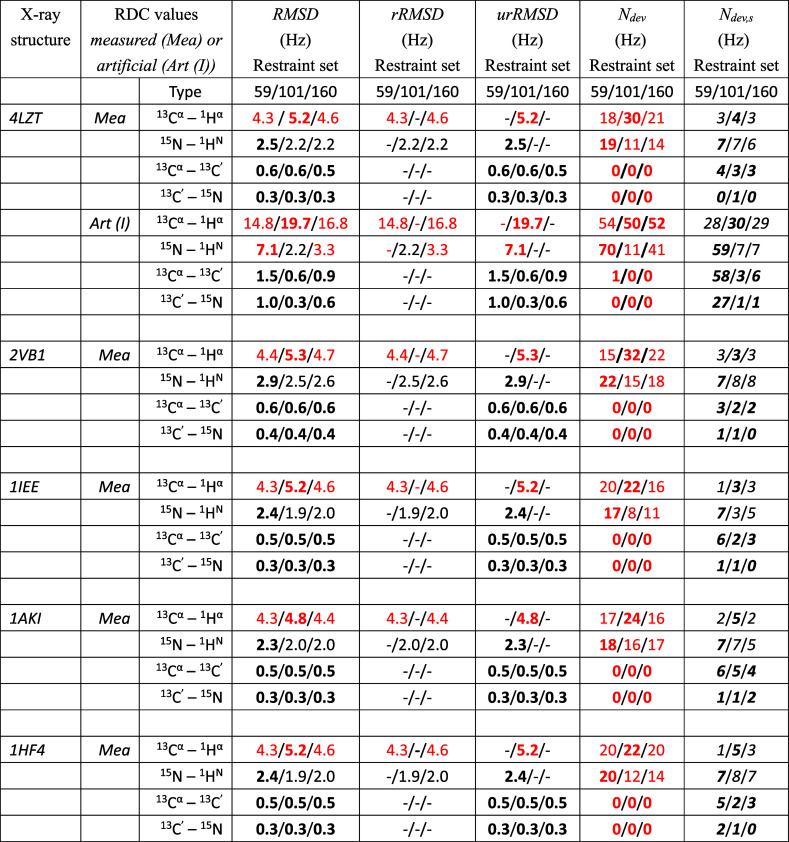
Summary of the Differences between Calculated (**AT** Method) and Measured (Mea) or Artificial (Art) RDC Values Serving as Target RDC Values, for Four Types of RDCs (59 ^13^C^α^–^1^H^α^ Values, 101 ^15^N–^1^H^N^ Values, 97 ^13^C–^13^C′ Values, and 45 ^13^C′–^15^N Values), for Five Different X-ray Structures (4LZT, 2VB1, 1IEE, 1AKI, and 1HF4) Using Three Different Sets of Measured (Mea) RDC Restraints (RDC_*CAH59*_, RDC_*NH101*_, and RDC_*NCAH160*_) or Artificial (Art) RDC Restraints (RDC_*CAH59I*_, RDC_*NH101I*_, and RDC_*NCAH160I*_)­^a^

a The values are taken from Tables 1, 2, 9, 10, S1–S18, S21, and S22. The values for the RDCs that are not part of the (sub)­set of RDC restraints applied are in bold. RMSD: Root-mean-square difference (RMSD) between calculated *D*_*k*_ and *D*_*k*_^0^ RDC values (in Hz) calculated over all, restrained and unrestrained, RDCs. rRMSD: RMSD values calculated over the particular (sub)­set of restrained RDCs. urRMSD: RMSD values calculated over the unrestrained RDCs. Deviations of calculated RDC values *D*_*k*_1_ *k*_2__ from the *D*_*k*_^0^ *D*_*k*_^0^ values larger than 3 Hz are in red. *N*_dev_: Number of such deviations. *N*_dev,s_: Number of RDCs for which the calculated *D*_*k*_ and the *D*_*k*_^0^ values have a different sign. These RDC values are in italics.

**Table 4:**
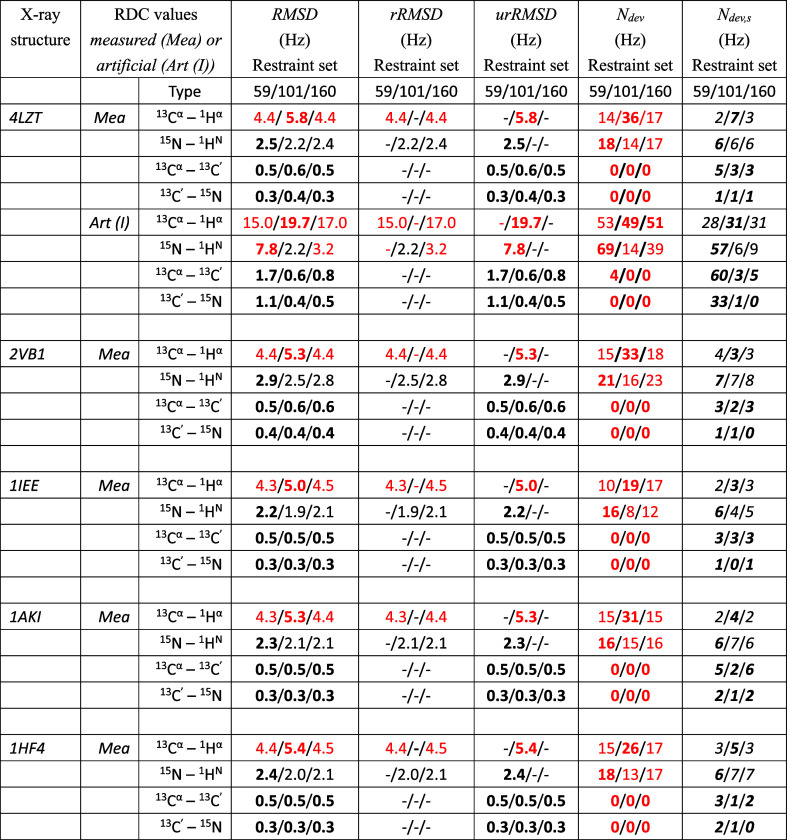
Summary of the Differences between Calculated (HRS Method) and Measured (Mea) or Artificial (Art) RDC Values Serving as Target RDC Values, for Four Types of RDCs (59 ^13^C^α^–^1^H^α^ Values, 101 ^15^N–^1^H^N^ Values, 97 ^13^C–^13^C′ Values, and 45 ^13^C′–^15^N Values), for Five Different X-ray Structures (4LZT, 2VB1, 1IEE, 1AKI, and 1HF4) Using Three Different Sets of Measured (Mea) RDC Restraints (RDC_*CAH59*_, RDC_*NH101*_, and RDC_*NCAH160*_) or Artificial (Art) RDC Restraints (RDC_*CAH59I*_, RDC_*NH101I*_, and RDC_*NCAH160I*_)­^a^

a The values are taken from Tables 1, 2, 9, 10, S1–S18, S21, and S22. The values for the RDCs that are not part of the (sub)­set of RDC restraints applied, are in bold. RMSD: root-mean-square difference (RMSD) between calculated *D*_*k*_ and *D*_*k*_^0^ RDC values (in Hz) calculated over all, *mfv*-restrained, and unrestrained, RDCs. rRMSD: RMSD values calculated over the particular (sub)­set of *mfv*-restrained RDCs. urRMSD: RMSD values calculated over the unrestrained RDCs. Deviations of averaged ⟨*D*_*k*_1_ *k*_2__⟩_*t*_^*mfv*^RDC values from the *D*_*k*_^0^ values larger than 3 Hz are in red. *N*_dev_: Number of such deviations. *N*_dev,s_: Number of RDCs for which the calculated *D*_*k*_ and the *D*_*k*_^0^ values have a different sign. These RDC values are in italics.

These four types or sets of RDC values show different sizes, which are roughly proportional to their *D*_*k*_^*c*^ values, eq 3. The sizes of the RDC values of the sets RDC_*CAH59*_ and RDC_*NH101*_ are an order of magnitude larger than those of the sets RDC_*CAC97*_ and RDC_*CN45*_. These sets can be combined into different sets of *N*_RDC_ target *D*_*k*_^0^ values for RDC restraining: For example, one set, RDC_*NCAH160*_, containing the 160 ^13^C^α^–^1^H^α^ and ^15^N–^1^H^N^ RDCs (Tables 1 and 2).

The differences in RDC values for the same residue between two sets of measured ^13^C^α^–^1^H^α^ RDCs, see Table 1, range from 0.1 to 5.4 Hz, with an RMSD of 2.3 Hz for the 18 RDC values. This suggests an experimental accuracy of a few Hz for these RDC values. For the other three types of RDCs, the sets of ^15^N–^1^H^N^, ^13^C^α^–^13^C′ and ^13^C′–^15^N RDC values, there are no two independent sets of measurements available. In view of this, the flat-bottom parameter of the restraining function (HRS method) was set to Δ*D* ^fb^ = 2 Hz, resulting in a width of 4 Hz of the flat bottom of the restraining potential-energy function, eqs 8a and 8b. This value is much smaller than the ranges of the RDC values of sets RDC_*CAH59*_ and RDC_*NH101*_, whereas it is large compared to the small ranges of a few Hz measured for the RDCs of sets RDC_*CAC97*_ and RDC_*CN45*_. The variation of the RDC values between the different residues of sets RDC_*CAC97*_ and RDC_*CN45*_ is smaller than 2 Hz. Since RDC values are the result of averaging over a slightly nonuniform rotational distribution, which reduces over 3 orders of magnitude positive and negative values of kHz size of a dipolar coupling (DC) to RDC values of Hz size, the smaller the RDC values are, the longer the sampling of this distribution will be required when calculating RDC values. For a two-atomic molecule SD simulated in vacuo, it already required microseconds to obtain a 1000-fold reduction in size.^10^ For a protein in aqueous solution, the sampling would require sampling times orders of magnitude longer than the rotational correlation time to obtain sufficient accuracy. So only sets RDC_*CAH59*_ and RDC_*NH101*_ were used for RDC restraining (HRS method) or as target RDC values when minimizing the difference between calculated and target RDC values (AT method).

### 4.4 Artificially Defined RDC Values Serving as Target RDC Values

As a test of the sensitivity of calculated RDC values to the spatial distribution of RDCs over the molecule and their compatibility with a given structure, a set of 59 artificial target RDC values was obtained by inverting the sequence of the 59 CA-HA target values along the backbone of HEWL, see Table 9. The corresponding set of RDC restraints is called RDC_*CAH59I*_. The combination of this set (RDC_*CAH59I*_) with the (measured) set of RDC restraints RDC_*NH101*_ is denoted as RDC_*NCAH160I*_.

**Table 5:** Root-Mean-Square Differences (RMSD­(*S*1 – *S*2) in Hz) between RDC Values Calculated Using the AT Method and Using the HRS Method, for Three Different Sets of Types of RDCs (*CAH59* (Mea), *CAH59I* (Art), and *NH101* (Mea)), for Three Different Measured (Mea) Target RDC Restraint Sets (*CAH59*, *NH101*, and *NCAH160*), for Two Different Artificial (Art) Target RDC Restraint Sets (*CAH59I* and *NCAH160I*) and Five X-ray Structures (4LZT, 2VB1, 1IEE, 1AKI, and 1HF4)­^a^

RDC	RMSD(*S*1 – *S*2)						
type set	restraint set	4LZT (Mea)	2VB1 (Mea)	1IEE (Mea)	1AKI (Mea)	1HF4 (Mea)	4LZT (Art)
*CAH59(I)*	*CAH59(I)*	0.72	0.85	0.59	0.82	1.00	2.59
*CAH59(I)*	*NH101*	1.14	0.15	0.54	1.05	0.53	1.14
*CAH59(I)*	*NCAH160(I)*	1.02	1.54	0.86	0.96	0.76	0.62
*NH101*	*CAH59(I)*	0.42	0.45	0.35	0.48	0.59	1.26
*NH101*	*NH101*	0.50	0.08	0.28	0.55	0.24	0.50
*NH101*	*NCAH160(I)*	0.48	0.73	0.41	0.47	0.40	0.32

a AT: alignment-tensor method (**τ**_D_^RDC^= 0, **τ**_AT_^RDC^= 0). HRS: magnetic-field rotation method (*K* ^RDC*,msy*^ = 0, *K* ^RDC*,mfv*^ = 100 kJ mol^–1^ Hz^–2^, **τ**_θ_^RDC,*mfv*^ = 10 ns, *t* ^*mfv*^ = 30 ns). RMSD values larger than 3 Hz are in (red) italics.

**Table 6:**
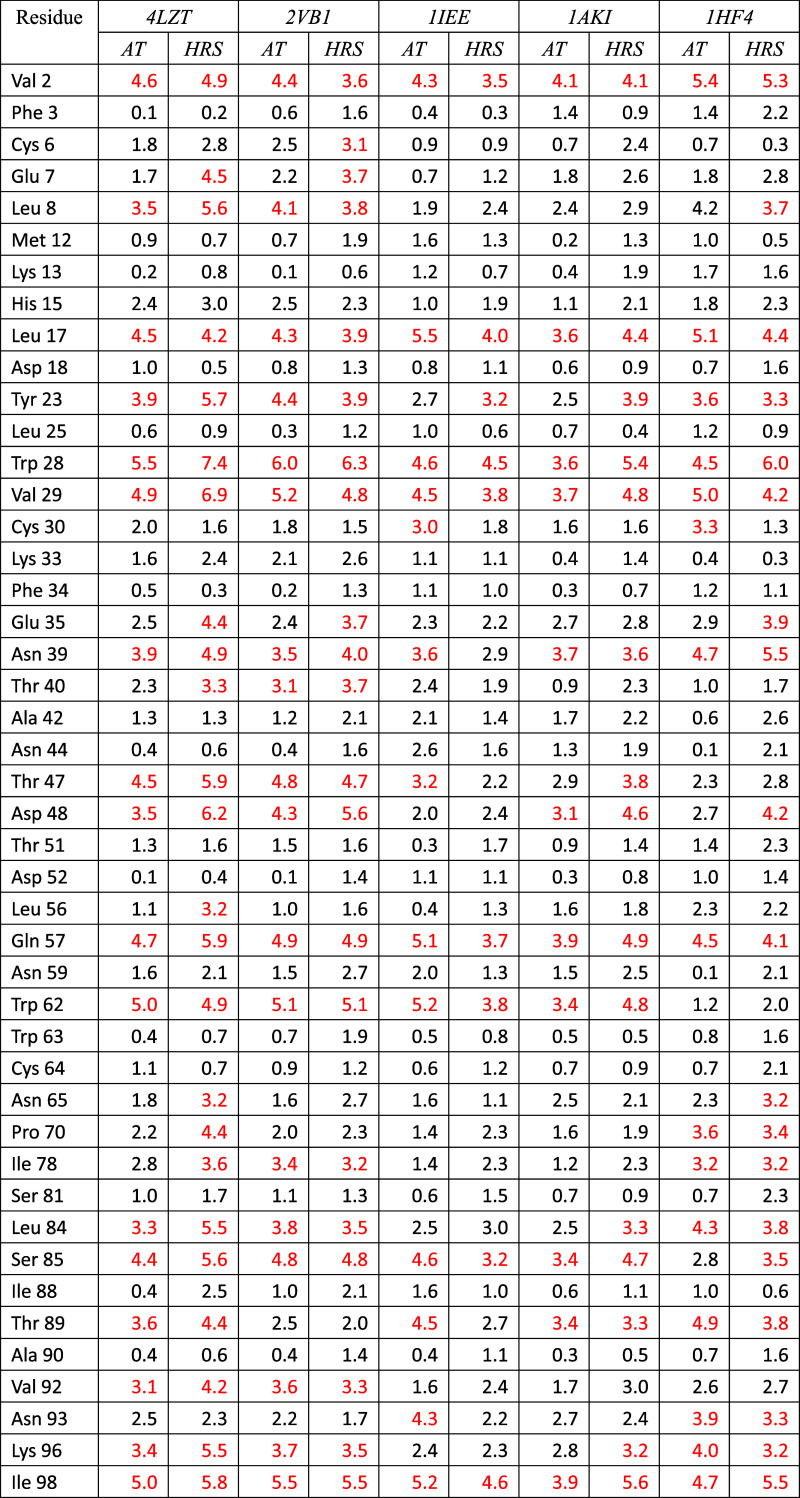

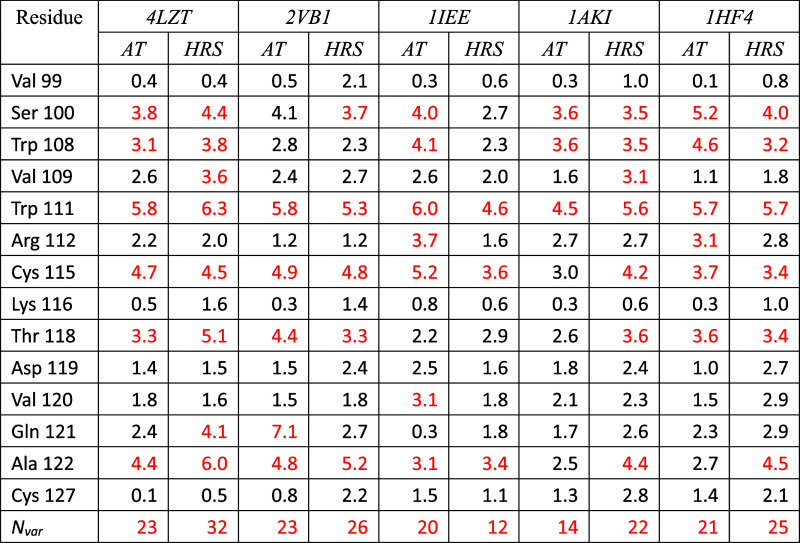
Largest Variation in ^13^C^α^–^1^H^α^ RDC Values (59) in Hz for HEWL Between Three Different Sets of RDC Restraints (CAH59, NH101, and NCAH160) Using the Alignment-Tensor (AT) Method or the Magnetic-field Rotation (HRS) Method, for Five X-ray Structures, *4LZT*, *2VB1*, *1IEE*, *1AKI*, and *1HF4*, Obtained from the Data in Tables 1, S3, S7, S11, and S15, Respectively^a^

a Variations larger than 3 Hz are in red. *N*_var_: Number of such variations.

**Table 7:**
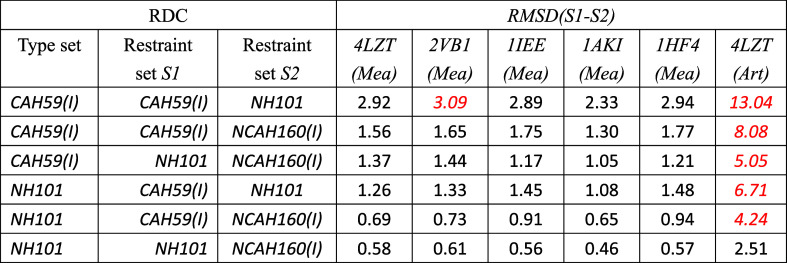
Root-Mean-Square Differences (RMSD­(*S*1*–S*2), in Hz) from Using the AT Method between RDC Values Calculated for Three Different Sets of Types of RDCs (*CAH59* (Mea), *CAH59I* (Art), and *NH101* (Mea)), Using Three (*CAH59*, *NH101*, and *NCAH160*) Different Measured (Mea) Target RDC Restraint Sets or Two Different Artificial (Art) Target RDC Restraint Sets (*CAH59I* and *NCAH160I*), for Five X-ray Structures (4LZT, 2VB1, 1IEE, 1AKI, and 1HF4)­^a^

a AT: alignment-tensor method (**τ**_D_^RDC^= 0, **τ**_AT_^RDC^= 0). RMSD values larger than 3 Hz are in (red) italics.

**Table 8:**
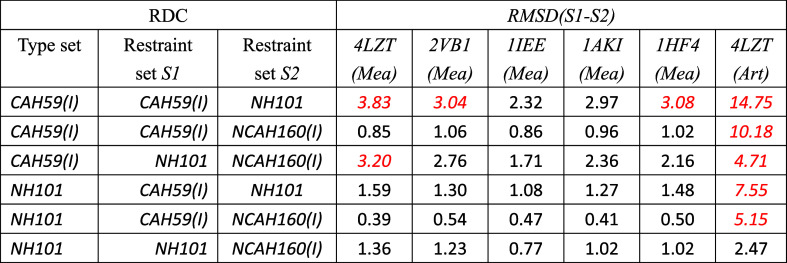
Root-Mean-Square Differences (RMSD­(*S*1 – *S*2), in Hz) from Using the HRS Method between RDC Values Calculated for Three Different Sets of Types of RDCs (*CAH59* (Mea), *CAH59I* (Art), and *NH101* (Mea)), Using Three (*CAH59*, *NH101*, and *NCAH160*) Different Measured (Mea) Target RDC Restraint Sets or Two Different Artificial (Art) Target RDC Restraint Sets (*CAH59I* and *NCAH160I*), for Five X-ray Structures (4LZT, 2VB1, 1IEE, 1AKI, and 1HF4)­^a^

a HRS: magnetic-field rotation method (*K* ^RDC*,msy*^ = 0, *K* ^RDC*,mfv*^ = 100 kJ mol^–1^ Hz^–2^, **τ**_θ_^RDC,*mfv*^ = 10 ns, *t* ^*mfv*^ = 30 ns). RMSD values larger than 3 Hz are in (red) italics.

**Table 9:**
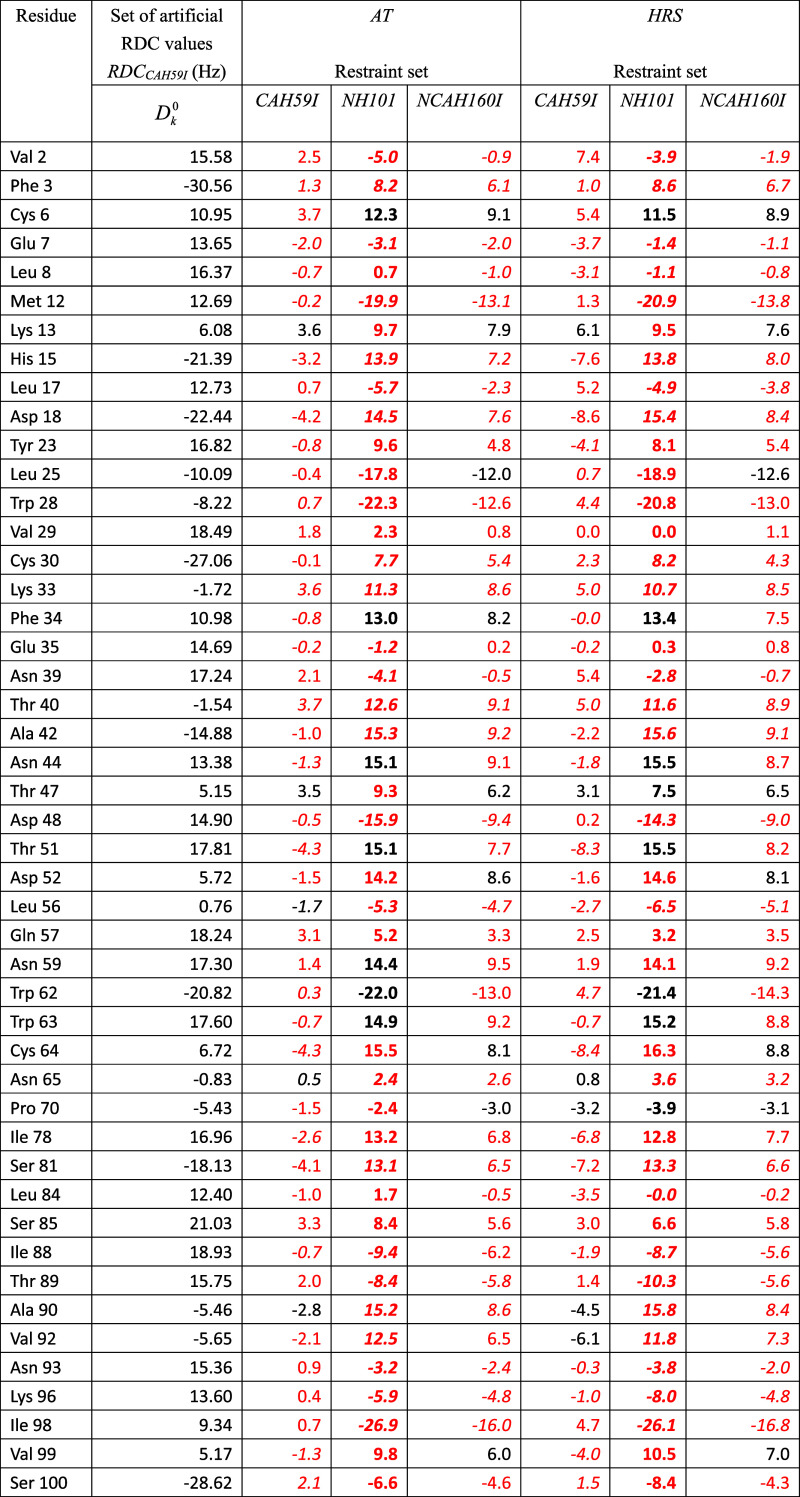

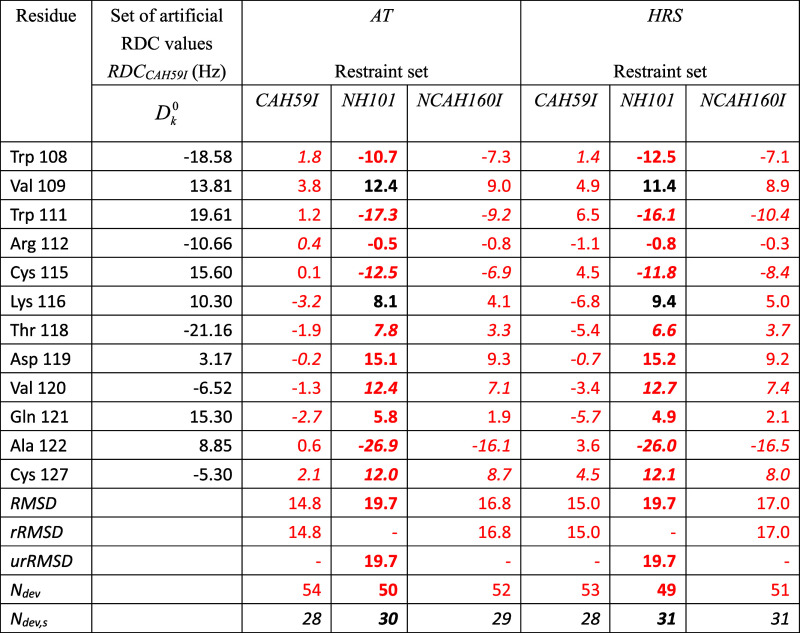
Artificial ^13^C^α^–^1^H^α^ RDC Values (59), set RDC_*CAH59I*_, in Hz for HEWL Calculated Using Three Different Sets of RDC Restraints (RDC_*CAH59I*_, RDC_*NH101*_, and RDC_*NCAH160I*_) for the X-ray structure 4LZT by Applying the Alignment-Tensor Method (AT: **τ**_D_^RDC^ = 0, **τ**_AT_^RDC^ = 0)^13^ or the HRS (*K* ^RDC*,msy*^ = 0) Method,^14^ *K* ^RDC*,mfv*^ = 100 kJ mol^–1^ Hz^–2^, **τ**_θ_^RDC,*mfv*^ = 10 ns, in *t* ^*mfv*^ = 30 ns SD Simulations of the Magnetic-field Vector^a^

a The (RDC restraint) set RDC_*CAH59I*_ is obtained by inverting the sequence of RDC values *D*_*k*_^0^ as given in the fourth column of Table 1. The RDC restraint set RDC_*NH101*_ contains the RDC values *D*_*k*_^0^ given in the second column of Table 2. The RDC restraint set RDC_*NCAH160I*_ is obtained by combining the sets of RDC restraints RDC_*CAH59I*_ and RDC_*NH101*_. The values for the RDCs that are *not* part of the (sub)­set of RDC restraints applied, are in bold. RMSD: Root-mean-square difference (RMSD) between calculated *D*_*k*_ and *D*_*k*_^0^ RDC values calculated over all, restrained and unrestrained, RDCs. rRMSD: RMSD values calculated over the particular (sub)­set of restrained RDCs. urRMSD: RMSD values calculated over the unrestrained RDCs. Deviations of calculated RDC values *D*_*k*_1_ *k*_2__ (AT) or averaged ⟨*D*_*k*_1_ *k*_2__⟩_*t*_^*mfv*^ (HRS) from the *D*_*k*_^0^ values larger than 3 Hz are in red. *N*_dev_: Number of such deviations. *N*_dev,s_: Number of RDCs for which the calculated *D*_*k*_ and the *D*_*k*_^0^ values have a different sign. These RDC values are in italics.

### 4.5 Analysis of Magnetic-Field Orientation Distributions and RDC Values

When applying the HRS method, the value of ${\overset{®}{D_{k_{1}k_{2}}\left(t\right)\;}}^{t}$ can be shown as a function of time in order to analyze its convergence. The nonuniformity or anisotropy of the rotational distribution of the angle θ_*k*_1_ *k*_2_,*h*_1_ *h*_2__ or in short-hand notation θ_*k*_1_ *k*_2_,*H*_ between the RDC-vector for *D*_*k*_1_ *k*_2__ and the vector *r⃗*_*h*_1_ *h*_2__ representing the direction of the magnetic field *H⃗* can be calculated from the magnetic-field vector SD trajectories. No magic-angle peaks should appear in the distribution of angle θ_*k*_1_ *k*_2_,*H*_ between the RDC-vector for *D*_*k*_1_ *k*_2__ and the magnetic-field direction *H⃗*. They would indicate improper rotational sampling. Since the protein is kept rigid, and only single X-ray structures are used, it suffices to calculate the rotational distribution for three roughly orthogonal vectors *r⃗*_*k*_1_ *k*_2__, connecting atoms *k*_1_ and *k*_2_ in the protein of the angle θ_*k*_1_ *k*_2_,*h*_1_ *h*_2__ between vector *r⃗*_*k*_1_ *k*_2__ and vector *r⃗*_*h*_1_ *h*_2__ representing the direction of the magnetic field *H⃗* from the magnetic-field vector SD trajectories. Here, we used vectors *r⃗*_*k*_1_ *k*_2__ connecting two C^α^ atoms in three helices: residues 4–15, residues 24–36, and residues 89–99.

In the present case of RDC-restraining, using a flat bottom of *D*_*k*_^0^ ± 2 Hz in the restraining potential-energy term, deviations |⟨*D*_*k*_⟩ – *D*_*k*_^0^| ≤ 3 Hz are considered insignificant.

### 4.6 Choice of Parameter Values for RDC-Restraining of the Magnetic-Field Vector in the HRS Method

The parameter Δ*D* ^h^ restricting the range of the harmonic part of the restraining function, eqs 8a and 8b, beyond which this function becomes linear, was set equal to 1 Hz. An HRS calculation of RDC values for a single protein structure requires a choice of the number *N*_*mfv*_ of SD time steps of the magnetic-field vector simulations, a value of the force constant *K* ^RDC*,mfv*^ restraining the magnetic-field vector toward the target, experimentally derived, RDC values, and a value of the memory relaxation time τ_θ_^RDC,*mfv*^, which controls the rotational averaging of the calculated RDC values. The SD simulation of the rotation of the magnetic-field vector should be sufficiently long to obtain converged RDC values. The 30 ns of rotational sampling of the magnetic-field vector seems enough to obtain converged RDC values (data not shown). The ratio *K* ^RDC*,mfv*^/τ_θ_^RDC,*mfv*^ determines the size of the RDC-restraining force. If this force is too large, magic-angle peaks will appear in the rotational distribution of the magnetic-field vector.^14,15^ If this force is too small, the rotational distribution of the magnetic-field vector will not be biased toward nonuniformity. As in refs 14,15 various combinations of *K* ^RDC*,mfv*^ and τ_θ_^RDC,*mfv*^ values were investigated (data not shown). The combination of *K* ^RDC*,mfv*^ = 100 kJ mol^–1^ Hz^–2^ and τ_θ_^RDC,*mfv*^ = 10 ns appeared to yield low differences between calculated and target RDC values without generating magic-angle peaks, see Figure 2. So these parameter values were used when further analyzing the data. The generated distributions of the angle θ_*k*_1_ *k*_2_,*H*_ between some vectors in the protein and the direction of the magnetic field *H* are rather noisy. The nonuniformity of the distribution required to obtain nonzero RDC values appears to be rather small.

**Figure 2:**
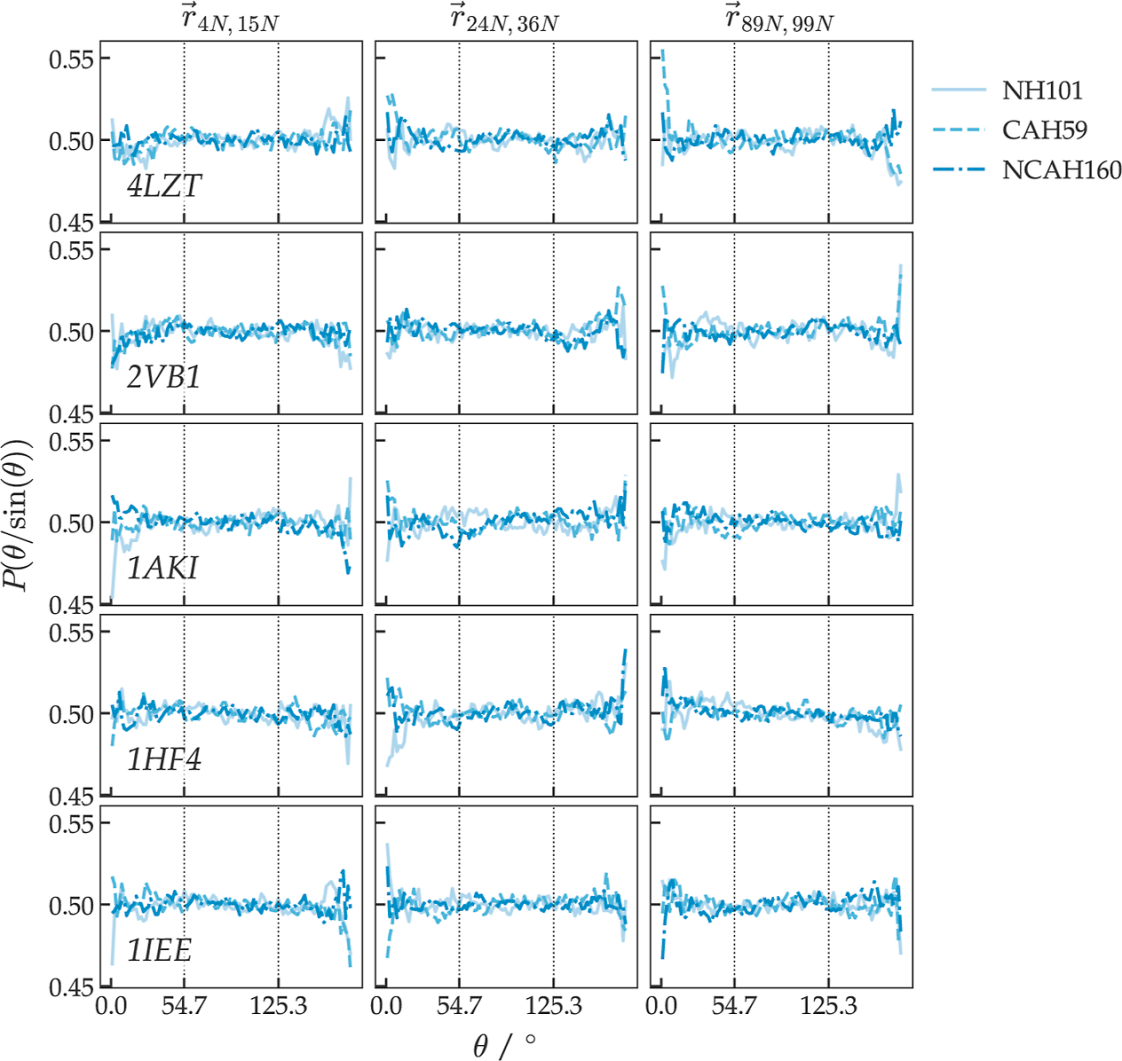
Distribution of θ_*k*_1_ *k*_2_,*H*_/sin­(θ_*k*_1_ *k*_2_,*H*_) between three approximately orthogonal vectors *r⃗*_*k*_1_ *k*_2__ connecting the C^α^ atoms of three helices, residues 4–15, residues 24–36, and residues 89–99, and the magnetic-field direction *H⃗* from three *mfv* SD simulations of 5000 time steps of 2 fs (*t* ^*mfv*^ = 10 ps) with *N*_*mfv*_ = 1000 resulting in 30 ns of rotational sampling. The HRS *mfv* parameter values were *K* ^RDC*,mfv*^ = 100 kJ mol^–1^ Hz^–2^ and **τ**_θ_^RDC,*mfv*^ = 10 ns. Three sets of RDC restraints were applied to the magnetic-field vector, RDC_*CAH59*_ (dashed lines), RDC_*NH101*_ (solid lines), and RDC_*NCAH160*_ (dot-dashed lines). (rows) Distributions for the five X-ray structures 4LZT, 2VB1, 1IEE, 1AKI, and 1HF4. (columns) The distributions for the three vectors *r⃗*_*k*_1_ *k*_2__.

The number *N*_RDC_ of RDCs used in the restraining was varied (*N*_RDC_ = 160, 101, and 59) in order to analyze the dependence of the obtained RDC values upon the size and composition (160: N–H and CA-H; 101: N–H; and 59: CA-H) of the set of backbone RDC target values used in the restraining.

### 4.7 Analysis of Structures

Atom-positional root-mean-square differences in RMSD between the X-ray structures were calculated after superimposing the backbone atoms (N, CA, and C) of residues 3–126.

The secondary structure assignment was done with the program DSSP, based on the Kabsch–Sander rules.^32^

Hydrogen bonds were identified according to a geometric criterion: a hydrogen bond was assumed to exist if the hydrogen-acceptor distance was less than 0.25 nm and the donor-hydrogen-acceptor angle was larger than 135°.

Backbone 3_10_-helical and α-helical hydrogen bonds and secondary-structure assignments are shown in Table S20.

## 5 Results

The RDC values calculated for the four types of experimentally derived RDCs for the backbone of the protein HEWL, 59 for CA-HA, 101 for N–H, 97 for CA-C′, and 45 for C′–N, are shown in Tables 1, 2, S1, and S2 for the X-ray structure 4LZT and in Tables S3–S18 for the X-ray structures 2VB1, 1IEE, 1AKI, and 1HF4.

The size of an RDC value depends on the product of the two gyromagnetic ratios γ_1_ and γ_2_ involved, see eq 1. This product is largest for the CA-HA RDCs, about half as large for the N–H RDCs, again about a factor of 2 smaller for the CA-C′ RDCs, and in turn a factor of 2 smaller for the C′–N RDCs. This may explain why the measured 59 CA-HA RDC values cover the widest range of values, [−31,+21] Hz, the 101 N–H RDC values display less variation, [−9,+16] Hz, whereas the 97 CA-C′ and the 45 C′–N RDC values are very small with ranges of [−3,+2] and [−1,+2] Hz, respectively. The difference in size by about a factor of 10 corresponds to the difference in dipolar coupling values of about a factor of 2^3^. For the latter two sets of RDCs, RDC values were calculated, but these sets were not used as restraints.

### 5.1 Calculation of RDCs Using the AT and HRS Methods

Table 1 shows that for the 59 CA-HA RDCs, RDC-restraining does not bring the calculated values close to the target ones. Using the AT method, the RMSD­(*D* – *D* ^0^) values are 4.3 Hz when using the restraint set RDC_*CAH59*_, 5.2 Hz using the restraint set RDC_*NH101*_, and 4.6 Hz using the combined restraint set RDC_*NCAH160*_. The number of deviations larger than 3 Hz between calculated *D*_*k*_ values and target *D*_*k*_^0^ values, *N*_dev_, is 18 (out of 59) when using the restraint set RDC_*CAH59*_, 30 when using the restraint set RDC_*NH101*_, and 21 when using the combined restraint set RDC_*NCAH160*_. The signs of the RDCs are well reproduced, and only *N*_dev,s_ = 3 or 4 out of 59 appear to be different (5 or 7%). This is not surprising because over its range [0°,180°], the Legendre function *P*_*k*_(θ), eq 5, see Figure 1 of ref 10, changes sign at the two magic angles, at 54° from positive to negative and at 125° from negative to positive. When the target value is small, and the θ angle is close to a magic angle value (small RDC values), a difference in sign may easily occur. The HRS method shows comparable results. For both methods (AT and HRS), the largest differences between calculated and target RDC values are observed for residues Asp 52 (>19 Hz), Asn 93 (>11 Hz), and Arg 112 (>11 Hz). This may be due to a structural incompatibility between the measured RDC value and the (X-ray) structure (4LZT) used in the calculation (see Section 5.5).

Table 2 shows that the 101 N–H RDCs are more easily reproduced with RMSD­(*D* – *D* ^0^) values ranging from 2.2 to 2.5 Hz when applying the three restraint sets using either the AT or the HRS method. Accordingly, the number of deviations between calculated *D*_*k*_ values and target *D*_*k*_^0^ values, *N*_dev_, is 11 to 19 (out of 101), smaller than the range 14–36 (out of 59) for the 59 CA-HA RDCs. The largest differences between calculated and target RDC values are observed for residues Tyr 20 (>7 Hz), Gly 71 (>6 Hz), Asp 87 (>5 Hz) and Leu 129 (>6 Hz). Considering the number of RDC values, the signs of the RDCs (*N*_dev*,*s_) are equally well reproduced; 6 to 7 out of 101 appear to be different (6 to 7%).

Table S1 shows, as expected in view of their relatively small DC values, that the 97 CA-C′ RDCs are well reproduced in the calculations, although these RDCs were not restrained. RMSD­(*D* – *D* ^0^) values are 0.5 or 0.6 Hz, *N*_dev_ = 0 and *N*_dev*,*s_ is 3 to 5 (3 or 5%).

Table S2 shows a similar picture for the (small) 45 C′–N RDCs, which were also not restrained in the calculations. RMSD­(*D* – *D* ^0^) values are 0.3 or 0.4 Hz, *N*_dev_ = 0 and *N*_dev*,*s_ is 1 or 0 (2 or 0%).

That a set of target RDC values cannot be closely reproduced in an AT or HRS calculation can have various causes. The measured set of target values may suffer from experimentally induced inaccuracies, which renders them inconsistent with protein structure, or, being measured for a configurational Boltzmann-weighted ensemble of protein structures in aqueous solution at nonzero temperature, they may be incompatible with a single (protein) structure. Summaries of the differences between calculated and measured RDC values serving as target RDC values are presented in Table 3 (AT method) and Table 4 (HRS method).

### 5.2 Comparison of RDCs Calculated Using the AT and HRS Methods

Considering the three sets of RDC restraints, RDC_*CAH59*_, RDC_*NH101*_, and RDC_*NCAH160*_, the CA-HA RDC target values are slightly worse reproduced applying the RDC_*CAH59*_ set of restraints when using the HRS method than when using the AT method (Table 1), with RMSD­(*D* – *D* ^0^) values of 4.4 Hz (HRS) compared to 4.3 Hz (AT). For the N–H RDC target values by applying the RDC_*NH101*_ set of restraints (Table 2), the RMSD­(*D* – *D* ^0^) values are 2.2 Hz for both methods. Table 5 shows that the RMSD­(*D*(AT) – *D*(HRS)) value for the CA-HA RDCs using the restraint set RDC_*CAH59*_ is 0.72 Hz (structure 4LZT (Mea), first line), and for the N–H RDCs using the restraint set RDC_*NH101*_, it is 0.50 Hz (structure 4LZT (Mea), fifth line). The largest difference between applying the AT and HRS methods (using the two restraint sets RDC_*CAH59*_ and RDC_*NH101*_), with an RMSD­(*D*(AT) – *D*(HRS)) value of 1.14 Hz is observed for the CA-HA RDCs applying the RDC_*NH101*_ restraints (structure 4LZT (Mea), second line). Considering all five X-ray structures, the largest differences between the AT and HRS methods are observed for the 2VB1 (Mea) X-ray structure, 1.54 Hz for the CA-HA RDCs using the RDC_*NCAH160*_ restraints, for the 4LZT (Mea) X-ray structure, 1.14 Hz for the CA-HA RDCs using the RDC_*NH101*_ restraints, for the 1AKI (Mea) X-ray structure, 1.05 Hz for the CA-HA RDCs using the RDC_*NH101*_ restraints, and for the 1HF4 (Mea) X-ray structure, 1.00 Hz for the CA-HA RDCs using the RDC_*CAH59*_ restraints. The 1IEE (Mea) X-ray structure shows the smallest RMSD­(*D*(AT) – *D*(HRS)) values, ranging from 0.3 to 0.6 Hz (see Section 5.5).

### 5.3 Influence of the Set, Size, and Types of RDC Restraints on the Calculated RDC Values

Comparing the RMSD values for sets of restrained (rRMSD) and unrestrained (urRMSD) RDCs in Tables 1 and 2, the RMSD­(*D* – *D* ^0^) values of the latter are larger than that of the former, as expected. These tables, and the corresponding ones for the four other X-ray structures (Tables S3, S4, S7, S8, S11, S12, S15, and S16), also allow a detailed comparison of RDCs calculated using the three different sets of restraints, RDC_*CAH59*_, RDC_*NH101*_, and RDC_*NCAH160*_. For the CA-HA RDCs (Tables 1, S3, S7, S11 and S15), the largest differences between the RDCs calculated using the three different sets of restraints are shown in Table 6. Considering all five X-ray structures, differences larger than 3 Hz are observed for 14 to 23 residues using the AT method and for 12 to 32 residues using the HRS method, with the largest difference being 7.1 Hz (Gln 121, 2VB1 structure) for the AT method and 7.4 Hz (Trp 28, 4LZT structure) for the HRS method. For the N–HN RDCs (Tables 2, S4, S8, S12, and S16), the largest differences between the RDCs calculated using the three different sets of restraints are shown in Table S19. The differences for these RDCs are much smaller. Considering all five X-ray structures, differences larger than 3 Hz are only observed for 0 to 4 residues for the AT method and for 0 to 6 residues for the HRS method, the largest difference being 3.1 Hz for the AT method and 3.6 Hz for the HRS method.

Tables 7 (AT) and 8 (HRS) allow a comparison of RMSD values between RDCs calculated using the three different sets of restraints, RMSD­(*D*(*CAH59*) – *D*(*NH101*)), RMSD­(*D*(*CAH59*) – *D*(*NCAH160*)), and RMSD­(*D*(*NH101*) – *D*(*NCAH160*)), for the five X-ray structures, for both methods (AT and HRS) and both sets of RDCs (CA-HA and N–H). In Table 7 (AT), the RMSD values for the five X-ray structures vary between 0.5 Hz (RMSD­(*D*(*NH101*) – *D*(*NCAH160*)) for the N–HN RDCs in X-ray structure 1AKI) and 3.1 Hz (RMSD­(*D*(*CAH59*) – *D*(*NH101*)) for the CA-HA RDCs in X-ray structure 2VB1). Only one RMSD value is the only one that is larger than 3 Hz. Using the HRS method (Table 8), the RMSD values vary between 0.4 Hz (RMSD­(*D*(*CAH59*) – *D*(*NCAH160*)) for the N–HN RDCs in X-ray structure 4LZT) and 3.8 Hz (RMSD­(*D*(*CAH59*) – *D*(*NH101*)) for the CA-HA RDCs in X-ray structure 4LZT). Four RMSD values are larger than 3 Hz, all for CA-HA RDCs, three (RMSD­(*D*(*CAH59*) – *D*(*NH101*)) values (4LZT, 2VB1, and 1HF4 structures) and one RMSD­(*D*(*NH101*) – *D*(*NCAH160*)) value (4LZT structure).

The application of different sets of RDC restraints illustrates that the calculated RDC values depend on the size and type of the set of RDC restraints. They also depend on the orientational variation of the RDC-vector directions within the structure of the molecule.

### 5.4 Compatibility of the Set, Size, and Types of RDC Target Values or Restraints and the Structure for Which RDC Values are Calculated

Although the application of the three different sets of RDC restraints, RDC_*CAH59*_, RDC_*NH101*_, and RDC_*NCAH160*_, leads to different values of calculated RDCs, the differences are, except for a few residues, not very large. The three different restraint sets contain only RDCs of two types, CA-HA and N–HN, involving backbone atoms along similar parts of the backbone. So the three restraint sets, the one involving the CA-HA RDCs (RDC_*CAH59*_), the one involving the N–HN RDCs (RDC_*NH101*_), and the combination of these (RDC_*NCAH160*_), are likely to contain similar information regarding the relative directions of these RDC vectors. In addition, since most of the backbone of HEWL in solution is rather stable, most of the measured backbone RDCs are likely to be compatible with the HEWL structures present in solution.

As a test of the sensitivity of calculated RDC values to the spatial distribution of RDCs over the molecule and their compatibility with a given structure, a set of 59 artificial target RDC values was obtained by inverting the sequence of the 59 CA-HA target values along the backbone of HEWL, see Tables 7–9. The corresponding set of RDC restraints is called RDC_*CAH59I*_. The combination of this set (RDC_*CAH59I*_) with the (measured) set of RDC restraints RDC_*NH101*_ is denoted as RDC_*NCAH160I*_. Restraining toward the set RDC_*CAH59I*_ of target values is not very successful, with RMSD values of 14.8 Hz (AT) and 15.0 Hz (HRS). As expected, restraining toward the set RDC_*NH101*_ of (measured) target values leads for the (artificial) CA-HA RDCs to even larger RMSD values, 19.7 Hz for both methods. Restraining toward the set RDC_*NCAH160I*_, the combination of artificial and measured RDC values, yields, also as expected, somewhat smaller RMSD values, 16.8 Hz (AT) and 17.0 Hz (HRS). The number of deviations larger than 3 Hz, 49 to 54 out of 59 RDCs, is large in all cases, while 28 to 31 calculated RDCs appear to have a sign different from the target RDC value (about 50%).

Table 10 shows the results for the set of 101 N–HN RDCs. Of course, using the (artificial) restraint set RDC_*CAH59I*_ the (measured) N–HN RDC values are not well reproduced, with RMSD values of 7.1 Hz (AT) and 7.8 Hz (HRS). Using the combined (artificial, measured) restraint set RDC_*NCAH160I*_ the (measured) N–HN RDC values are better reproduced, with RMSD values of 3.3 Hz (AT) and 3.2 Hz (HRS).

**Table 10:**
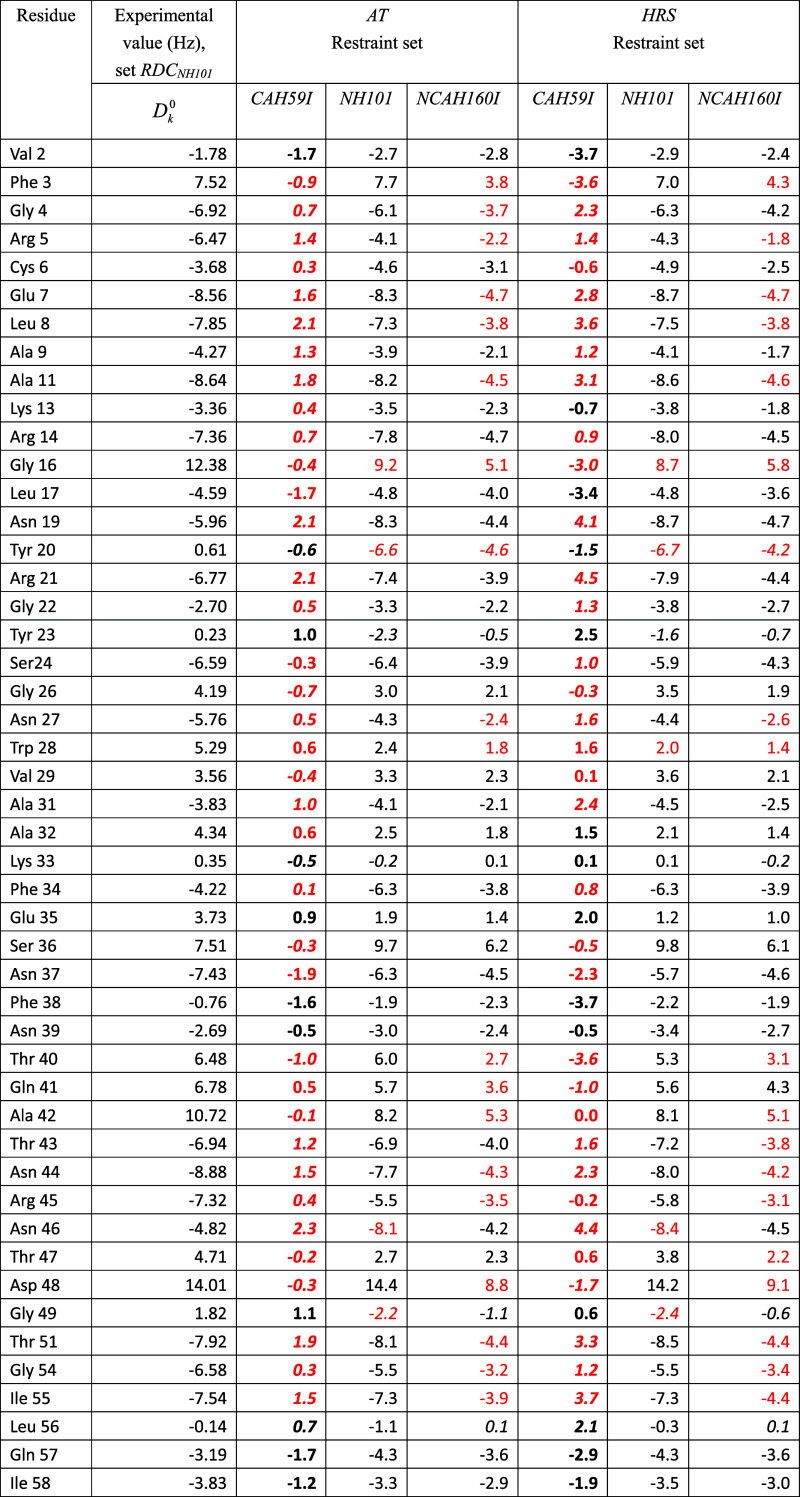

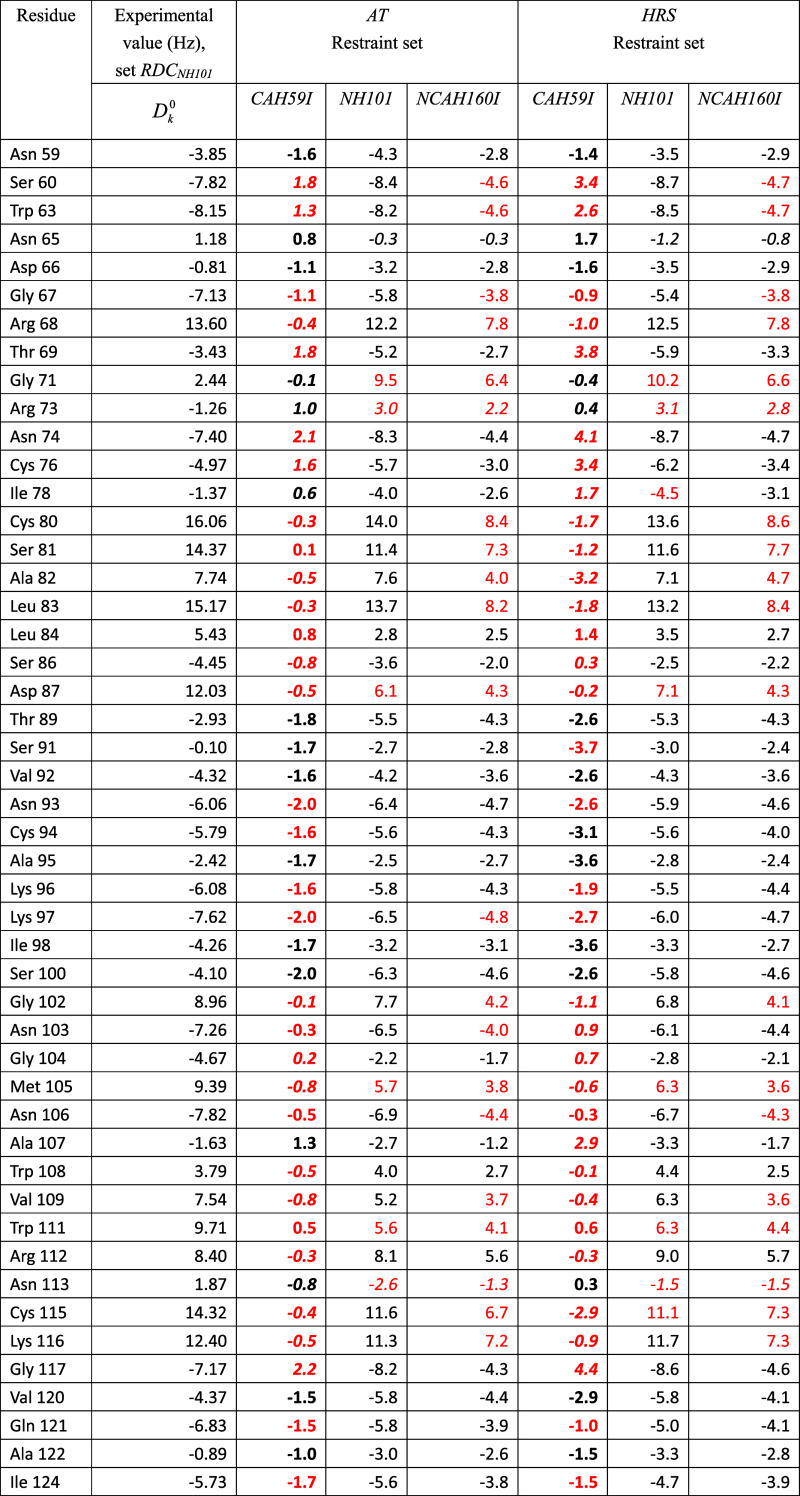

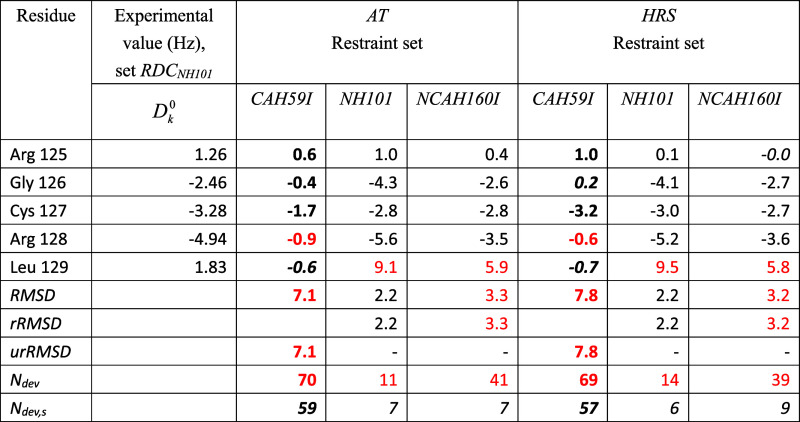
^15^N–^1^H^N^ RDC Values (101) in Hz for HEWL, as Obtained from NMR Measurements at 308 K and pH = 3.8, Table 4.1 of ref 16, and as Calculated Using Three Different Sets of RDC Restraints (RDC_*CAH59I*_, RDC_*NH101*_, and RDC_*NCAH160I*_) for the X-ray Structure 4LZT by the Applying the Alignment-Tensor Method (AT: **τ**_D_^RDC^ = 0, **τ**_AT_^RDC^ = 0)^13^ or the HRS (*K* ^RDC*,msy*^ = 0) Method,^14^ *K* ^RDC*,mfv*^ = 100 kJ mol^–1^ Hz^–2^, **τ**_θ_^RDC,*mfv*^ = 10 ns, in *t* ^*mfv*^ = 30 ns. SD Simulations of the Magnetic-Field Vector^a^

a The RDC restraint set RDC_*CAH59I*_ is obtained by inverting the sequence of RDC values *D*_*k*_^0^, as given in the fourth column of Table 1. The RDC restraint set RDC_*NH101*_ contains the RDC values *D*_*k*_^0^ given in the second column of Table 2. The RDC restraint set RDC_*NCAH160I*_ is obtained by combining the sets of RDC restraints RDC_*CAH59I*_ and RDC_*NH101*_. The values for the RDCs that are *not* part of the (sub)­set of RDC restraints applied are in bold. RMSD: Root-mean-square difference (RMSD) between calculated *D*_*k*_ and *D*_*k*_^0^ RDC values calculated over all, restrained and unrestrained, RDCs. rRMSD: RMSD values calculated over the particular (sub)­set of restrained RDCs. urRMSD: RMSD values calculated over the unrestrained RDCs. Deviations of RDC values *D*_*k*_1_ *k*_2__ (AT) or averaged ⟨*D*_*k*_1_ *k*_2__⟩_*t*_^*mfv*^ (HRS) from the *D*_*k*_^0^ values larger than 3 Hz are in red. *N*_dev_: Number of such deviations. *N*_dev,s_: Number of RDCs for which the calculated *D*_*k*_ and the *D*_*k*_^0^ values have a different sign. These RDC values are in italics.

Table S21 shows the results for the set of 97 CA-C′ RDCs. Using the (artificial) restraint set RDC_*CAH59I*_, the (measured) CA-C′ RDC values are, because they are small, almost all reproduced; only 1 or 4 RDC values deviate more than 3 Hz,. Yet, more than half of the calculated RDC values, 58 (AT) and 60 (HRS), appear to have a sign different from the measured values.

Table S22 shows the results for the set of 45 C′-N RDCs. Using the (artificial) restraint set RDC_*CAH59I*_, the (measured) C′–N RDC values are, because they are small, all reproduced within 3 Hz. Yet, more than half of the calculated RDC values, 27 (AT) and 33 (HRS), appear to have a sign different from the measured values.

Comparing the RMSD values for the RDC values calculated using the AT and HRS methods, Table 5 shows that the RMSD­(*D*(AT) – *D*(HRS)) value for the CA-HA RDCs using the restraint set RDC_*CAH59*_ is 0.72 Hz (structure 4LZT (Mea), first line), while using the restraint set RDC_*CAH59I*_, it is 2.59 Hz (structure 4LZT (Art), first line). The RMSD­(*D*(AT) – *D*(HRS)) value for the N–HN RDCs using the restraint set RDC_*CAH59*_ is 0.42 Hz (structure 4LZT (Mea), fourth line), while using the restraint set RDC_*CAH59I*_, it is 1.26 Hz (structure 4LZT (Art), fourth line). The two methods show somewhat larger differences in calculated RDC values in the case that the calculated and target RDC values are incompatible.

Tables 7 (AT) and 8 (HRS) allow a comparison of RMSD values between RDCs calculated using the three different sets (two artificial and one measured) of restraints, RMSD­(*D*(*CAH59I*) – *D*(*NH101*)), RMSD­(*D*(*CAH59I*) – *D*(*NCAH160I*)), and RMSD­(*D*(*NH101*) – *D*(*NCAH160I*)), for the 4LZT X-ray structure, for both methods (AT and HRS) and both sets of RDCs (artificial CA-HA and measured N–H). As expected, the largest differences are found for the artificial set (CAH59I) of target RDC values, with the RMSD­(*D*(*CAH59I*) – *D*(*NH101*)) values for the 4LZT X-ray structure being 13.04 Hz (AT, Table 7, first line, last column) and 14.75 Hz (HRS, Table 8, first line, last column). The RMSD­(*D*(*CAH59I*) – *D*(*NCAH160I*)) values are smaller with 8.08 HZ (AT, Table 7) and 10.18 Hz (HRS, Table 8). As expected, the RMSD­(*D*(*NH101*) – *D*(*NCAH160I*)) with values of 5.05 Hz (AT) and 4.71 Hz (HRS) are again smaller. For the set of measured (NH101) RDCs, the RMSD­(*D*(*CAH59I*) – *D*(*NH101*)) values are 6.71 (AT) and 7.55 Hz (HRS), while RMSD­(*D*(*CAH59I*) – *D*(*NCAH160I*)) values are 4.24 (AT) and 5.15 Hz (HRS). The only RMSD value smaller than 3 Hz is RMSD­(*D*(*NH101*) – *D*(*NCAH160I*)), with 2.51 Hz (AT) and 2.47 HZ (HRS).

The application of rather different sets of (artificial and measured) RDC restraints demonstrates that calculated RDC values depend on the size and type of the set of RDC restraints or RDC target values. This is due to the procedure to calculate RDC values, in which the difference between calculated and target RDC values is minimized (AT method) or the calculated RDC values are restrained toward the target ones (HRS method).

### 5.5 Comparison of the Five X-ray Structures and the RDC Values Calculated from Them

HEWL contains various secondary-structure elements: three β-strands, residues 41–45, 50–53 and 58–59, four α-helices, residues 4–14, 25–36, 88–99 and 108–113, and three 3_10_-helical helices, residues 19–22, 79–84 and 119–123, see Table S20 and Figure 3. This figure shows the 10 pairwise atom-positional distances (129 C^α^ and 129 N atoms) between the five X-ray structures as a function of residue number.

**Figure 3:**
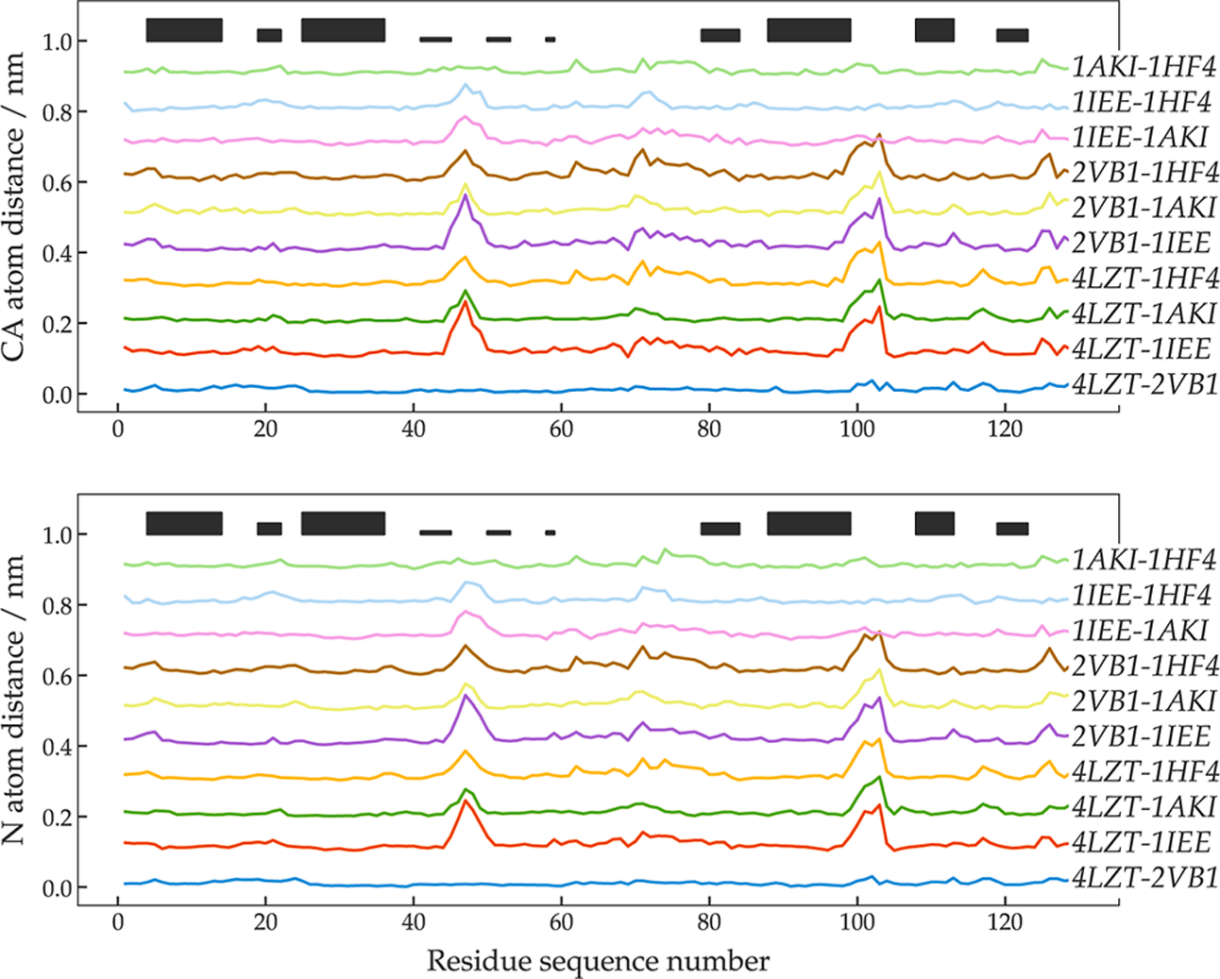
Ten pairwise atom-positional distances, 129 C^α^ (upper panel) and 129 N (lower panel) backbone atoms, between the five X-ray structures, 4LZT, 2VB1, 1IEE, 1AKI, and 1HF4, as a function of the residue number. The different X-ray structures were translationally and rotationally superimposed using the backbone atoms N, C^α^, and C′ of residues 3–126. The secondary-structure assignments are indicated by horizontal bars. Thick bars: α-helices. Thin bars: 3_10_-helices. Line bars: β-strands. Pair 4LZT–2VB1: dark blue; 4LZT–1IEE: red; 4LZT–1AKI: dark green; 4LZT–1HF4: dark yellow; 2VB1–1IEE: purple; 2VB1–1AKI: yellowish green; 2VB1–1HF4: brown; 1IEE–1AKI: magenta; 1IEE–1HF4: light blue; and 1AKI–1HF4: light green. The lines are consecutively shifted by 0.1 nm.

The largest structural differences are observed for residues around 48 and 103 and, to a lesser extent, for residues in the range 60–80 and at the C-terminus. The smallest structural differences are found between X-ray structures 4LZT and 2VB1 and between 1AKI and 1HF4.

Tables 11 (AT) and 12 (HRS) allow a comparison of RMSD values between RDCs calculated for the different X-ray structures. Generally, the RMSD values for the CA-HA RDCs (upper-right triangle) are larger than those for the N–HN RDCs (lower-left triangle), which is no surprise considering their slightly different sizes. The smallest differences are observed between the 4LZT and 2VB1 structures, both obtained for a triclinic cell but at different temperatures, with a largest difference of 1.30 Hz (AT) and 1.58 Hz (HRS) for the CA-HA RDCs using the RDC_*NH101*_ restraint set. For all other pairs of structures, the 108 (9 pairs, 2 types of RDCs, 3 restraint sets, 2 methods) RMSD values range from 1.37 (AT) and 1.45 Hz (HRS) for the N–HN RDCs using the RDC_*NH101*_ restraint set between the 4LZT and 1AKI structures to 2.88 Hz (AT) and 2.96 Hz (HRS) for the CA-HA RDCs using the RDC_*CAH59*_ restraint set between the 1IEE and 1AKI structures.

**Table 11:** Root-mean-square Differences (RMSD­(*X*1 – *X*2) in Hz) from Using the AT Method between RDC Values Calculated between 10 Pairs of X-ray (4LZT, 2VB1, 1IEE, 1AKI, and 1HF4) Structures, Using Three Different Sets of RDC Restraints (*CAH59*, *NH101*, and *NCAH160*, Separated by the Symbol “/”) for Two Different Sets of Types of RDCs (*CAH59* in the Upper-Right Triangle and *NH101* in the Lower-Left Triangle)^a^

	4LZT	2VB1	1IEE	1AKI	1HF4
4LZT		1.30/1.30/1.29	2.25/2.18/2.16	2.12/2.11/2.07	1.71/1.86/1.73
2VB1	0.97/0.81/0.87		2.62/2.60/2.54	2.24/2.29/2.21	1.99/2.24/2.06
1IEE	1.89/1.67/1.75	2.30/1.97/2.10		2.88/2.72/2.78	1.81/2.19/1.95
1AKI	1.61/1.37/1.46	2.02/1.70/1.82	1.76/1.47/1.57		2.19/2.25/2.18
1HF4	2.13/1.74/1.88	2.42/2.02/2.15	2.02/1.72/1.83	1.84/1.57/1.66	

a AT: alignment-tensor method (**τ**_D_^RDC^= 0, **τ**_AT_^RDC^= 0). RMSD values larger than 3 Hz are in (red) italics.

**Table 12:** Root-Mean-Square Differences (RMSD­(*X*1 – *X*2) in Hz) from Using the HRS Method between RDC Values Calculated between 10 Pairs of X-ray (4LZT, 2VB1, 1IEE, 1AKI, and 1HF4) Structures, Using Three Different Sets of RDC Restraints (*CAH59*, *NH101*, *NCAH160*, Separated by the Symbol “/”) for Two Different Sets of Types of RDCs (*CAH59* in the Upper-Right Triangle and *NH101* in the Lower-Left Triangle)^a^

	4LZT	2VB1	1IEE	1AKI	1HF4
4LZT		1.46/1.58/1.40	2.31/2.58/2.35	2.15/2.25/2.21	1.85/1.92/1.78
2VB1	1.01/0.97/1.00		2.69/2.50/2.86	2.37/2.16/2.46	2.13/2.07/2.26
1IEE	1.94/1.80/1.85	2.34/2.00/2.33		2.96/2.71/2.88	1.97/2.05/1.90
1AKI	1.68/1.45/1.66	2.11/1.80/2.16	1.79/1.61/1.65		2.28/2.21/2.17
1HF4	2.16/1.77/2.06	2.48/2.01/2.45	2.02/1.78/1.90	1.82/1.60/1.74	

a HRS: magnetic-field rotation method (*K* ^RDC*,msy*^ = 0, *K* ^RDC*,mfv*^ = 100 kJ mol^–1^ Hz^–2^, **τ**_θ_^RDC,*mfv*^ = 10 ns, *t* ^*mfv*^ = 30 ns). RMSD values larger than 3 Hz are in (red) italics.

### 5.6 Backbone Conformational Properties for Residues that Show a Large Discrepancy between Calculated and Measured RDC Values

Figure 4 shows the backbone of HEWL, in particular, the parts for which the largest atom-positional differences between the five different X-ray structures are present. These parts are expected to be more flexible. This may lead to a variety of conformations contributing to an RDC value measured in solution and thus to smaller RDC values than would be obtained for a single structure. Residue numbers of atoms for which large differences in calculated RDC values are found are indicated in red for the CA-HA RDCs and in pink for the N–H RDCs.

**Figure 4:**
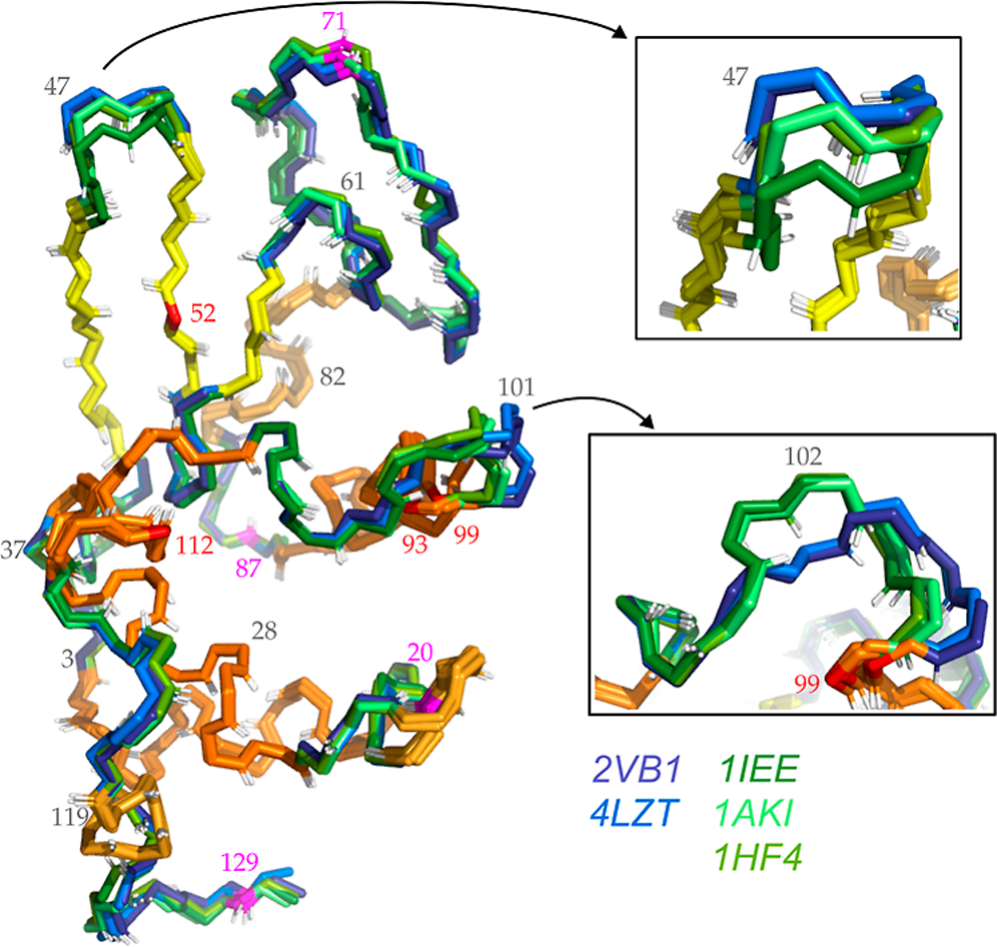
Backbone atoms of the five X-ray structures, 4LZT (light blue), 2VB1 (dark blue), 1IEE (dark green), 1AKI (light green), and 1HF4 (green), superimposed using the atoms of all α- and 3_10_-helices and β-strands. Residue numbers of atoms for which large differences in calculated RDC values are found are indicated in red for the CA-HA RDCs and in pink for the N–H RDCs.

From Tables 1, S3, S7, S11, and S15 containing the CA-HA RDCs, the largest discrepancies between calculated and target RDC values for the 59 residues can be determined. For the residues showing a large discrepancy, the smallest discrepancy among the six RDC values for the residue (three restraint sets for the AT and HRS methods) for the five X-ray structures (4LZT, 2VB1, 1IEE, 1AKI, 1HF4) are found for residues Asp 52 (19 Hz, 19 Hz, 18 Hz, 20 Hz, and 20 Hz), Asn 93 (−11 Hz, −13 Hz, −9 Hz, −14 Hz, −10 Hz), Val 99 (−8 Hz, −8 Hz, −7 Hz, −6 Hz, −6 Hz), and Arg 112 (−11 Hz, −5 Hz, −12 Hz, −5 Hz, −11 Hz). For Asn 93 and Arg 112, the five X-ray structures show a variation larger than 3 Hz. Asp 52, in the middle of a β-strand, and Asn 93 and Arg 112, in the middle of helices, are not in regions in which the backbones of the five crystal structures differ most (Figure 3). Val 99 is in a structurally more variable region. All four RDCs are observed only in one (Table 4.3 of ref 16) of the two CA-HA sets of RDC values. In ref 16 both spectra are described as crowded, and this concerns the ones of Table 4.3 of ref 16 probably most, because each peak was split into four components and also H­(*i*)-CA­(*i* – *1*) peaks were visible. The observed differences between calculated and target RDC values, in particular, those in helices or β-sheets, may be due to assignment difficulties.

From Tables 2, S4, S8, S12, and S16 containing the N–HN RDCs, the largest discrepancies between calculated and target RDC values for the 101 residues can be determined correspondingly. For the residues showing a large discrepancy, the smallest discrepancy among the six RDC values for the residue (three restraint sets for the AT and HRS methods) for the five X-ray structures (4LZT, 2VB1, 1IEE, 1AKI, 1HF4) are found for residues Tyr 20 (−7 Hz, −7 Hz, −7 Hz, −6 Hz, and −7 Hz), Gly 71 (6 Hz, 9 Hz, −1 Hz, 2 Hz, and 0.8 Hz), Asp 87 (−7 Hz, −9 Hz, −5 Hz, −3 Hz, and −4 Hz), and Leu 129 (7 Hz, 7 Hz, 0.6 Hz, 0 Hz, and −2 Hz). With the exception of Asp 87, these residues are in structurally variable regions of the backbone. These discrepancies might be due to different conformations present in solution than in a crystal. In case of a large mobility in solution, the measured RDC value is expected to be closer to zero than the one calculated for a single (X-ray) structure. This is observed for Tyr 20, Gly 71, and Leu 129. For the latter two residues, the five X-ray structures show a variation larger than 3 Hz.

## 6 Discussion and Conclusions

Five sets of 320 RDC values in total, obtained from NMR measurements on uniformly [^13^C,^15^N]-labeled hen egg-white lysozyme (HEWL) in an ether bicelle solution and in an isotropic solution at 35 °C and pH 3.8, were used to investigate their relevance regarding structure determination or refinement of the protein. An RDC *D*_*k*_ ≡ *D*_*k*_1_ *k*_2__ is the result of averaging over the angular distribution *P*(θ_*k*_1_ *k*_2_,*H*_) of the angle θ_*k*_1_ *k*_2_,*H*_ of the vector *r⃗*_*k*_1_ *k*_2__ connecting the atoms *k*_1_ and *k*_2_ of the RDC with the direction of the magnetic field *H⃗*. In this way, small values of ⟨*D*_*k*_⟩, called *residual* dipolar couplings (*R*DCs), of the order of Hz, that is 10^3^–10^4^ smaller than the values of the dipolar couplings *D*_*k*_ themselves, which are of the order of kHz, can be obtained. Unfortunately, the size and shape of the experimentally induced anisotropy or nonuniformity in the rotational distribution *P*(θ_*k*_1_ *k*_2_,*H*_) cannot experimentally be determined. Modeling such an anisotropic or nonuniform rotational distribution in a molecular structure calculation or simulation in such a way that it matches experiment is virtually impossible, in particular due to the difficulty of realistically representing macroscopic conditions at a molecular level of resolution. Therefore, the common approach to calculating RDCs is to minimize the difference between calculated *D*_*k*_ or ⟨*D*_*k*_⟩ RDC values and target *D*_*k*_^0^ RDC values for a given set of (experimentally derived) target RDC values *D*_*k*_^0^, by varying the rotational, orientation distribution *P*(θ_*k*_1_ *k*_2_,*H*_) in one way or the other.

In the AT approach, the orientation distribution is represented in terms of a basis set of five spherical harmonic functions of order 2. The real physical (anisotropic) orientation distribution may, however, differ from the AT one described in terms of spherical harmonics of order 2. Contributions of orders different from 2 are not accounted for in the AT approach, in which the unknown orientation or alignment distribution of the molecule is represented by a 3 × 3 alignment tensor, which depends on 5 parameters, three (Euler) angles plus two nonspherical symmetry parameters for the molecule. The 3 × 3 tensor is symmetric and has trace zero, so it has only 5 independent tensor elements. These represent the reduction of the size of the averaged dipolar couplings from the kHz range to the Hz range and the anisotropy of the orientation distribution of the molecule in terms of the 5 coefficients *a*_*m*_ (*m* = 1–5) of the five spherical harmonic basis functions of order 2, see, e.g., refs 13,15 .

In the HRS approach the orientation distribution is generated in an SD simulation of the rotational motion of a two-atomic magnetic-field vector *r⃗*_*h*_1_ *h*_2__ under the influence of an RDC-restraining force approximately proportional to the difference between calculated (averaged) and target RDC values. This HRS method allows a larger variability of the orientation distribution *P*(θ_*k*_1_ *k*_2_,*H*_) than the AT one.

In both approaches, the mimicking of the reduction of the size of the dipolar coupling due to the averaging of dipolar couplings over the orientation distribution from the kHz range to the Hz range, i.e., by a factor 10^3^–10^4^, by the parameter optimization in the AT approach or by rotational SD simulation of the magnetic-field vector and averaging in the HRS approach, complicates a reliable calculation of RDC values. The AT and HRS methods yield overall a similar picture. As expected, the HRS method allows more variation in the orientation distribution and thus in the calculated RDC values than the AT one.

The data presented for the HEWL illustrate this. How well the calculated RDC values represent the measured RDC values used as target RDC values in the calculation depends on (i) the size of the set of RDC target values used in the calculation, that is, in the fitting of calculated to target RDC values, (ii) the type of the RDC target values used, and (iii) the width of, i.e., the variety within, the distribution of orientations of the RDC target vectors in the molecular structure used in the calculation. For HEWL RDC values were calculated for five single X-ray structures (4LZT, 2VB1, 1IEE, 1AKI, and 1HF4). Apart from a few regions of the backbone, no large structural differences as a function of temperature or crystalline environment (unit cell) are present. The sets of calculated RDC values for these structures do show differences, illustrating the need to include intraprotein motion in the procedure to calculate RDC values. In the current investigation the protein was not simulated using MD simulation, as this would need a currently prohibitively large computational effort to obtain sufficiently converged RDC values in an MD simulation of HEWL in aqueous solution. This is something that could be explored in the future as computing power increases.

A recent study^15^ of a β-heptapeptide solvated in methanol, a molecule much smaller than HEWL and a solvent consisting of larger molecules than water, thus allowing MD simulation of the molecule in solution, led to the same observations regarding the challenge or difficulty of calculating RDC values for a structure using a fit to experimentally measured RDC values as reached here without MD simulation of the protein HEWL. The AT method ignores the coupling between internal and rotational motions of the molecule. In the HRS method the coupling between internal and rotational motion of the molecule is reduced depending on the number *N*_*mfv*_ of SD simulation steps of the magnetic-field vector per molecular configuration; the larger *N*_*mfv*_, the more the coupling is reduced. This renders the AT method, and to a lesser extent the HRS method (depending on the value of *N*_*mfv*_), less reliable for flexible parts of molecules.

Applying the HRS method, artifacts in the calculated RDC values can be detected by considering the angular distributions (θ_*k*_1_ *k*_2_,*H*_/sin­(θ_*k*_1_ *k*_2_,*H*_)) of the RDC or other vectors in the molecule, which should not show peaks at the magic angles. Applying either the AT or the HRS method, the dependence of the calculated RDC values on the type and size of different (sub)­sets of target RDC values should be investigated. This is, however, not a strong test in case the different (sub)­sets of RDC target values contain correlated information.^15^

Why are RDCs much less useful for obtaining structural information on biomolecules such as proteins compared to NOE and ^3^ *J*-coupling calculations and restraining? In other words, which features of RDCs and their calculation differ from the features of the NOE and ^3^ *J*-couplings?

Some features regarding the calculation of RDCs that are different from features of NOEs and ^3^ *J*-couplings are the following.

1. use of (measured) target values to calculate RDC values.
2. The orientation distribution that defines an RDC is unknown and immeasurable.
3. It is impossible to realistically and accurately mimic or represent the orientation-biasing forces (that lead to RDC values not equal to zero) in a simulation at the molecular level of resolution.
4. An RDC is defined as an orientational average over many large, positive, and negative dipolar-coupling (DC) values that lead to a reduction of the averaged DC over more than 3 orders of magnitude to the size of an RDC.

Some features regarding the calculation of NOEs and ^3^ *J*-couplings that are different from the features of RDCs are the following.

1. To calculate an NOE distance or a ^3^ *J*-coupling, no target value is required.
2. An NOE distance or a ^3^ *J*-coupling is defined in terms of a single structure, not in terms of an (orientational) average such as is the case for an RDC. Of course almost any measurement involves averaging, but here the definition makes the difference.
3. An NOE distance or a ^3^ *J*-coupling does not depend on an unknown biasing force present in the measurement or calculation (simulation).
4. An NOE distance or a ^3^ *J*-coupling is an average over many distances (NOE) or torsional-angle values (^3^ *J*-coupling), but the values of the distances or torsional angles do not vary over more than 6 orders of magnitude (−1000 Hz to +1000 Hz for a DC), but over at most 1 order of magnitude, rendering the averaged values vastly less prone to uncertainty.

Yet there also exist similarities between RDCs and ^3^ *J*-couplings. Different orientations of a molecular fragment may be compatible with a single set of RDC values,^33^ just as different torsional angles may be compatible with a single ^3^ *J*-coupling value^18^ due to the multiplicity of the Karplus curve. This feature would suggest the use of local-elevation techniques^34^ to obtain proper (Boltzmann-weighted) sampling of the structures determining the measured RDC or ^3^ *J*-coupling value.

In comparison to other quantities observable by NMR techniques, such as NOESY or ROESY intensities and ^3^ *J*-couplings, measured RDC values are fundamentally less useful for structure determination or refinement of biomolecules. While an NOE intensity can relatively straightforwardly be related to an atom–atom distance and a ^3^ *J*-coupling to a torsional angle using a Karplus-type relation, there exists no direct relationship between an RDC and a molecular configuration due to its definition in terms of a rotational distribution of the angle between the RDC vector and the magnetic field. Since this distribution is immeasurable and cannot be reliably simulated at the molecular level of resolution, and since the slight anisotropy of the rotational distribution of the molecule in the biasing medium reduces the RDC signal from the kHz range to the Hz range, that is, over at least 3 orders of magnitude, the only viable procedure to calculate RDC values for a molecular structure is to apply a procedure in which the difference between calculated and (measured) target RDC values is minimized. These features introduce a much larger uncertainty in calculated RDC values and their effect on the structure determination of refinement of biomolecules than the use of NOE intensities or ^3^ *J*-couplings.

## Supplementary Material





## Data Availability

The GROMOS software for biomolecular simulation is available at https://github.com/biomos under the GPL-2.0 license.

## Notes

Archive file with data in machine-readable format, including HEWL topology, structure files of the 5 crystal structures, and RDC restraint file (ZIP)
^13^C^α^–^13^C′ RDC values as obtained from NMR measurements and as calculated for the X-ray structure 4LZT, ^13^C′-^15^N RDC values as obtained from NMR measurements and as calculated for the X-ray structure 4LZT, equivalent of Tables 1, 2, S1, and S2, but using the 2VB1, 1IEE, 1AKI, or 1HF4 X-ray structure instead of the 4LZT one, the equivalent of Table 6, but for the N–HN RDCs instead of the CA-HA RDCs, backbone hydrogen bonds and secondary-structure assignments for HEWL, ^13^C^α^–^13^C′ RDC values calculated for the X-ray structure 4LZT using restraints to a set of artificial target RDC values, and ^13^C′–^15^N RDC values calculated for the X-ray structure 4LZT using restraints to a set of artificial target RDC values (PDF)
