# Supplementary material for: Use of Measured Residual Dipolar Couplings to Calculate Residual Dipolar Couplings for a Protein Structure: A Case Study Using Hen Egg-White Lysozyme
Source: J Chem Inf Model. 2025 Sep 26;65(19):10418–44. doi: 10.1021/acs.jcim.5c01428 (PMC12529780; doi:10.1021/acs.jcim.5c01428)
Supplement: Supplementary file 2 [file ci5c01428_si_002.pdf]

On the Use of Measured Residual Dipolar Couplings to Calculate Residual Dipolar Couplings for a Protein Structure: A Case Study Using Hen Egg-White Lysozyme

Supporting Information

Maria Pechlaner[#]*, Wilfred F. van Gunsteren[#], Niels Hansen[&] and Lorna J. Smith[†],

[#]Institute of Molecular Physical Sciences, Swiss Federal Institute of Technology, ETH, CH-8093 Zurich, Switzerland

[&]Institute of Thermodynamics and Thermal Process Engineering, University of Stuttgart, D-70569 Stuttgart, Germany

[†]Department of Chemistry, Inorganic Chemistry Laboratory, University of Oxford, South Parks Road, Oxford, OX1 3QR, UK

[*]Corresponding author. Electronic mail: maria.pechlaner@chem.ethz.ch

(Phone: +41 44 6334285)

Version/Date: 16-08-2025

data.zip: Archive file with data in machine-readable format, including HEWL topology, the structure files of the 5 crystal structures and RDC restraint file.

Table S1. $^{13}C^{\alpha}$-$^{13}C'$ RDC values (97) in Hz for HEWL, as obtained from NMR measurements at 308 K and pH = 3.8, Table 4.4 of Ref.[16], and as calculated using three different sets of RDC restraints (*RDC*$_{CAH59}$, *RDC*$_{NH101}$, *RDC*$_{NCAH160}$) for the X-ray structure ***4LZT*** by applying the alignment-tensor method (*AT*: $\tau_D^{RDC} = 0$, $\tau_{AT}^{RDC} = 0$)[13] or the *HRS* ($K^{RDC,msy} = 0$) method[14]. $K^{RDC,mfv} = 100$ kJmol^{-1}Hz^{-2}, $\tau_\theta^{RDC,mfv} = 10$ ns, in $t^{mfv} = 30$ ns SD simulations of the magnetic-field vector. The RDC restraint set *RDC*$_{CAH59}$ contains the RDC values D_k^0 given in the fourth column of Table 1. The RDC restraint set *RDC*$_{NH101}$ contains the RDC values D_k^0 given in the second column of Table 2. The RDC restraint set *RDC*$_{NCAH160}$ is obtained by combining the sets of RDC restraints *RDC*$_{CAH59}$ and *RDC*$_{NH101}$. The values for the RDCs that are *not* part of the (sub)set of RDC restraints applied, are in bold. *RMSD*: Root-mean-square difference (RMSD) between calculated D_k and D_k^0 RDC values calculated over all, *mfv*-restrained and unrestrained, RDCs. *rRMSD*: RMSD-values calculated over the particular (sub)set of *mfv*-restrained RDCs. *urRMSD*: RMSD-values calculated over the unrestrained RDCs. Deviations of RDC-values $D_{k_1k_2}$ (*AT*) or averaged $< D_{k_1k_2} >_{t^{mfv}}$ (*HRS*) from the D_k^0 values larger than 3 Hz are in red. N_{dev}: Number of such deviations. $N_{dev,s}$: Number of RDCs for which the calculated D_k and D_k^0 values have a different sign. These RDC values are in italics.

Residue	Experimental value (Hz), set *RDC*$_{CAC97}$	*AT* Restraint set			*HRS* Restraint set		
		CAH59	*NH101*	*NCAH160*	*CAH59*	*NH101*	*NCAH160*
Lys 1	0.97	**1.4**	**1.0**	**1.2**	**1.4**	**0.9**	**1.3**
Val 2	-2.11	**-1.8**	**-1.7**	**-1.8**	**-1.9**	**-1.6**	**-1.8**
Phe 3	-0.50	**-0.5**	**-0.6**	**-0.5**	**-0.4**	**-0.7**	**-0.5**
Gly 4	1.95	**1.4**	**1.2**	**1.3**	**1.4**	**1.2**	**1.4**
Glu 7	-1.56	**-1.6**	**-1.1**	**-1.3**	**-1.5**	**-1.0**	**-1.5**
Leu 8	1.19	**1.3**	**1.0**	**1.1**	**1.2**	**0.9**	**1.2**
Ala 9	1.19	**1.3**	**1.2**	**1.2**	**1.3**	**1.3**	**1.3**
Ala 10	-1.30	**-1.3**	**-1.3**	**-1.3**	**-1.2**	**-1.4**	**-1.4**
Ala 11	0.13	**0.1**	**0.3**	**0.2**	**0.2**	**0.4**	**0.2**
Met 12	0.83	**0.5**	**0.4**	**0.4**	**0.4**	**0.4**	**0.5**
Lys 13	1.51	**1.4**	**1.2**	**1.3**	**1.5**	**1.2**	**1.4**
Arg 14	-1.94	**-2.5**	**-2.0**	**-2.2**	**-2.4**	**-1.9**	**-2.4**

His 15	1.01	**1.3**	**1.2**	**1.2**	**1.3**	**1.1**	**1.3**
Gly 16	0.55	**1.1**	**1.0**	**1.1**	**1.1**	**1.1**	**1.1**
Asp 18	1.71	**1.2**	**1.2**	**1.2**	**1.3**	**1.2**	**1.3**
Asn 19	-1.81	**-1.9**	**-1.6**	**-1.7**	**-1.8**	**-1.6**	**-1.8**
Tyr 23	-0.67	**-1.3**	**-0.8**	**-1.1**	**-1.3**	**-0.7**	**-1.2**
Ser24	1.34	**1.4**	**1.0**	**1.2**	**1.3**	**0.9**	**1.3**
Leu 25	0.76	**1.6**	**1.5**	**1.5**	**1.6**	**1.6**	**1.6**
Asn 27	-0.39	**0.1**	**-0.3**	**-0.1**	***0.1***	**-0.5**	**-0.0**
Trp 28	1.41	**1.2**	**1.2**	**1.2**	**1.2**	**1.2**	**1.2**
Cys 30	-2.86	**-2.8**	**-2.5**	**-2.7**	**-2.9**	**-2.5**	**-2.8**
Ala 31	1.21	**1.3**	**0.9**	**1.1**	**1.3**	**0.8**	**1.2**
Ala 32	1.67	**1.3**	**1.3**	**1.3**	**1.4**	**1.3**	**1.3**
Lys 33	-0.74	**-0.8**	**-0.6**	**-0.7**	**-0.9**	**-0.5**	**-0.8**
Phe 34	-2.53	**-2.5**	**-2.3**	**-2.4**	**-2.5**	**-2.4**	**-2.5**
Ser 36	-1.04	**-1.3**	**-0.9**	**-1.1**	**-1.2**	**-0.8**	**-1.2**
Asn 37	0.81	**0.8**	**0.8**	**0.8**	**0.8**	**0.9**	**0.8**
Phe 38	0.83	**0.7**	**0.2**	**0.5**	**0.7**	**0.0**	**0.6**
Asn 39	-0.43	**-0.5**	**-0.4**	**-0.4**	**-0.4**	**-0.4**	**-0.5**
Thr 40	-2.42	**-2.3**	**-2.2**	**-2.2**	**-2.4**	**-2.2**	**-2.3**
Gln 41	0.14	**0.3**	**0.4**	**0.3**	**0.2**	**0.5**	**0.3**
Ala 42	-2.63	**-2.4**	**-1.9**	**-2.1**	**-2.3**	**-1.8**	**-2.3**
Asn 44	-1.67	**-2.7**	**-2.3**	**-2.5**	**-2.7**	**-2.2**	**-2.7**
Asn 46	-2.51	**-2.7**	**-2.4**	**-2.5**	**-2.8**	**-2.3**	**-2.6**
Thr 47	-0.95	**-0.4**	**-0.1**	**-0.3**	**-0.3**	**-0.1**	**-0.4**
Asp 48	1.48	**1.3**	**1.0**	**1.2**	**1.4**	**0.9**	**1.3**
Gly 49	-0.60	**-0.9**	**-0.7**	**-0.8**	**-1.0**	**-0.5**	**-0.8**
Ser 50	-1.22	**-1.3**	**-0.8**	**-1.0**	**-1.1**	**-0.7**	**-1.2**
Thr 51	-1.57	**-1.1**	**-1.1**	**-1.1**	**-1.2**	**-1.1**	**-1.1**
Tyr 53	-1.29	**-2.5**	**-2.3**	**-2.4**	**-2.6**	**-2.2**	**-2.5**
Leu 56	-2.63	**-2.3**	**-1.8**	**-2.0**	**-2.3**	**-1.7**	**-2.2**
Gln 57	-0.66	**-0.5**	**-0.2**	**-0.3**	**-0.4**	**-0.1**	**-0.4**
Asn 59	-1.71	**-0.5**	**-0.5**	**-0.4**	**-0.4**	**-0.6**	**-0.5**
Ser 60	-2.19	**-1.5**	**-1.0**	**-1.2**	**-1.4**	**-0.9**	**-1.4**
Arg 61	1.21	**1.4**	**1.3**	**1.3**	**1.4**	**1.4**	**1.5**
Cys 64	-2.38	**-2.0**	**-2.0**	**-2.0**	**-2.2**	**-2.0**	**-2.1**
Asn 65	0.92	**1.4**	**1.3**	**1.4**	**1.5**	**1.3**	**1.4**
Asp 66	-2.07	**-2.5**	**-2.4**	**-2.4**	**-2.6**	**-2.4**	**-2.5**
Gly 67	0.08	**0.3**	**0.4**	**0.4**	**0.3**	**0.6**	**0.4**
Arg 68	1.42	**1.4**	**1.1**	**1.2**	**1.4**	**1.0**	**1.3**
Gly 71	-2.77	**-1.4**	**-1.1**	**-1.2**	**-1.5**	**-0.9**	**-1.3**
Asn 74	0.44	**0.4**	**0.2**	**0.2**	**0.2**	**0.2**	**0.3**
Leu 75	-2.30	**-1.7**	**-1.3**	**-1.5**	**-1.8**	**-1.1**	**-1.6**
Cys 76	1.80	**1.2**	**0.8**	**1.0**	**1.2**	**0.6**	**1.1**
Asn 77	0.62	**0.6**	**0.7**	**0.6**	**0.6**	**0.7**	**0.6**
Pro 79	0.26	***-0.4***	***-0.4***	***-0.4***	***-0.3***	***-0.5***	***-0.5***
Cys 80	-0.23	**-0.4**	**-0.1**	**-0.3**	**-0.4**	**-0.0**	**-0.4**
Ser 81	-1.71	**-1.7**	**-1.5**	**-1.6**	**-1.8**	**-1.4**	**-1.7**
Ala 82	-0.80	**-0.5**	**-0.7**	**-0.6**	**-0.5**	**-0.9**	**-0.6**
Leu 83	1.12	**0.7**	**0.8**	**0.8**	**0.8**	**0.9**	**0.8**
Leu 84	-0.72	**-0.5**	**-0.2**	**-0.3**	**-0.5**	**-0.0**	**-0.4**

Ser 85	-2.60	-2.0	-1.8	-1.9	-2.0	-1.9	-2.1
Ile 88	2.38	0.8	0.9	0.9	0.9	1.0	0.9
Thr 89	-1.36	-2.3	-1.7	-2.0	-2.3	-1.6	-2.2
Ala 90	1.77	1.4	1.2	1.3	1.4	1.1	1.4
Ser 91	1.06	0.7	0.3	0.4	0.6	0.2	0.6
Val 92	1.08	0.0	0.3	0.2	0.2	0.3	0.1
Asn 93	-1.11	-1.2	-0.8	-1.0	-1.3	-0.7	-1.1
Cys 94	0.75	1.0	0.6	0.8	1.0	0.4	0.9
Ala 95	1.20	1.4	1.2	1.3	1.4	1.1	1.4
Lys 96	-1.79	-2.0	-1.5	-1.7	-1.9	-1.3	-1.9
Lys 97	0.79	1.1	1.0	1.0	1.1	1.1	1.1
Ile 98	-1.68	*0.9*	*0.4*	*0.6*	*0.8*	*0.2*	*0.7*
Val 99	1.81	1.1	1.2	1.2	1.3	1.2	1.2
Ser 100	-2.11	-2.5	-2.0	-2.2	-2.5	-1.8	-2.4
Gly 102	0.52	1.4	1.3	1.3	1.5	1.3	1.4
Gly 104	1.94	1.4	1.3	1.4	1.5	1.3	1.4
Met 105	0.41	0.6	0.6	0.6	0.5	0.7	0.6
Asn 106	-0.99	-1.0	-1.2	-1.1	-1.1	-1.4	-1.1
Ala 107	1.44	1.2	1.2	1.2	1.3	1.3	1.3
Ala 110	-1.26	-0.7	-0.9	-0.9	-0.9	-1.0	-0.8
Trp 111	1.12	1.4	1.1	1.3	1.4	1.0	1.3
Asn 113	-2.61	-2.5	-1.9	-2.2	-2.4	-1.8	-2.4
Arg 114	0.28	*-1.0*	*-0.9*	*-1.0*	*-1.2*	*-0.8*	*-1.0*
Cys 115	1.25	1.5	1.1	1.3	1.4	1.0	1.4
Lys 116	-0.35	*0.1*	-0.2	-0.0	*0.1*	-0.3	-0.0
Thr 118	-0.81	-0.7	-0.5	-0.6	-0.8	-0.4	-0.6
Val 120	-0.92	-0.9	-0.6	-0.8	-1.0	-0.4	-0.8
Gln 121	1.11	0.6	0.8	0.7	0.7	0.8	0.7
Ala 122	0.73	1.0	0.5	0.7	0.9	0.3	0.8
Trp 123	0.43	0.7	0.7	0.7	0.7	0.8	0.7
Ile 124	-3.36	-2.9	-2.5	-2.7	-2.9	-2.5	-2.8
Arg 125	0.76	0.6	0.7	0.7	0.7	0.7	0.7
Gly 126	0.95	1.1	0.7	0.9	1.1	0.6	1.0
Cys 127	0.43	0.9	0.9	0.9	0.9	1.0	1.0
Arg 128	-0.62	-1.2	-0.9	-1.0	-1.1	-0.9	-1.2
RMSD		0.6	0.6	0.5	0.5	0.6	0.5
rRMSD		-	-	-	-	-	-
urRMSD		0.6	0.6	0.5	0.5	0.6	0.5
N_{dev}		0	0	0	0	0	0
$N_{dev,s}$		4	3	3	5	3	3

Table S2. ^{13}C'-^{15}N RDC values (45) in Hz for HEWL, as obtained from NMR measurements at 308 K and pH = 3.8, Table 4.5 of Ref.[16], and as calculated using three different sets of RDC restraints (*RDC*$_{CAH59}$, *RDC*$_{NH101}$, *RDC*$_{NCAH160}$) for the X-ray structure ***4LZT*** by applying the alignment-tensor method (*AT*: $\tau_D^{RDC} = 0$, $\tau_{AT}^{RDC} = 0$)[13] or the *HRS* ($K^{RDC,msy} = 0$) method[14]. $K^{RDC,mfv} = 100$ kJmol^{-1}Hz^{-2}, $\tau_\theta^{RDC,mfv} = 10$ ns, in $t^{mfv} = 30$ ns SD simulations of the magnetic-field vector. The RDC restraint set *RDC*$_{CAH59}$ contains the RDC values D_k^0 given in the fourth column of Table 1. The RDC restraint set *RDC*$_{NH101}$ contains the RDC values D_k^0 given in the second column of Table 2. The RDC restraint set *RDC*$_{NCAH160}$ is obtained by combining the sets of RDC restraints *RDC*$_{CAH59}$ and *RDC*$_{NH101}$. The values for the RDCs that are *not* part of the (sub)set of RDC restraints applied, are in bold. *RMSD*: Root-mean-square difference (RMSD) between calculated D_k and D_k^0 RDC values calculated over all, *mfv*-restrained and unrestrained, RDCs. *rRMSD*: RMSD-values calculated over the particular (sub)set of *mfv*-restrained RDCs. *urRMSD*: RMSD-values calculated over the unrestrained RDCs. Deviations of RDC-values $D_{k_1k_2}$ (*AT*) or averaged $< D_{k_1k_2} >_{t^{mfv}}$ (*HRS*) from the D_k^0 values larger than 3 Hz are in red. N_{dev}: Number of such deviations. $N_{dev,s}$: Number of RDCs for which the calculated D_k and the D_k^0 values have a different sign. These RDC values are in italics.

Residue	Experimental value (Hz), set *RDC*$_{CN45}$ D_k^0	*AT* Restraint set			*HRS* Restraint set		
		CAH59	*NH101*	*NCAH160*	*CAH59*	*NH101*	*NCAH160*
Gly 4	-0.55	**-0.5**	**-0.2**	**-0.3**	**-0.5**	**-0.1**	**-0.4**
Glu 7	0.20	**0.1**	***-0.0***	**0.0**	**0.1**	***-0.1***	**0.1**
Leu 8	0.67	**0.6**	**0.7**	**0.7**	**0.6**	**0.8**	**0.7**
Ala 9	-0.38	***0.0***	**-0.1**	**-0.0**	***0.0***	**-0.1**	***0.0***
Ala 10	0.42	**0.5**	**0.6**	**0.6**	**0.6**	**0.6**	**0.6**
Ala 11	-0.95	**-0.9**	**-0.7**	**-0.8**	**-0.9**	**-0.7**	**-0.9**
Met 12	2.41	**1.9**	**1.7**	**1.8**	**2.0**	**1.6**	**1.9**
Lys 13	-0.78	**-0.9**	**-0.7**	**-0.8**	**-0.9**	**-0.6**	**-0.9**
Arg 14	1.07	**0.6**	**0.4**	**0.5**	**0.7**	**0.3**	**0.6**
Gly 16	-0.95	**-1.0**	**-0.9**	**-0.9**	**-1.0**	**-1.0**	**-1.0**
Trp 28	1.37	**1.6**	**1.3**	**1.5**	**1.6**	**1.3**	**1.6**

Ala 31	-0.23	**-0.2**	**-0.2**	**-0.2**	**-0.1**	**-0.3**	**-0.2**
Ala 32	0.38	**0.9**	**0.9**	**0.9**	**0.9**	**1.0**	**0.9**
Lys 33	-0.49	**-0.4**	**-0.4**	**-0.4**	**-0.4**	**-0.4**	**-0.4**
Asn 37	-1.12	**-0.9**	**-0.9**	**-0.9**	**-1.0**	**-0.9**	**-0.9**
Phe 38	1.72	**1.3**	**1.2**	**1.2**	**1.4**	**1.1**	**1.3**
Asn 39	-0.78	**-0.8**	**-0.7**	**-0.8**	**-0.8**	**-0.7**	**-0.8**
Thr 40	-1.41	**-0.9**	**-0.8**	**-0.9**	**-0.9**	**-0.8**	**-0.9**
Gln 41	-0.26	**-0.5**	**-0.3**	**-0.4**	**-0.5**	**-0.1**	**-0.5**
Thr 47	-1.13	**-0.6**	**-0.5**	**-0.5**	**-0.6**	**-0.5**	**-0.6**
Arg 61	1.21	**1.2**	**1.2**	**1.2**	**1.3**	**1.3**	**1.2**
Arg 68	-0.64	**-0.4**	**-0.3**	**-0.3**	**-0.4**	**-0.2**	**-0.4**
Leu 75	-0.57	**-0.3**	**-0.4**	**-0.3**	**-0.3**	**-0.4**	**-0.3**
Cys 76	1.35	**1.6**	**1.4**	**1.5**	**1.6**	**1.5**	**1.6**
Asn 77	-0.81	**-1.0**	**-0.9**	**-0.9**	**-1.0**	**-0.9**	**-1.0**
Ser 81	-1.23	**-0.9**	**-0.9**	**-0.9**	**-1.0**	**-0.9**	**-1.0**
Leu 83	-0.89	**-0.7**	**-0.5**	**-0.6**	**-0.7**	**-0.4**	**-0.7**
Leu 84	-0.63	**-0.6**	**-0.6**	**-0.6**	**-0.6**	**-0.6**	**-0.6**
Ala 90	-0.78	**-0.2**	**-0.3**	**-0.3**	**-0.3**	**-0.3**	**-0.3**
Asn 93	-0.28	**-0.4**	**-0.3**	**-0.4**	**-0.4**	**-0.4**	**-0.4**
Ala 95	-0.40	**-0.6**	**-0.6**	**-0.6**	**-0.6**	**-0.6**	**-0.6**
Lys 96	1.14	**0.8**	**0.9**	**0.9**	**0.9**	**1.0**	**0.9**
Lys 97	-1.40	**-0.9**	**-0.9**	**-0.9**	**-0.9**	**-0.9**	**-0.9**
Gly 104	0.60	**1.3**	**1.1**	**1.2**	**1.3**	**1.1**	**1.3**
Met 105	-0.05	**-0.2**	**-0.3**	**-0.2**	**-0.2**	**-0.3**	**-0.2**
Ala 110	-1.01	**-0.9**	**-0.9**	**-0.9**	**-0.9**	**-0.9**	**-0.9**
Trp 111	1.13	**1.4**	**1.1**	**1.2**	**1.4**	**1.0**	**1.3**
Asn 113	-0.28	**-0.3**	**-0.3**	**-0.3**	**-0.2**	**-0.4**	**-0.3**
Arg 114	-1.14	**-0.8**	**-0.8**	**-0.8**	**-0.9**	**-0.8**	**-0.8**
Cys 115	0.97	**1.0**	**0.6**	**0.8**	**0.9**	**0.6**	**0.9**
Gln 121	-0.78	**-0.9**	**-0.8**	**-0.9**	**-0.9**	**-0.9**	**-0.9**
Ala 122	1.68	**1.1**	**1.0**	**1.0**	**1.1**	**1.0**	**1.1**
Arg 125	1.26	**1.1**	**0.8**	**1.0**	**1.1**	**0.8**	**1.0**
Gly 126	0.52	**1.0**	**0.8**	**0.8**	**0.9**	**0.7**	**0.9**
Cys 127	-0.67	**-0.9**	**-0.7**	**-0.8**	**-0.9**	**-0.6**	**-0.8**
RMSD		**0.3**	**0.3**	**0.3**	**0.3**	**0.4**	**0.3**
rRMSD		-	-	-	-	-	-
urRMSD		**0.3**	**0.3**	**0.3**	**0.3**	**0.4**	**0.3**
N_{dev}		**0**	**0**	**0**	**0**	**0**	**0**
$N_{dev,s}$		**0**	**1**	**0**	**1**	**1**	**1**

Table S3. $^{13}C^{\alpha}$-$^{1}H^{\alpha}$ RDC values (38, 39) in Hz for HEWL, as obtained from NMR measurements at 308 K and pH = 3.8 using two different pulse sequences as given in Tables 4.2 and 4.3 of Ref.[16], and as calculated using three different sets of RDC restraints (RDC_{CAH59}, RDC_{NH101}, $RDC_{NCAH160}$) for the X-ray structure ***2VB1*** by applying the alignment-tensor method (*AT*: $\tau_D^{RDC} = 0$, $\tau_{AT}^{RDC} = 0$)[13] or the *HRS* ($K^{RDC,msy} = 0$) method[14], $K^{RDC,mfv} = 100$ kJmol^{-1}Hz^{-2}, $\tau_\theta^{RDC,mfv} = 10$ ns, in $t^{mfv} = 30$ ns SD simulations of the magnetic-field vector. The (RDC restraint) set RDC_{CAH59} contains the RDC values D_k^0 given in the fourth column. The RDC restraint set RDC_{NH101} contains the RDC values D_k^0 given in the second column of Table 2. The RDC restraint set $RDC_{NCAH160}$ is obtained by combining the sets of RDC restraints RDC_{CAH59} and RDC_{NH101}. In case two experimental values are available, the D_k^0 RDC values used in the calculations are the average of the two experimental values. The values for the RDCs that are *not* part of the (sub)set of RDC restraints applied, are in bold. *RMSD*: Root-mean-square difference (RMSD) between calculated D_k and D_k^0 RDC values calculated over all, *mfv*-restrained and unrestrained, RDCs. *rRMSD*: RMSD-values calculated over the particular (sub)set of *mfv*-restrained RDCs. *urRMSD*: RMSD-values calculated over the unrestrained RDCs. Deviations of RDC values $D_{k_1k_2}$ (*AT*) or averaged $< D_{k_1k_2} >_{t^{mfv}}$ (*HRS*) from the D_k^0 values larger than 3 Hz are in red. N_{dev}: Number of such deviations. $N_{dev,s}$: Number of RDCs for which the calculated D_k and the D_k^0 values have a different sign. These RDC values are in italics.

Residue	Experimental value (Hz)			*AT*			*HRS*		
	Table 4.2[16]	Table 4.3[16]	D_k^0	*CAH59*	*NH101*	*NCAH160*	*CAH59*	*NH101*	*NCAH160*
Val 2	-5.30	-	-5.30	-10.0	**-5.6**	-7.6	-9.3	**-5.7**	-9.2
Phe 3	-	8.85	8.85	8.8	**8.2**	8.4	8.1	**8.4**	9.7
Cys 6	-	15.30	15.30	14.8	**12.3**	13.4	15.6	**12.5**	15.3
Glu 7	-	-6.52	-6.52	-4.9	**-2.7**	-3.9	-6.5	**-2.8**	-4.3
Leu 8	-	3.17	3.17	5.3	**1.2**	3.3	5.2	**1.4**	4.4
Met 12	-20.12	-22.20	-21.16	-21.0	**-20.1**	-20.3	-20.8	**-20.3**	-22.2

Lys 13	10.26	10.33	10.30	7.6	**7.7**	7.6	8.2	**7.8**	8.4
His 15	15.60	-	15.60	16.6	**14.1**	15.3	16.0	**14.4**	16.7
Leu 17	-	-10.66	-10.66	-9.6	**-5.3**	-7.1	-7.9	**-5.4**	-9.3
Asp 18	-	19.61	19.61	14.7	**13.9**	14.2	13.9	**13.9**	15.2
Tyr 23	-	13.81	13.81	14.5	**10.1**	12.2	14.0	**10.4**	14.3
Leu 25	-18.58	-	-18.58	-17.9	**-17.8**	-17.6	-17.7	**-17.9**	-19.1
Trp 28	-28.74	-28.51	-28.62	-27.4	**-21.4**	-24.2	-28.0	**-21.7**	-27.0
Val 29	5.17	-	5.17	7.0	**1.8**	4.3	6.8	**2.0**	6.1
Cys 30	9.83	8.84	9.34	5.8	**7.6**	6.9	7.7	**7.5**	6.2
Lys 33	13.60	-	13.60	13.1	**11.0**	12.0	13.5	**11.2**	13.8
Phe 34	-	15.36	15.36	12.9	**13.1**	13.0	14.4	**13.1**	13.2
Glu 35	-	-5.65	-5.65	-3.0	**-0.6**	-1.8	-4.2	**-0.5**	-1.9
Asn 39	-6.88	-4.05	-5.46	-7.4	**-3.9**	-5.6	-7.9	**-3.9**	-6.3
Thr 40	15.69	15.81	15.75	15.5	**12.4**	13.7	16.2	**12.5**	15.2
Ala 42	-	18.93	18.93	16.3	**15.1**	15.6	17.2	**15.1**	16.6
Asn 44	21.03	-	21.03	15.3	**14.9**	15.1	16.5	**14.9**	15.6
Thr 47	-	12.40	12.40	14.6	**9.8**	12.0	14.8	**10.1**	14.4
Asp 48	-17.97	-18.29	-18.13	-17.5	**-13.2**	-15.3	-18.9	**-13.3**	-16.9
Thr 51	16.96	-	16.96	16.5	**15.0**	15.7	16.4	**15.1**	16.7
Asp 52	-	-5.43	-5.43	*13.8*	***13.9***	*13.9*	*15.2*	***13.8***	*14.1*
Leu 56	-0.83	-	-0.83	-3.8	**-4.8**	-4.1	-3.2	**-4.8**	-4.7
Gln 57	6.72	-	6.72	9.4	**4.5**	6.7	9.5	**4.6**	8.8
Asn 59	17.60	-	17.60	15.9	**14.4**	15.0	17.1	**14.4**	16.2
Trp 62	-19.22	-22.42	-20.82	-24.9	**-19.8**	-22.0	-24.0	**-20.1**	-25.2
Trp 63	17.30	-	17.30	15.7	**15.0**	15.3	16.9	**15.0**	16.0
Cys 64	18.24	-	18.24	15.9	**15.0**	15.4	15.6	**15.1**	16.3
Asn 65	0.76	-	0.76	1.0	**2.6**	1.8	*-0.0*	**2.7**	2.1
Pro 70	5.72	-	5.72	*-3.1*	***-5.1***	*-4.0*	*-2.8*	***-5.1***	*-4.2*
Ile 78	-	17.81	17.81	16.0	**12.6**	14.2	15.4	**12.8**	16.0
Ser 81	14.90	-	14.90	15.2	**14.1**	14.7	15.5	**14.2**	15.2
Leu 84	-	5.15	5.15	6.1	**2.3**	4.3	6.0	**2.5**	5.3
Ser 85	-	13.38	13.38	12.8	**8.0**	10.2	13.0	**8.2**	12.5
Ile 88	-14.50	-15.25	-14.88	-10.3	**-9.3**	-10.0	-11.6	**-9.5**	-10.5
Thr 89	-0.08	-3.00	-1.54	-7.3	**-9.8**	-8.8	-7.9	**-9.9**	-8.4
Ala 90	-	17.24	17.24	15.7	**15.3**	15.5	16.7	**15.3**	15.9
Val 92	14.69	-	14.69	15.8	**12.2**	14.0	15.2	**12.5**	15.8
Asn 93	-	10.98	10.98	*-2.1*	***-4.3***	*-3.5*	*-2.7*	***-4.4***	*-2.8*
Lys 96	-1.72	-	-1.72	-1.5	**-5.2**	-3.3	-1.6	**-5.1**	-2.6
Ile 98	-26.52	-27.59	-27.06	-31.5	**-26.0**	-28.5	-31.3	**-26.3**	-31.8
Val 99	-	18.49	18.49	9.4	**8.9**	9.1	8.1	**9.1**	10.2
Ser 100	-8.22	-	-8.22	-0.5	**-4.6**	-2.7	-0.8	**-4.5**	-1.5
Trp 108	-10.09	-	-10.09	-8.9	**-11.7**	-10.4	-9.4	**-11.7**	-10.1
Val 109	14.56	19.08	16.82	14.1	**11.7**	12.7	14.6	**11.9**	14.6
Trp 111	-22.31	-22.56	-22.44	-21.0	**-15.2**	-17.8	-20.1	**-15.4**	-20.7
Arg 112	-	12.73	12.73	6.8	**5.6**	5.9	6.3	**5.5**	6.7
Cys 115	-	-21.39	-21.39	-19.1	**-14.2**	-16.3	-17.8	**-14.4**	-19.2
Lys 116	8.87	3.30	6.08	10.9	**10.6**	10.6	9.9	**10.6**	11.3
Thr 118	12.69	-	12.69	10.1	**5.7**	7.9	9.9	**6.6**	9.5
Asp 119	17.09	15.66	16.37	16.2	**14.7**	15.3	17.1	**14.7**	16.4
Val 120	13.71	13.50	13.65	14.4	**12.9**	13.5	14.7	**12.9**	14.6

Gln 121	12.41	9.50	10.95	10.1	**17.2**	8.8	10.1	**7.4**	9.6
Ala 122	-	-30.56	-30.56	-31.3	**-26.5**	-28.7	-32.0	**-26.8**	-31.6
Cys 127	14.73	16.44	15.58	13.9	**13.1**	13.4	15.3	**13.1**	14.4
RMSD				4.4	**5.3**	4.7	4.4	**5.3**	4.4
rRMSD				4.4	-	4.7	4.4	-	4.4
urRMSD				-	**5.3**	-	-	**5.3**	-
N_{dev}				15	**32**	22	15	**33**	18
$N_{dev,s}$				3	**3**	3	4	**3**	3

Table S4. ^{15}N-^{1}H^{N} RDC values (101) in Hz for HEWL, as obtained from NMR measurements at 308 K and pH = 3.8, Table 4.1 of Ref.[16], and as calculated using three different sets of RDC restraints (RDC_{CAH59}, RDC_{NH101}, $RDC_{NCAH160}$) for the X-ray structure ***2VB1*** by applying the alignment-tensor method (*AT*: $\tau_D^{RDC} = 0$, $\tau_{AT}^{RDC} = 0$)[13] or the *HRS* ($K^{RDC,msy} = 0$) method[14], $K^{RDC,mfv} = 100$ kJmol^{-1}Hz^{-2}, $\tau_\theta^{RDC,mfv} = 10$ ns, in $t^{mfv} = 30$ ns SD simulations of the magnetic-field vector. The RDC restraint set RDC_{CAH59} contains the RDC values D_k^0 given in the fourth column of Table 1. The RDC restraint set RDC_{NH101} contains the RDC values D_k^0 given in the second column. The RDC restraint set $RDC_{NCAH160}$ is obtained by combining the sets of RDC restraints RDC_{CAH59} and RDC_{NH101}. The values for the RDCs that are *not* part of the (sub)set of RDC restraints applied, are in bold. *RMSD*: Root-mean-square difference (RMSD) between calculated D_k and D_k^0 RDC values calculated over all, *mfv*-restrained and unrestrained, RDCs. *rRMSD*: RMSD-values calculated over the particular (sub)set of *mfv*-restrained RDCs. *urRMSD*: RMSD-values calculated over the unrestrained RDCs. Deviations of RDC values $D_{k_1k_2}$ (*AT*) or averaged $< D_{k_1k_2} >_{t^{mfv}}$ (*HRS*) from the D_k^0 values larger than 3 Hz are in red. N_{dev}: Number of such deviations. $N_{dev,s}$: Number of RDCs for which the calculated D_k and the D_k^0 values have a different sign. These RDC values are in italics.

Residue	Experimental value (Hz), target D_k^0	*AT*			*HRS*		
		CAH59	*NH101*	*NCAH160*	*CAH59*	*NH101*	*NCAH160*
Val 2	-1.78	**-0.6**	-1.7	-1.2	**-1.0**	-1.7	-1.1
Phe 3	7.52	**10.4**	7.4	8.8	**10.2**	7.5	10.1
Gly 4	-6.92	**-6.5**	-5.8	-6.1	**-5.9**	-5.9	-6.8
Arg 5	-6.47	**-3.2**	-3.5	-3.5	**-3.9**	-3.5	-3.1
Cys 6	-3.68	**-5.2**	-5.7	-5.5	**-6.1**	-5.6	-5.3
Glu 7	-8.56	**-8.6**	-8.2	-8.4	**-8.9**	-8.2	-8.7
Leu 8	-7.85	**-7.5**	-7.1	-7.4	**-7.8**	-7.1	-7.5
Ala 9	-4.27	**-3.2**	-3.8	-3.6	**-4.0**	-3.7	-3.2
Ala 11	-8.64	**-8.4**	-8.1	-8.2	**-8.8**	-8.1	-8.5
Lys 13	-3.36	**-1.9**	-2.9	-2.5	**-2.8**	-2.9	-2.0
Arg 14	-7.36	**-7.3**	-7.3	-7.3	**-8.0**	-7.3	-7.4
Gly 16	12.38	**12.7**	9.7	11.0	**12.2**	9.8	12.7

Leu 17	-4.59	**-3.9**	-4.3	-4.1	**-4.6**	-4.3	-4.3
Asn 19	-5.96	**-8.5**	-8.0	-8.2	**-8.4**	-8.0	-8.6
Tyr 20	0.61	***-6.7***	*-6.6*	*-6.6*	***-7.5***	*-6.6*	*-6.9*
Arg 21	-6.77	**-7.4**	-6.9	-7.1	**-6.9**	-7.0	-7.6
Gly 22	-2.70	**-2.5**	-2.9	-2.7	**-1.8**	-3.0	-3.0
Tyr 23	0.23	***-5.4***	*-3.6*	*-4.6*	***-5.4***	*-3.7*	*-5.1*
Ser24	-6.59	**-8.3**	-6.4	-7.3	**-7.9**	-6.6	-8.4
Gly 26	4.19	**0.4**	1.6	1.2	**0.7**	1.7	0.7
Asn 27	-5.76	**-4.7**	-3.9	-4.1	**-4.5**	-3.8	-4.7
Trp 28	5.29	**2.6**	2.3	2.6	**3.2**	2.4	2.6
Val 29	3.56	**2.8**	3.5	3.3	**3.2**	3.6	3.1
Ala 31	-3.83	**-4.4**	-4.0	-4.1	**-4.1**	-3.9	-4.5
Ala 32	4.34	**2.8**	2.6	2.8	**3.4**	2.6	2.9
Lys 33	0.35	***-1.2***	*-0.0*	*-0.5*	***-1.0***	0.0	*-1.0*
Phe 34	-4.22	**-7.4**	-6.4	-6.7	**-7.6**	-6.3	-7.4
Glu 35	3.73	**2.2**	1.7	2.1	**2.9**	1.8	2.2
Ser 36	7.51	**10.2**	9.7	10.0	**10.7**	9.8	10.6
Asn 37	-7.43	**-8.0**	-6.2	-7.0	**-8.2**	-6.3	-8.2
Phe 38	-0.76	**-1.3**	-2.3	-1.8	**-1.8**	-2.3	-1.8
Asn 39	-2.69	**-3.3**	-3.4	-3.4	**-2.8**	-3.5	-3.9
Thr 40	6.48	**8.7**	5.9	7.2	**8.5**	5.9	8.3
Gln 41	6.78	**6.7**	4.8	5.6	**5.9**	4.9	6.8
Ala 42	10.72	**9.6**	8.9	9.3	**10.2**	9.1	9.9
Thr 43	-6.94	**-6.8**	-6.9	-6.9	**-7.5**	-6.9	-6.9
Asn 44	-8.88	**-7.4**	-7.3	-7.4	**-7.9**	-7.3	-7.5
Arg 45	-7.32	**-5.3**	-5.8	-5.6	**-6.2**	-5.7	-5.4
Asn 46	-4.82	**-8.8**	-8.1	-8.4	**-8.6**	-8.1	-8.9
Thr 47	4.71	**0.5**	2.4	1.4	**0.5**	2.4	1.1
Asp 48	14.01	**15.8**	14.1	14.9	**16.0**	14.2	16.2
Gly 49	1.82	***-1.3***	*-2.1*	*-1.8*	***-2.1***	*-2.0*	*-1.2*
Thr 51	-7.92	**-8.5**	-8.2	-8.4	**-8.8**	-8.2	-8.6
Gly 54	-6.58	**-6.2**	-5.3	-5.6	**-6.3**	-5.3	-6.2
Ile 55	-7.54	**-8.5**	-7.3	-7.9	**-8.1**	-7.5	-8.7
Leu 56	-0.14	**-3.2**	-1.4	-2.3	**-3.2**	-1.5	-2.8
Gln 57	-3.19	**-4.3**	-4.2	-4.2	**-4.5**	-4.4	-4.8
Ile 58	-3.83	**-2.9**	-3.1	-3.0	**-2.7**	-3.2	-3.5
Asn 59	-3.85	**-6.4**	-4.2	-5.2	**-6.6**	-4.3	-6.2
Ser 60	-7.82	**-8.7**	-8.2	-8.4	**-8.8**	-8.2	-8.8
Trp 63	-8.15	**-8.4**	-7.9	-8.0	**-8.5**	-7.8	-8.5
Asn 65	1.18	**0.3**	*-0.7*	*-0.2*	**1.1**	*-0.7*	*-0.2*
Asp 66	-0.81	**-2.9**	-3.1	-3.0	**-2.7**	-3.2	-3.4
Gly 67	-7.13	**-7.3**	-5.6	-6.3	**-7.5**	-5.6	-7.2
Arg 68	13.60	**11.0**	10.8	10.9	**11.4**	10.9	11.6
Thr 69	-3.43	**-7.4**	-7.0	-7.1	**-6.9**	-7.0	-7.7
Gly 71	2.44	**12.0**	11.5	11.6	**12.0**	11.6	12.6
Arg 73	-1.26	***2.6***	*1.8*	*2.0*	***2.0***	*1.8*	*2.9*
Asn 74	-7.40	**-8.3**	-7.8	-8.0	**-8.1**	-7.8	-8.4
Cys 76	-4.97	**-4.9**	-4.7	-4.7	**-4.5**	-4.7	-5.1
Ile 78	-1.37	**-4.7**	-4.5	-4.5	**-4.0**	-4.6	-5.1
Cys 80	16.06	**16.3**	13.8	15.0	**16.6**	14.0	16.5

Ser 81	14.37	**12.2**	10.9	11.4	**11.9**	10.9	12.7
Ala 82	7.74	**10.0**	7.2	8.4	**9.4**	7.3	9.9
Leu 83	15.17	**16.6**	13.8	15.1	**16.9**	14.0	16.8
Leu 84	5.43	**1.8**	2.6	2.1	**1.6**	2.5	2.3
Ser 86	-4.45	**-6.3**	-3.5	-4.9	**-6.1**	-3.7	-6.0
Asp 87	12.03	**0.4**	2.6	1.5	**0.5**	2.5	1.0
Thr 89	-2.93	**-5.5**	-5.0	-5.2	**-5.7**	-5.1	-5.9
Ser 91	-0.10	**-2.1**	-2.9	-2.5	**-2.6**	-2.9	-2.5
Val 92	-4.32	**-4.7**	-4.4	-4.5	**-4.8**	-4.5	-5.1
Asn 93	-6.06	**-7.9**	-6.3	-7.0	**-8.2**	-6.4	-8.1
Cys 94	-5.79	**-5.7**	-5.6	-5.6	**-6.4**	-5.6	-6.0
Ala 95	-2.42	**-2.2**	-2.8	-2.5	**-2.6**	-2.9	-2.7
Lys 96	-6.08	**-6.6**	-5.5	-6.0	**-6.6**	-5.7	-6.9
Lys 97	-7.62	**-7.8**	-6.4	-7.0	**-8.2**	-6.5	-8.0
Ile 98	-4.26	**-3.4**	-3.9	-3.7	**-4.0**	-3.9	-3.8
Ser 100	-4.10	**-8.0**	-6.4	-7.1	**-8.3**	-6.5	-8.2
Gly 102	8.96	**9.4**	6.7	8.0	**9.8**	6.8	9.0
Asn 103	-7.26	**-7.3**	-4.8	-6.0	**-7.1**	-5.0	-7.2
Gly 104	-4.67	***3.0***	*1.5*	*2.3*	***3.6***	*1.5*	*2.5*
Met 105	9.39	**5.6**	5.9	5.8	**6.0**	6.0	5.9
Asn 106	-7.82	**-7.0**	-5.6	-6.2	**-7.2**	-5.6	-6.9
Ala 107	-1.63	**-2.8**	-2.8	-2.7	**-2.2**	-2.8	-2.9
Trp 108	3.79	**3.0**	3.8	3.5	**3.4**	3.9	3.4
Val 109	7.54	**3.3**	5.0	4.2	**3.4**	5.0	4.0
Trp 111	9.71	**5.9**	6.1	5.9	**5.7**	6.2	6.5
Arg 112	8.40	**8.5**	9.0	8.7	**8.6**	9.1	9.2
Asn 113	1.87	***-3.8***	*-1.1*	*-2.4*	***-3.7***	*-1.3*	*-3.3*
Cys 115	14.32	**12.9**	9.8	11.2	**12.5**	9.9	12.8
Lys 116	12.40	**10.4**	10.4	10.4	**10.8**	10.5	11.0
Gly 117	-7.17	**-8.5**	-8.0	-8.2	**-8.3**	-8.0	-8.7
Val 120	-4.37	**-4.0**	-4.4	-4.2	**-4.7**	-4.4	-4.3
Gln 121	-6.83	**-8.1**	-5.8	-6.9	**-8.2**	-6.0	-8.2
Ala 122	-0.89	**-3.0**	-3.2	-3.0	**-2.7**	-3.2	-3.5
Ile 124	-5.73	**-8.1**	-5.7	-6.8	**-8.1**	-5.9	-8.1
Arg 125	1.26	**1.9**	0.7	1.3	**2.7**	0.7	1.4
Gly 126	-2.46	**-6.3**	-4.8	-5.4	**-6.5**	-4.8	-6.2
Cys 127	-3.28	**-3.1**	-3.4	-3.3	**-3.4**	-3.5	-3.6
Arg 128	-4.94	**-7.6**	-6.2	-6.8	**-7.9**	-6.2	-7.6
Leu 129	1.83	**9.5**	9.9	9.7	**9.7**	9.9	10.1
RMSD		**2.9**	2.5	2.6	**2.9**	2.5	2.8
rRMSD		-	2.5	2.6	-	2.5	2.8
urRMSD		**2.9**	-	-	**2.9**	-	-
N_{dev}		**22**	15	18	**21**	16	23
$N_{dev,s}$		**7**	8	8	**7**	7	8

Table S5. $^{13}C^{\alpha}$-$^{13}C'$ RDC values (97) in Hz for HEWL, as obtained from NMR measurements at 308 K and pH = 3.8, Table 4.4 of Ref.[16], and as calculated using three different sets of RDC restraints (RDC_{CAH59}, RDC_{NH101}, $RDC_{NCAH160}$) for the X-ray structure ***2VB1*** by applying the alignment-tensor method (*AT*: $\tau_D^{RDC} = 0$, $\tau_{AT}^{RDC} = 0$)[13] or the *HRS* ($K^{RDC,msy} = 0$) method[14]. $K^{RDC,mfv} = 100$ kJmol^{-1}Hz^{-2}, $\tau_{\theta}^{RDC,mfv} = 10$ ns, in $t^{mfv} = 30$ ns SD simulations of the magnetic-field vector. The RDC restraint set RDC_{CAH59} contains the RDC values D_k^0 given in the fourth column of Table 1. The RDC restraint set RDC_{NH101} contains the RDC values D_k^0 given in the second column of Table 2. The RDC restraint set $RDC_{NCAH160}$ is obtained by combining the sets of RDC restraints RDC_{CAH59} and RDC_{NH101}. The values for the RDCs that are *not* part of the (sub)set of RDC restraints applied, are in bold. *RMSD*: Root-mean-square difference (RMSD) between calculated D_k and D_k^0 RDC values calculated over all, *mfv*-restrained and unrestrained, RDCs. *rRMSD*: RMSD-values calculated over the particular (sub)set of *mfv*-restrained RDCs. *urRMSD*: RMSD-values calculated over the unrestrained RDCs. Deviations of RDC-values $D_{k_1k_2}$ (*AT*) or averaged $< D_{k_1k_2} >_{t^{mfv}}$ (*HRS*) from the D_k^0 values larger than 3 Hz are in red. N_{dev}: Number of such deviations. $N_{dev,s}$: Number of RDCs for which the calculated D_k and D_k^0 values have a different sign. These RDC values are in italics.

Residue	Experimental value (Hz)	*AT*			*HRS*		
		CAH59	*NH101*	*NCAH160*	*CAH59*	*NH101*	*NCAH160*
Lys 1	0.97	**1.5**	**1.0**	**1.2**	**1.4**	**1.1**	**1.5**
Val 2	-2.11	**-1.9**	**-1.7**	**-1.8**	**-2.0**	**-1.7**	**-1.9**
Phe 3	-0.50	**-0.3**	**-0.5**	**-0.4**	**-0.3**	**-0.5**	**-0.4**
Gly 4	1.95	**1.5**	**1.2**	**1.3**	**1.6**	**1.2**	**1.5**
Glu 7	-1.56	**-1.6**	**-1.1**	**-1.3**	**-1.4**	**-1.1**	**-1.5**
Leu 8	1.19	**1.2**	**0.9**	**1.0**	**1.2**	**0.9**	**1.2**
Ala 9	1.19	**1.3**	**1.2**	**1.2**	**1.2**	**1.2**	**1.3**
Ala 10	-1.30	**-1.3**	**-1.2**	**-1.2**	**-1.2**	**-1.2**	**-1.4**
Ala 11	0.13	**0.0**	**0.3**	**0.2**	**0.2**	**0.3**	**0.1**
Met 12	0.83	**0.7**	**0.5**	**0.6**	**0.6**	**0.5**	**0.7**
Lys 13	1.51	**1.4**	**1.2**	**1.3**	**1.4**	**1.2**	**1.4**
Arg 14	-1.94	**-2.4**	**-1.9**	**-2.1**	**-2.3**	**-1.9**	**-2.4**
His 15	1.01	**1.3**	**1.1**	**1.2**	**1.4**	**1.1**	**1.3**
Gly 16	0.55	**1.1**	**1.0**	**1.1**	**1.0**	**1.0**	**1.2**

Asp 18	1.71	1.3	1.2	1.3	1.4	1.2	1.4
Asn 19	-1.81	-1.7	-1.5	-1.6	-1.6	-1.5	-1.8
Tyr 23	-0.67	-1.1	-0.7	-0.9	-1.1	-0.7	-1.0
Ser24	1.34	1.3	0.9	1.1	1.3	1.0	1.3
Leu 25	0.76	1.5	1.4	1.5	1.5	1.4	1.5
Asn 27	-0.39	*0.1*	-0.3	-0.1	*0.1*	-0.3	*0.1*
Trp 28	1.41	1.2	1.2	1.2	1.3	1.2	1.2
Cys 30	-2.86	-2.8	-2.4	-2.6	-2.8	-2.5	-2.8
Ala 31	1.21	1.3	0.9	1.0	1.3	0.9	1.3
Ala 32	1.67	1.2	1.2	1.2	1.3	1.2	1.2
Lys 33	-0.74	-0.9	-0.7	-0.8	-1.1	-0.7	-0.9
Phe 34	-2.53	-2.4	-2.2	-2.3	-2.4	-2.2	-2.5
Ser 36	-1.04	-1.2	-0.8	-1.0	-1.1	-0.8	-1.2
Asn 37	0.81	0.8	0.7	0.7	0.6	0.8	0.8
Phe 38	0.83	0.6	0.2	0.4	0.6	0.2	0.6
Asn 39	-0.43	-0.5	-0.4	-0.4	-0.4	-0.4	-0.6
Thr 40	-2.42	-2.3	-2.1	-2.2	-2.4	-2.1	-2.4
Gln 41	0.14	0.3	0.4	0.4	0.2	0.4	0.4
Ala 42	-2.63	-2.4	-1.9	-2.1	-2.3	-1.9	-2.4
Asn 44	-1.67	-2.7	-2.2	-2.4	-2.6	-2.2	-2.7
Asn 46	-2.51	-2.6	-2.3	-2.4	-2.7	-2.3	-2.6
Thr 47	-0.95	-0.4	-0.1	-0.2	-0.2	-0.1	-0.3
Asp 48	1.48	1.3	0.9	1.1	1.3	1.0	1.3
Gly 49	-0.60	-0.6	-0.4	-0.5	-0.7	-0.4	-0.6
Ser 50	-1.22	-1.1	-0.7	-0.8	-0.9	-0.7	-1.1
Thr 51	-1.57	-1.2	-1.1	-1.2	-1.3	-1.1	-1.2
Tyr 53	-1.29	-2.6	-2.3	-2.4	-2.7	-2.3	-2.6
Leu 56	-2.63	-2.3	-1.8	-2.0	-2.4	-1.8	-2.3
Gln 57	-0.66	-0.4	-0.1	-0.2	-0.2	-0.1	-0.4
Asn 59	-1.71	-0.3	-0.3	-0.3	-0.2	-0.3	-0.4
Ser 60	-2.19	-1.4	-0.9	-1.1	-1.3	-0.9	-1.4
Arg 61	1.21	1.3	1.2	1.2	1.3	1.2	1.3
Cys 64	-2.38	-2.0	-1.9	-2.8	-2.1	-1.9	-2.1
Asn 65	0.92	1.4	1.3	1.3	1.5	1.3	1.5
Asp 66	-2.07	-2.3	-2.2	-2.3	-2.4	-2.2	-2.4
Gly 67	0.08	0.3	0.4	0.4	0.2	0.4	0.4
Arg 68	1.42	1.4	1.1	1.2	1.3	1.1	1.4
Gly 71	-2.77	-1.3	-0.9	-1.1	-1.4	-0.9	-1.2
Asn 74	0.44	0.3	0.1	0.2	0.2	0.1	0.2
Leu 75	-2.30	-1.8	-1.3	-1.5	-1.9	-1.3	-1.7
Cys 76	1.80	1.2	0.8	1.0	1.2	0.8	1.2
Asn 77	0.62	0.6	0.7	0.7	0.7	0.7	0.7
Pro 79	0.26	-0.6	-0.6	-0.6	-0.6	-0.6	-0.7
Cys 80	-0.23	-0.4	-0.1	-0.2	-0.3	-0.1	-0.3
Ser 81	-1.71	-1.8	-1.5	-1.7	-1.9	-1.6	-1.8
Ala 82	-0.80	-0.4	-0.6	-0.5	-0.4	-0.6	-0.5
Leu 83	1.12	0.8	0.9	0.8	0.9	0.9	0.8
Leu 84	-0.72	-0.6	-0.3	-0.4	-0.6	-0.3	-0.5
Ser 85	-2.60	-2.0	-1.8	-1.9	-2.0	-1.8	-2.1
Ile 88	2.38	0.7	0.8	0.8	0.9	0.8	0.7

Thr 89	-1.36	**-1.9**	**-1.4**	**-1.6**	**-1.9**	**-1.4**	**-1.8**
Ala 90	1.77	**1.5**	**1.1**	**1.3**	**1.4**	**1.2**	**1.5**
Ser 91	1.06	**0.5**	**0.2**	**0.3**	**0.5**	**0.2**	**0.5**
Val 92	1.08	**0.1**	**0.3**	**0.2**	**0.2**	**0.3**	**0.1**
Asn 93	-1.11	**-1.2**	**-0.8**	**-1.0**	**-1.3**	**-0.9**	**-1.2**
Cys 94	0.75	**1.0**	**0.6**	**0.8**	**1.0**	**0.6**	**1.0**
Ala 95	1.20	**1.3**	**1.1**	**1.2**	**1.4**	**1.1**	**1.4**
Lys 96	-1.79	**-1.9**	**-1.4**	**-1.6**	**-1.8**	**-1.4**	**-1.9**
Lys 97	0.79	**1.0**	**0.9**	**0.9**	**0.9**	**0.9**	**1.1**
Ile 98	-1.68	***0.7***	***0.3***	***0.5***	***0.7***	***0.3***	***0.7***
Val 99	1.81	**1.3**	**1.3**	**1.3**	**1.4**	**1.3**	**1.3**
Ser 100	-2.11	**-2.4**	**-1.9**	**-2.1**	**-2.4**	**-1.9**	**-2.4**
Gly 102	0.52	**1.7**	**1.6**	**1.6**	**1.7**	**1.6**	**1.7**
Gly 104	1.94	**1.4**	**1.3**	**1.3**	**1.5**	**1.3**	**1.4**
Met 105	0.41	**0.5**	**0.6**	**0.5**	**0.3**	**0.6**	**0.5**
Asn 106	-0.99	**-0.7**	**-0.9**	**-0.8**	**-0.7**	**-0.9**	**-0.8**
Ala 107	1.44	**1.2**	**1.2**	**1.2**	**1.3**	**1.2**	**1.2**
Ala 110	-1.26	**-0.8**	**-0.9**	**-0.9**	**-0.9**	**-1.0**	**-0.9**
Trp 111	1.12	**1.4**	**1.1**	**1.2**	**1.4**	**1.1**	**1.4**
Asn 113	-2.61	**-2.5**	**-2.0**	**-2.2**	**-2.5**	**-2.0**	**-2.5**
Arg 114	0.28	***-1.2***	***-1.0***	***-1.1***	***-1.4***	***-1.0***	***-1.2***
Cys 115	1.25	**1.4**	**1.1**	**1.2**	**1.5**	**1.1**	**1.4**
Lys 116	-0.35	**-0.0**	**-0.3**	**-0.2**	**-0.0**	**-0.3**	**-0.1**
Thr 118	-0.81	**-0.8**	**-0.5**	**-0.6**	**-0.9**	**-0.5**	**-0.7**
Val 120	-0.92	**-0.9**	**-0.6**	**-0.8**	**-1.0**	**-0.6**	**-0.8**
Gln 121	1.11	**0.8**	**0.9**	**0.9**	**1.0**	**0.9**	**0.9**
Ala 122	0.73	**0.9**	**0.4**	**0.6**	**0.9**	**0.4**	**0.8**
Trp 123	0.43	**0.7**	**0.7**	**0.7**	**0.6**	**0.7**	**0.8**
Ile 124	-3.36	**-2.9**	**-2.5**	**-2.7**	**-2.9**	**-2.5**	**-3.0**
Arg 125	0.76	**0.4**	**0.5**	**0.4**	**0.5**	**0.5**	**0.5**
Gly 126	0.95	**1.0**	**0.6**	**0.8**	**1.0**	**0.6**	**0.9**
Cys 127	0.43	**0.8**	**0.8**	**0.8**	**0.7**	**0.8**	**0.8**
Arg 128	-0.62	**-1.1**	**-0.7**	**-0.9**	**-0.9**	**-0.8**	**-1.1**
RMSD		**0.6**	**0.6**	**0.6**	**0.5**	**0.6**	**0.6**
rRMSD		-	-	-	-	-	-
urRMSD		**0.6**	**0.6**	**0.6**	**0.5**	**0.6**	**0.6**
N_{dev}		**0**	**0**	**0**	**0**	**0**	**0**
$N_{dev,s}$		**3**	**2**	**2**	**3**	**2**	**3**

Table S6. ^{13}C'-^{15}N RDC values (45) in Hz for HEWL, as obtained from NMR measurements at 308 K and pH = 3.8, Table 4.5 of Ref.[16], and as calculated using three different sets of RDC restraints (RDC_{CAH59}, RDC_{NH101}, $RDC_{NCAH160}$) for the X-ray structure ***2VB1*** by applying the alignment-tensor method (*AT*: $\tau_D^{RDC} = 0$, $\tau_{AT}^{RDC} = 0$)[13] or the *HRS* ($K^{RDC,msy} = 0$) method[14]. $K^{RDC,mfv} = 100$ kJmol^{-1}Hz^{-2}, $\tau_\theta^{RDC,mfv} = 10$ ns, in $t^{mfv} = 30$ ns SD simulations of the magnetic-field vector. The RDC restraint set RDC_{CAH59} contains the RDC values D_k^0 given in the fourth column of Table 1. The RDC restraint set RDC_{NH101} contains the RDC values D_k^0 given in the second column of Table 2. The RDC restraint set $RDC_{NCAH160}$ is obtained by combining the sets of RDC restraints RDC_{CAH59} and RDC_{NH101}. The values for the RDCs that are *not* part of the (sub)set of RDC restraints applied, are in bold. *RMSD*: Root-mean-square difference (RMSD) between calculated D_k and D_k^0 RDC values calculated over all, *mfv*-restrained and unrestrained, RDCs. *rRMSD*: RMSD-values calculated over the particular (sub)set of *mfv*-restrained RDCs. *urRMSD*: RMSD-values calculated over the unrestrained RDCs. Deviations of RDC-values $D_{k_1k_2}$ (*AT*) or averaged $< D_{k_1k_2} >_{t^{mfv}}$ (*HRS*) from the D_k^0 values larger than 3 Hz are in red. N_{dev}: Number of such deviations. $N_{dev,s}$: Number of RDCs for which the calculated D_k and the D_k^0 values have a different sign. These RDC values are in italics.

Residue	Experimental value (Hz)	*AT*			*HRS*		
		CAH59	*NH101*	*NCAH160*	*CAH59*	*NH101*	*NCAH160*
Gly 4	-0.55	**-0.5**	**-0.2**	**-0.3**	**-0.5**	**-0.2**	**-0.4**
Glu 7	0.20	**0.1**	***-0.0***	**0.0**	**0.2**	***-0.0***	**0.0**
Leu 8	0.67	**0.6**	**0.8**	**0.7**	**0.7**	**0.8**	**0.7**
Ala 9	-0.38	***0.0***	**-0.1**	**-0.0**	***0.0***	**-0.1**	**-0.0**
Ala 10	0.42	**0.4**	**0.5**	**0.5**	**0.5**	**0.5**	**0.5**
Ala 11	-0.95	**-0.8**	**-0.7**	**-0.7**	**-0.8**	**-0.7**	**-0.8**
Met 12	2.41	**1.8**	**1.5**	**1.7**	**1.8**	**1.6**	**1.8**
Lys 13	-0.78	**-0.8**	**-0.6**	**-0.7**	**-0.8**	**-0.7**	**-0.8**
Arg 14	1.07	**0.6**	**0.4**	**0.5**	**0.7**	**0.4**	**0.6**
Gly 16	-0.95	**-0.8**	**-0.8**	**-0.8**	**-0.9**	**-0.8**	**-0.9**
Trp 28	1.37	**1.6**	**1.3**	**1.5**	**1.6**	**1.3**	**1.6**
Ala 31	-0.23	**-0.2**	**-0.2**	**-0.2**	**-0.1**	**-0.2**	**-0.2**
Ala 32	0.38	**0.9**	**0.9**	**0.9**	**0.9**	**0.9**	**1.0**
Lys 33	-0.49	**-0.3**	**-0.4**	**-0.3**	**-0.4**	**-0.4**	**-0.4**

Asn 37	-1.12	**-0.9**	**-0.8**	**-0.9**	**-0.9**	**-0.8**	**-0.9**
Phe 38	1.72	**1.2**	**1.0**	**1.1**	**1.3**	**1.0**	**1.2**
Asn 39	-0.78	**-0.8**	**-0.7**	**-0.7**	**-0.7**	**-0.7**	**-0.8**
Thr 40	-1.41	**-0.9**	**-0.8**	**-0.8**	**-0.9**	**-0.8**	**-0.9**
Gln 41	-0.26	**-0.5**	**-0.2**	**-0.4**	**-0.5**	**-0.2**	**-0.5**
Thr 47	-1.13	**-0.5**	**-0.4**	**-0.5**	**-0.5**	**-0.4**	**-0.5**
Arg 61	1.21	**0.7**	**0.8**	**0.8**	**0.7**	**0.8**	**0.8**
Arg 68	-0.64	**-0.6**	**-0.5**	**-0.5**	**-0.6**	**-0.5**	**-0.6**
Leu 75	-0.57	**-0.4**	**-0.5**	**-0.4**	**-0.5**	**-0.5**	**-0.4**
Cys 76	1.35	**1.6**	**1.4**	**1.5**	**1.6**	**1.5**	**1.7**
Asn 77	-0.81	**-0.9**	**-0.9**	**-0.9**	**-0.9**	**-0.9**	**-0.9**
Ser 81	-1.23	**-0.9**	**-0.8**	**-0.9**	**-1.0**	**-0.8**	**-0.9**
Leu 83	-0.89	**-0.7**	**-0.5**	**-0.6**	**-0.7**	**-0.5**	**-0.7**
Leu 84	-0.63	**-0.7**	**-0.7**	**-0.7**	**-0.7**	**-0.7**	**-0.7**
Ala 90	-0.78	**-0.2**	**-0.3**	**-0.2**	**-0.3**	**-0.3**	**-0.2**
Asn 93	-0.28	**-0.4**	**-0.3**	**-0.3**	**-0.3**	**-0.3**	**-0.4**
Ala 95	-0.40	**-0.5**	**-0.5**	**-0.5**	**-0.4**	**-0.5**	**-0.6**
Lys 96	1.14	**0.8**	**0.9**	**0.9**	**0.8**	**0.9**	**0.9**
Lys 97	-1.40	**-0.9**	**-0.8**	**-0.9**	**-0.9**	**-0.8**	**-0.9**
Gly 104	0.60	**1.7**	**1.4**	**1.5**	**1.7**	**1.4**	**1.7**
Met 105	-0.05	**-0.2**	**-0.3**	**-0.2**	**-0.2**	**-0.3**	**-0.3**
Ala 110	-1.01	**-0.9**	**-0.9**	**-0.9**	**-0.9**	**-0.9**	**-0.9**
Trp 111	1.13	**1.2**	**0.9**	**1.0**	**1.2**	**0.9**	**1.2**
Asn 113	-0.28	**-1.1**	**-1.0**	**-1.1**	**-1.0**	**-1.1**	**-1.2**
Arg 114	-1.14	**-0.6**	**-0.6**	**-0.6**	**-0.6**	**-0.6**	**-0.6**
Cys 115	0.97	**1.1**	**0.8**	**0.9**	**1.0**	**0.8**	**1.1**
Gln 121	-0.78	**-0.9**	**-0.8**	**-0.8**	**-0.9**	**-0.8**	**-0.9**
Ala 122	1.68	**1.3**	**1.1**	**1.2**	**1.2**	**1.1**	**1.3**
Arg 125	1.26	**1.0**	**0.8**	**0.9**	**1.1**	**0.8**	**1.0**
Gly 126	0.52	**1.0**	**0.8**	**0.9**	**1.0**	**0.8**	**1.1**
Cys 127	-0.67	**-0.9**	**-0.7**	**-0.7**	**-0.8**	**-0.7**	**-0.9**
RMSD		**0.4**	**0.4**	**0.4**	**0.4**	**0.4**	**0.4**
rRMSD		-	-	-	-	-	-
urRMSD		**0.4**	**0.4**	**0.4**	**0.4**	**0.4**	**0.4**
N_{dev}		**0**	**0**	**0**	**0**	**0**	**0**
$N_{dev,s}$		**1**	**1**	**0**	**1**	**1**	**0**

Table S7. $^{13}C^{\alpha}$-$^{1}H^{\alpha}$ RDC values (38, 39) in Hz for HEWL, as obtained from NMR measurements at 308 K and pH = 3.8 using two different pulse sequences as given in Tables 4.2 and 4.3 of Ref.[16], and as calculated using three different sets of RDC restraints (RDC_{CAH59}, RDC_{NH101}, $RDC_{NCAH160}$) for the X-ray structure ***1IEE*** by applying the alignment-tensor method (*AT*: $\tau_D^{RDC} = 0$, $\tau_{AT}^{RDC} = 0$)[13] or the *HRS* ($K^{RDC,msy} = 0$) method[14], $K^{RDC,mfv} = 100$ kJmol^{-1}Hz^{-2}, $\tau_\theta^{RDC,mfv} = 10$ ns, in $t^{mfv} = 30$ ns SD simulations of the magnetic-field vector. The (RDC restraint) set RDC_{CAH59} contains the RDC values D_k^0 given in the fourth column. The RDC restraint set RDC_{NH101} contains the RDC values D_k^0 given in the second column of Table 2. The RDC restraint set $RDC_{NCAH160}$ is obtained by combining the sets of RDC restraints RDC_{CAH59} and RDC_{NH101}. In case two experimental values are available, the D_k^0 RDC values used in the calculations are the average of the two experimental values. The values for the RDCs that are *not* part of the (sub)set of RDC restraints applied, are in bold. *RMSD*: Root-mean-square difference (RMSD) between calculated D_k and D_k^0 RDC values calculated over all, *mfv*-restrained and unrestrained, RDCs. *rRMSD*: RMSD-values calculated over the particular (sub)set of *mfv*-restrained RDCs. *urRMSD*: RMSD-values calculated over the unrestrained RDCs. Deviations of RDC values $D_{k_1k_2}$ (*AT*) or averaged $< D_{k_1k_2} >_{t^{mfv}}$ (*HRS*) from the D_k^0 values larger than 3 Hz are in red. N_{dev}: Number of such deviations. $N_{dev,s}$: Number of RDCs for which the calculated D_k and the D_k^0 values have a different sign. These RDC values are in italics.

Residue	Experimental value (Hz)			*AT*			*HRS*		
	Table 4.2[16]	Table 4.3[16]	D_k^0	*CAH59*	*NH101*	*NCAH160*	*CAH59*	*NH101*	*NCAH160*
Val 2	-5.30	-	-5.30	-9.4	**-5.1**	-6.7	-9.0	**-5.5**	-8.0
Phe 3	-	8.85	8.85	7.6	**8.0**	7.9	7.4	**7.4**	7.1
Cys 6	-	15.30	15.30	13.1	**12.2**	12.7	13.2	**12.3**	12.9
Glu 7	-	-6.52	-6.52	-1.2	**-0.5**	-1.0	-1.6	**-0.6**	-1.8
Leu 8	-	3.17	3.17	3.1	**1.2**	2.1	3.1	**0.7**	2.7
Met 12	-20.12	-22.20	-21.16	-23.7	**-22.1**	-22.9	-23.6	**-22.3**	-23.2

Lys 13	10.26	10.33	10.30	6.4	**7.6**	7.3	6.6	**7.3**	6.8
His 15	15.60	-	15.60	15.7	**14.7**	15.3	16.0	**14.1**	15.8
Leu 17	-	-10.66	-10.66	-15.0	**-9.5**	-11.6	-13.9	**-9.9**	-12.0
Asp 18	-	19.61	19.61	16.1	**15.3**	15.7	16.4	**15.4**	16.5
Tyr 23	-	13.81	13.81	13.2	**10.5**	11.7	13.1	**9.9**	12.2
Leu 25	-18.58	-	-18.58	-19.0	**-18.0**	-18.4	-18.8	**-18.2**	-18.5
Trp 28	-28.74	-28.51	-28.62	-28.8	**-24.2**	-26.3	-28.9	**-24.4**	-28.1
Val 29	5.17	-	5.17	7.5	**3.0**	4.8	6.8	**3.0**	5.4
Cys 30	9.83	8.84	9.34	4.2	**7.2**	6.3	5.4	**7.2**	6.9
Lys 33	13.60	-	13.60	12.6	**11.5**	12.1	12.4	**11.3**	12.0
Phe 34	-	15.36	15.36	12.4	**13.5**	13.3	13.3	**13.8**	14.3
Glu 35	-	-5.65	-5.65	-7.3	**-5.0**	-6.1	-7.6	**-5.4**	-7.5
Asn 39	-6.88	-4.05	-5.46	-8.5	**-4.9**	-6.4	-8.4	**-5.5**	-7.9
Thr 40	15.69	15.81	15.75	15.4	**13.0**	14.1	15.2	**13.3**	14.5
Ala 42	-	18.93	18.93	17.3	**15.2**	16.1	17.4	**16.0**	17.3
Asn 44	21.03	-	21.03	17.1	**14.5**	15.6	17.0	**15.4**	16.8
Thr 47	-	12.40	12.40	16.6	**13.4**	14.7	16.4	**14.2**	15.8
Asp 48	-17.97	-18.29	-18.13	-11.5	**-9.5**	-10.6	-11.9	**-9.6**	-12.0
Thr 51	16.96	-	16.96	14.7	**14.6**	14.9	15.4	**14.1**	15.8
Asp 52	-	-5.43	-5.43	*12.9*	***14.0***	*13.8*	*13.9*	***14.2***	*15.0*
Leu 56	-0.83	-	-0.83	-1.2	**-0.9**	-0.8	-0.6	**-1.5**	-0.2
Gln 57	6.72	-	6.72	12.3	**7.2**	9.3	11.5	**7.8**	10.0
Asn 59	17.60	-	17.60	17.2	**15.2**	16.1	17.3	**16.0**	17.2
Trp 62	-19.22	-22.42	-20.82	-22.9	**-17.7**	-19.7	-22.0	**-18.2**	-20.4
Trp 63	17.30	-	17.30	14.5	**14.9**	15.0	15.3	**15.3**	16.1
Cys 64	18.24	-	18.24	16.4	**15.8**	16.1	16.8	**15.7**	16.9
Asn 65	0.76	-	0.76	2.0	**3.6**	3.0	1.9	**3.0**	1.9
Pro 70	5.72	-	5.72	6.2	**4.8**	5.6	6.5	**4.2**	6.2
Ile 78	-	17.81	17.81	15.1	**13.7**	14.5	15.3	**13.0**	15.0
Ser 81	14.90	-	14.90	12.4	**12.9**	13.0	13.3	**12.5**	14.0
Leu 84	-	5.15	5.15	7.5	**5.0**	6.1	7.4	**4.4**	6.7
Ser 85	-	13.38	13.38	14.5	**9.9**	11.7	13.8	**10.6**	12.6
Ile 88	-14.50	-15.25	-14.88	-12.0	**-13.6**	-13.4	-13.0	**-12.8**	-13.8
Thr 89	-0.08	-3.00	-1.54	-4.6	**-9.1**	-7.5	-5.7	**-8.4**	-7.2
Ala 90	-	17.24	17.24	15.7	**15.9**	16.1	16.5	**16.2**	17.3
Val 92	14.69	-	14.69	15.1	**13.5**	14.3	15.2	**12.8**	14.8
Asn 93	-	10.98	10.98	1.8	***-2.5***	*-1.1*	0.8	***-1.4***	*-0.6*
Lys 96	-1.72	-	-1.72	-4.4	**-6.8**	-5.8	-4.7	**-7.0**	-5.4
Ile 98	-26.52	-27.59	-27.06	-30.8	**-25.6**	-27.8	-30.5	**-25.9**	-29.3
Val 99	-	18.49	18.49	11.3	**11.0**	11.1	11.2	**10.6**	11.0
Ser 100	-8.22	-	-8.22	-5.9	**-9.9**	-8.5	-6.8	**-9.5**	-8.2
Trp 108	-10.09	-	-10.09	-7.7	**-11.8**	-10.4	-8.8	**-11.1**	-10.2
Val 109	14.56	19.08	16.82	15.6	**13.0**	14.2	15.3	**13.3**	14.7
Trp 111	-22.31	-22.56	-22.44	-20.9	**-14.9**	-17.2	-19.9	**-15.3**	-18.2
Arg 112	-	12.73	12.73	0.3	***-3.4***	*-2.2*	*-0.7*	***-2.3***	*-1.9*
Cys 115	-	-21.39	-21.39	-15.8	**-10.6**	-12.2	-14.6	**-11.0**	-12.8
Lys 116	8.87	3.30	6.08	10.5	**9.7**	10.0	10.4	**9.8**	10.2
Thr 118	12.69	-	12.69	11.3	**9.1**	10.1	11.3	**8.4**	10.7
Asp 119	17.09	15.66	16.37	17.1	**14.6**	15.7	17.1	**15.5**	16.8
Val 120	13.71	13.50	13.65	15.1	**12.0**	13.2	14.8	**13.0**	14.3

Gln 121	12.41	9.50	10.95	6.3	**6.1**	6.4	6.9	**5.4**	7.2
Ala 122	-	-30.56	-30.56	-31.4	**-28.3**	-29.9	-31.7	**-28.3**	-31.3
Cys 127	14.73	16.44	15.58	14.7	**13.2**	14.0	14.7	**13.6**	14.5
RMSD				4.3	**5.2**	4.6	4.3	**5.0**	4.5
rRMSD				4.3	-	4.6	4.3	-	4.5
urRMSD				-	**5.2**	-	-	**5.0**	-
N_{dev}				20	**22**	16	19	**19**	17
$N_{dev,s}$				1	**3**	3	2	**3**	3

Table S8. ^{15}N-$^{1}H^{N}$ RDC values (101) in Hz for HEWL, as obtained from NMR measurements at 308 K and pH = 3.8, Table 4.1 of Ref.[16], and as calculated using three different sets of RDC restraints (*RDC*$_{CAH59}$, *RDC*$_{NH101}$, *RDC*$_{NCAH160}$) for the X-ray structure ***1IEE*** by applying the alignment-tensor method (*AT*: $\tau_D^{RDC} = 0$, $\tau_{AT}^{RDC} = 0$)[13] or the *HRS* ($K^{RDC,msy} = 0$) method[14], $K^{RDC,mfv}$ = 100 kJmol^{-1}Hz^{-2}, $\tau_\theta^{RDC,mfv}$ = 10 ns, in t^{mfv} = 30 ns SD simulations of the magnetic-field vector. The RDC restraint set *RDC*$_{CAH59}$ contains the RDC values D_k^0 given in the fourth column of Table 1. The RDC restraint set *RDC*$_{NH101}$ contains the RDC values D_k^0 given in the second column. The RDC restraint set *RDC*$_{NCAH160}$ is obtained by combining the sets of RDC restraints *RDC*$_{CAH59}$ and *RDC*$_{NH101}$. The values for the RDCs that are *not* part of the (sub)set of RDC restraints applied, are in bold. *RMSD*: Root-mean-square difference (RMSD) between calculated D_k and D_k^0 RDC values calculated over all, *mfv*-restrained and unrestrained, RDCs. *rRMSD*: RMSD-values calculated over the particular (sub)set of *mfv*-restrained RDCs. *urRMSD*: RMSD-values calculated over the unrestrained RDCs. Deviations of RDC values $D_{k_1k_2}$ (*AT*) or averaged $< D_{k_1k_2} >_{t^{mfv}}$ (*HRS*) from the D_k^0 values larger than 3 Hz are in red. N_{dev}: Number of such deviations. $N_{dev,s}$: Number of RDCs for which the calculated D_k and the D_k^0 values have a different sign. These RDC values are in italics.

Residue	Experimental value (Hz), target D_k^0	*AT*			*HRS*		
		CAH59	*NH101*	*NCAH160*	*CAH59*	*NH101*	*NCAH160*
Val 2	-1.78	**-3.9**	-4.4	-4.3	**-4.0**	-4.4	-4.2
Phe 3	7.52	**11.9**	8.8	10.0	**11.6**	9.0	10.1
Gly 4	-6.92	**-6.5**	-6.2	-6.3	**-6.4**	-5.8	-6.2
Arg 5	-6.47	**-2.3**	-3.8	-3.3	**-3.0**	-3.7	-3.8
Cys 6	-3.68	**-1.5**	-3.2	-2.7	**-2.2**	-3.1	-3.0
Glu 7	-8.56	**-8.2**	-8.3	-8.4	**-8.6**	-8.5	-9.1
Leu 8	-7.85	**-7.3**	-7.6	-7.6	**-7.8**	-7.5	-8.2
Ala 9	-4.27	**-3.1**	-4.4	-4.1	**-3.8**	-4.3	-4.5
Ala 11	-8.64	**-8.8**	-8.6	-8.8	**-9.1**	-8.8	-9.4
Lys 13	-3.36	**-2.3**	-3.9	-3.4	**-3.0**	-3.9	-3.8
Arg 14	-7.36	**-8.0**	-8.0	-8.1	**-8.3**	-8.2	-8.7
Gly 16	12.38	**12.7**	9.6	10.8	**12.2**	9.8	11.4

Leu 17	-4.59	**-4.1**	-4.7	-4.6	**-4.3**	-4.7	-4.5
Asn 19	-5.96	**-8.7**	-8.6	-8.7	**-9.1**	-8.7	-9.4
Tyr 20	0.61	***-6.5***	*-6.6*	*-6.7*	***-6.8***	*-6.8*	*-7.0*
Arg 21	-6.77	**-8.6**	-8.3	-8.5	**-8.8**	-8.1	-8.9
Gly 22	-2.70	**-4.5**	-4.6	-4.6	**-4.4**	-4.3	-4.3
Tyr 23	0.23	***-1.2***	*-0.6*	*-0.9*	***-1.3***	*-0.3*	*-1.2*
Ser24	-6.59	**-7.8**	-6.7	-7.3	**-7.7**	-6.4	-7.3
Gly 26	4.19	**0.9**	3.3	2.5	**1.5**	1.7	2.3
Asn 27	-5.76	**-5.2**	-3.4	-4.0	**-4.9**	-4.0	-4.5
Trp 28	5.29	**2.5**	3.6	3.3	**3.0**	3.1	3.4
Val 29	3.56	**2.0**	4.0	3.4	**2.6**	3.5	3.3
Ala 31	-3.83	**-4.8**	-3.3	-3.8	**-4.5**	-3.8	-4.1
Ala 32	4.34	**1.6**	2.9	2.6	**2.1**	2.4	2.6
Lys 33	0.35	***-2.4***	0.1	*-0.8*	***-1.8***	*-0.5*	*-1.1*
Phe 34	-4.22	**-7.8**	-5.9	-6.6	**-7.6**	-6.5	-7.2
Glu 35	3.73	**3.1**	3.8	3.7	**3.6**	3.5	3.9
Ser 36	7.51	**9.0**	10.2	9.9	**9.6**	9.8	10.1
Asn 37	-7.43	**-7.6**	-6.5	-7.0	**-7.5**	-6.5	-7.1
Phe 38	-0.76	***0.3***	-1.4	-0.8	**-0.0**	-1.2	-0.5
Asn 39	-2.69	**-2.5**	-3.0	-2.8	**-2.5**	-2.7	-2.4
Thr 40	6.48	**10.1**	7.4	8.5	**9.9**	7.6	9.4
Gln 41	6.78	**9.2**	6.6	7.6	**8.6**	6.8	7.7
Ala 42	10.72	**9.0**	9.9	9.8	**9.6**	9.4	10.0
Thr 43	-6.94	**-7.5**	-7.8	-7.8	**-7.9**	-8.0	-8.4
Asn 44	-8.88	**-7.6**	-8.0	-7.9	**-8.1**	-8.1	-8.6
Arg 45	-7.32	**-7.9**	-7.9	-8.0	**-8.2**	-8.1	-8.6
Asn 46	-4.82	**-5.3**	-5.2	-5.4	**-5.7**	-4.9	-5.9
Thr 47	4.71	**5.9**	7.5	7.0	**6.3**	7.3	6.9
Asp 48	14.01	**13.1**	12.9	13.2	**13.5**	12.7	13.7
Gly 49	1.82	**5.6**	3.9	4.5	**5.0**	4.0	4.3
Thr 51	-7.92	**-8.0**	-8.2	-8.2	**-8.4**	-8.1	-8.8
Gly 54	-6.58	**-7.9**	-6.3	-6.9	**-7.7**	-6.8	-7.5
Ile 55	-7.54	**-8.2**	-7.5	-7.9	**-8.2**	-7.2	-8.1
Leu 56	-0.14	**-1.1**	-0.3	-0.7	**-1.2**	-0.1	-1.1
Gln 57	-3.19	**-3.2**	-3.8	-3.7	**-3.3**	-3.6	-3.3
Ile 58	-3.83	**-3.3**	-3.8	-3.7	**-3.3**	-3.6	-3.3
Asn 59	-3.85	**-7.7**	-5.5	-6.4	**-7.4**	-6.0	-6.9
Ser 60	-7.82	**-8.7**	-8.4	-8.6	**-8.9**	-8.4	-9.0
Trp 63	-8.15	**-9.0**	-8.5	-8.7	**-9.2**	-8.7	-9.3
Asn 65	1.18	**0.7**	0.0	0.3	**0.8**	0.2	0.9
Asp 66	-0.81	**-0.3**	-1.4	-1.0	**-0.3**	-1.2	-0.5
Gly 67	-7.13	**-8.6**	-6.8	-7.5	**-8.4**	-7.3	-8.1
Arg 68	13.60	**12.1**	12.5	12.6	**12.6**	12.3	12.9
Thr 69	-3.43	**-8.4**	-8.0	-8.2	**-8.6**	-7.8	-8.5
Gly 71	2.44	***-0.4***	1.2	0.5	***-0.2***	1.3	0.3
Arg 73	-1.26	***2.9***	*2.2*	*2.4*	***2.5***	*2.5*	*2.1*
Asn 74	-7.40	**-8.7**	-8.5	-8.7	**-9.0**	-8.4	-9.1
Cys 76	-4.97	**-7.0**	-6.3	-6.5	**-6.9**	-6.5	-6.9
Ile 78	-1.37	**-1.0**	-1.6	-1.3	**-0.9**	-1.4	-0.8
Cys 80	16.06	**16.3**	14.7	15.5	**16.4**	14.7	16.3

Ser 81	14.37	**13.3**	12.1	12.6	**13.2**	12.2	12.9
Ala 82	7.74	**12.7**	9.6	10.8	**12.2**	9.8	11.3
Leu 83	15.17	**14.5**	13.4	14.0	**14.8**	13.3	14.8
Leu 84	5.43	**1.9**	2.3	2.0	**1.7**	2.5	1.8
Ser 86	-4.45	**-5.7**	-3.3	-4.3	**-5.3**	-3.3	-4.6
Asp 87	12.03	**5.2**	6.8	6.2	**5.6**	6.7	6.1
Thr 89	-2.93	**-4.8**	-4.9	-4.9	**-4.8**	-4.7	-4.7
Ser 91	-0.10	**-1.7**	-2.9	-2.5	**-2.0**	-2.8	-2.3
Val 92	-4.32	**-3.6**	-4.0	-3.9	**-3.6**	-3.8	-3.6
Asn 93	-6.06	**-7.5**	-6.5	-7.0	**-7.4**	-6.5	-7.1
Cys 94	-5.79	**-5.2**	-5.6	-5.6	**-5.4**	-5.6	-5.7
Ala 95	-2.42	**-1.9**	-2.9	-2.6	**-2.0**	-2.7	-2.2
Lys 96	-6.08	**-7.0**	-6.2	-6.6	**-6.9**	-6.0	-6.5
Lys 97	-7.62	**-8.0**	-6.5	-7.2	**-7.8**	-6.6	-7.3
Ile 98	-4.26	**-3.7**	-4.2	-4.1	**-3.8**	-4.1	-3.9
Ser 100	-4.10	**-6.4**	-3.9	-5.0	**-6.0**	-4.0	-5.3
Gly 102	8.96	**11.8**	8.7	9.9	**11.4**	8.9	10.6
Asn 103	-7.26	**-5.3**	-2.8	-3.8	**-4.9**	-2.9	-4.1
Gly 104	-4.67	**-1.9**	-3.0	-2.7	**-2.5**	-2.7	-3.1
Met 105	9.39	**4.3**	6.4	5.7	**4.9**	6.0	5.6
Asn 106	-7.82	**-8.8**	-7.3	-8.0	**-8.8**	-7.8	-8.6
Ala 107	-1.63	**-4.2**	-3.1	-3.5	**-4.0**	-3.5	-3.7
Trp 108	3.79	**2.6**	4.4	3.9	**3.2**	3.8	3.8
Val 109	7.54	***-0.1***	2.2	1.3	**0.3**	2.0	1.0
Trp 111	9.71	**5.0**	4.9	4.9	**4.9**	5.2	4.7
Arg 112	8.40	**7.9**	8.4	8.2	**8.0**	8.4	8.2
Asn 113	1.87	***-1.4***	0.7	*-0.2*	***-1.1***	0.7	*-0.4*
Cys 115	14.32	**14.6**	11.8	13.0	**14.3**	12.0	13.5
Lys 116	12.40	**10.5**	11.3	11.2	**11.0**	11.0	11.5
Gly 117	-7.17	**-8.3**	-8.0	-8.2	**-8.5**	-7.8	-8.5
Val 120	-4.37	**-5.2**	-5.6	-5.6	**-5.4**	-5.7	-5.7
Gln 121	-6.83	**-8.0**	-6.2	-7.0	**-7.7**	-6.2	-7.2
Ala 122	-0.89	**-1.9**	-2.6	-2.4	**-1.9**	-2.3	-1.8
Ile 124	-5.73	**-7.9**	-5.9	-6.7	**-7.6**	-5.9	-7.0
Arg 125	1.26	**3.4**	2.7	3.1	**3.6**	2.7	3.7
Gly 126	-2.46	**-3.5**	-0.8	-1.8	**-3.0**	-1.3	-2.2
Cys 127	-3.28	**-1.5**	-2.4	-2.1	**-1.6**	-2.1	-1.6
Arg 128	-4.94	**-7.6**	-5.2	-6.1	**-7.2**	-5.7	-6.6
Leu 129	1.83	**2.4**	4.4	3.7	**2.8**	4.3	3.5
RMSD		**2.4**	1.9	2.0	**2.2**	1.9	2.1
rRMSD		-	1.9	2.0	-	1.9	2.1
urRMSD		**2.4**	-	-	**2.2**	-	-
N_{dev}		**17**	8	11	**16**	8	12
$N_{dev,s}$		**7**	3	5	**6**	4	5

Table S9. $^{13}C^{\alpha}$-$^{13}C'$ RDC values (97) in Hz for HEWL, as obtained from NMR measurements at 308 K and pH = 3.8, Table 4.4 of Ref.[16], and as calculated using three different sets of RDC restraints (RDC_{CAH59}, RDC_{NH101}, $RDC_{NCAH160}$) for the X-ray structure ***1IEE*** by applying the alignment-tensor method (*AT*: $\tau_D^{RDC} = 0$, $\tau_{AT}^{RDC} = 0$)[13] or the *HRS* ($K^{RDC,msy} = 0$) method[14]. $K^{RDC,mfv} = 100$ kJmol^{-1}Hz^{-2}, $\tau_\theta^{RDC,mfv} = 10$ ns, in $t^{mfv} = 30$ ns SD simulations of the magnetic-field vector. The RDC restraint set RDC_{CAH59} contains the RDC values D_k^0 given in the fourth column of Table 1. The RDC restraint set RDC_{NH101} contains the RDC values D_k^0 given in the second column of Table 2. The RDC restraint set $RDC_{NCAH160}$ is obtained by combining the sets of RDC restraints RDC_{CAH59} and RDC_{NH101}. The values for the RDCs that are *not* part of the (sub)set of RDC restraints applied, are in bold. *RMSD*: Root-mean-square difference (RMSD) between calculated D_k and D_k^0 RDC values calculated over all, *mfv*-restrained and unrestrained, RDCs. *rRMSD*: RMSD-values calculated over the particular (sub)set of *mfv*-restrained RDCs. *urRMSD*: RMSD-values calculated over the unrestrained RDCs. Deviations of RDC-values $D_{k_1k_2}$ (*AT*) or averaged $< D_{k_1k_2} >_{t^{mfv}}$ (*HRS*) from the D_k^0 values larger than 3 Hz are in red. N_{dev}: Number of such deviations. $N_{dev,s}$: Number of RDCs for which the calculated D_k and D_k^0 values have a different sign. These RDC values are in italics.

Residue	Experimental value (Hz)	*AT*			*HRS*		
		CAH59	*NH101*	*NCAH160*	*CAH59*	*NH101*	*NCAH160*
Lys 1	0.97	**1.2**	**1.0**	**1.1**	**1.2**	**0.9**	**1.1**
Val 2	-2.11	**-1.5**	**-1.6**	**-1.6**	**-1.6**	**-1.5**	**-1.7**
Phe 3	-0.50	**-0.6**	**-0.5**	**-0.5**	**-0.5**	**-0.6**	**-0.5**
Gly 4	1.95	**1.4**	**1.2**	**1.3**	**1.4**	**1.2**	**1.3**
Glu 7	-1.56	**-1.3**	**-0.8**	**-1.0**	**-1.2**	**-0.8**	**-1.0**
Leu 8	1.19	**1.4**	**0.9**	**1.1**	**1.3**	**1.0**	**1.2**
Ala 9	1.19	**1.3**	**1.2**	**1.3**	**1.3**	**1.2**	**1.3**
Ala 10	-1.30	**-1.3**	**-1.1**	**-1.2**	**-1.2**	**-1.1**	**-1.1**
Ala 11	0.13	***-0.0***	**0.3**	**0.2**	**0.0**	**0.2**	**0.1**
Met 12	0.83	**0.8**	**0.4**	**0.6**	**0.7**	**0.5**	**0.6**
Lys 13	1.51	**1.5**	**1.4**	**1.5**	**1.5**	**1.4**	**1.5**
Arg 14	-1.94	**-2.2**	**-1.7**	**-1.9**	**-2.1**	**-1.8**	**-2.0**
His 15	1.01	**1.1**	**1.1**	**1.1**	**1.1**	**1.1**	**1.1**
Gly 16	0.55	**0.8**	**0.8**	**0.8**	**0.8**	**0.8**	**0.8**

Asp 18	1.71	**1.2**	**1.2**	**1.2**	**1.3**	**1.2**	**1.3**
Asn 19	-1.81	**-1.7**	**-1.3**	**-1.4**	**-1.6**	**-1.4**	**-1.5**
Tyr 23	-0.67	**-1.3**	**-0.8**	**-1.0**	**-1.2**	**-0.9**	**-1.2**
Ser24	1.34	**1.5**	**1.1**	**1.2**	**1.4**	**1.1**	**1.3**
Leu 25	0.76	**1.6**	**1.5**	**1.5**	**1.6**	**1.5**	**1.6**
Asn 27	-0.39	***0.2***	**-0.3**	**-0.1**	***0.1***	**-0.2**	**-0.1**
Trp 28	1.41	**1.2**	**1.2**	**1.2**	**1.2**	**1.2**	**1.3**
Cys 30	-2.86	**-2.6**	**-2.5**	**-2.6**	**-2.7**	**-2.5**	**-2.7**
Ala 31	1.21	**1.4**	**1.0**	**1.1**	**1.3**	**1.0**	**1.2**
Ala 32	1.67	**1.2**	**1.3**	**1.3**	**1.3**	**1.3**	**1.4**
Lys 33	-0.74	**-1.1**	**-1.0**	**-1.0**	**-1.1**	**-1.0**	**-1.2**
Phe 34	-2.53	**-2.2**	**-2.1**	**-2.2**	**-2.2**	**-2.1**	**-2.2**
Ser 36	-1.04	**-1.3**	**-0.8**	**-0.9**	**-1.2**	**-0.8**	**-1.0**
Asn 37	0.81	**0.6**	**0.7**	**0.7**	**0.6**	**0.6**	**0.6**
Phe 38	0.83	**0.8**	**0.4**	**0.6**	**0.7**	**0.4**	**0.6**
Asn 39	-0.43	**-0.8**	**-0.5**	**-0.6**	**-0.7**	**-0.5**	**-0.5**
Thr 40	-2.42	**-2.1**	**-2.2**	**-2.2**	**-2.2**	**-2.1**	**-2.2**
Gln 41	0.14	**0.1**	**0.3**	**0.2**	**0.1**	**0.2**	**0.1**
Ala 42	-2.63	**-2.3**	**-1.7**	**-1.9**	**-2.2**	**-1.8**	**-2.0**
Asn 44	-1.67	**-2.2**	**-1.6**	**-1.8**	**-2.1**	**-1.7**	**-2.0**
Asn 46	-2.51	**-1.8**	**-1.7**	**-1.8**	**-1.9**	**-1.6**	**-1.9**
Thr 47	-0.95	**-1.3**	**-1.0**	**-1.1**	**-1.2**	**-1.0**	**-1.1**
Asp 48	1.48	**1.1**	**0.8**	**0.9**	**1.1**	**0.7**	**1.0**
Gly 49	-0.60	***0.2***	***0.2***	***0.2***	***0.2***	***0.2***	***0.1***
Ser 50	-1.22	**-1.3**	**-0.8**	**-1.0**	**-1.2**	**-0.8**	**-1.0**
Thr 51	-1.57	**-0.9**	**-1.1**	**-1.1**	**-1.0**	**-1.0**	**-1.1**
Tyr 53	-1.29	**-2.3**	**-2.3**	**-2.3**	**-2.4**	**-2.2**	**-2.4**
Leu 56	-2.63	**-2.6**	**-2.2**	**-2.4**	**-2.6**	**-2.2**	**-2.6**
Gln 57	-0.66	**-0.7**	**-0.3**	**-0.4**	**-0.6**	**-0.3**	**-0.4**
Asn 59	-1.71	**-0.6**	**-0.5**	**-0.5**	**-0.5**	**-0.6**	**-0.5**
Ser 60	-2.19	**-1.8**	**-1.2**	**-1.5**	**-1.7**	**-1.3**	**-1.6**
Arg 61	1.21	**1.6**	**1.4**	**1.4**	**1.6**	**1.4**	**1.6**
Cys 64	-2.38	**-1.9**	**-2.0**	**-2.0**	**-2.0**	**-2.0**	**-2.1**
Asn 65	0.92	**1.3**	**1.3**	**1.3**	**1.4**	**1.3**	**1.4**
Asp 66	-2.07	**-2.0**	**-2.1**	**-2.1**	**-2.1**	**-2.1**	**-2.2**
Gly 67	0.08	**0.5**	**0.5**	**0.5**	**0.5**	**0.5**	**0.4**
Arg 68	1.42	**1.3**	**1.0**	**1.1**	**1.3**	**1.0**	**1.2**
Gly 71	-2.77	**-1.9**	**-1.7**	**-1.8**	**-2.0**	**-1.7**	**-2.0**
Asn 74	0.44	**0.7**	**0.4**	**0.5**	**0.6**	**0.5**	**0.6**
Leu 75	-2.30	**-2.2**	**-1.8**	**-2.0**	**-2.2**	**-1.8**	**-2.1**
Cys 76	1.80	**1.2**	**0.9**	**1.0**	**1.2**	**0.8**	**1.1**
Asn 77	0.62	**0.1**	**0.4**	**0.3**	**0.2**	**0.4**	**0.2**
Pro 79	0.26	**-0.4**	**-0.3**	**-0.3**	**-0.3**	**-0.4**	**-0.3**
Cys 80	-0.23	**-0.6**	**-0.2**	**-0.3**	**-0.5**	**-0.2**	**-0.4**
Ser 81	-1.71	**-1.3**	**-1.3**	**-1.3**	**-1.4**	**-1.2**	**-1.4**
Ala 82	-0.80	**-0.6**	**-0.8**	**-0.7**	**-0.6**	**-0.8**	**-0.7**
Leu 83	1.12	**0.3**	**0.6**	**0.5**	**0.4**	**0.6**	**0.6**
Leu 84	-0.72	**-1.1**	**-0.7**	**-0.9**	**-1.1**	**-0.8**	**-1.0**
Ser 85	-2.60	**-2.6**	**-2.4**	**-2.5**	**-2.6**	**-2.4**	**-2.6**
Ile 88	2.38	**1.0**	**1.1**	**1.1**	**1.1**	**1.1**	**1.2**

Thr 89	-1.36	**-2.3**	**-1.8**	**-2.0**	**-2.3**	**-1.8**	**-2.2**
Ala 90	1.77	**1.4**	**1.2**	**1.3**	**1.4**	**1.2**	**1.3**
Ser 91	1.06	**0.8**	**0.3**	**0.5**	**0.7**	**0.4**	**0.6**
Val 92	1.08	***-0.0***	**0.3**	**0.2**	**0.1**	**0.3**	**0.3**
Asn 93	-1.11	**-1.2**	**-0.8**	**-1.0**	**-1.2**	**-0.9**	**-1.1**
Cys 94	0.75	**1.1**	**0.8**	**0.9**	**1.0**	**0.7**	**0.9**
Ala 95	1.20	**1.4**	**1.1**	**1.2**	**1.4**	**1.2**	**1.3**
Lys 96	-1.79	**-2.0**	**-1.4**	**-1.6**	**-1.9**	**-1.5**	**-1.8**
Lys 97	0.79	**1.2**	**1.1**	**1.2**	**1.2**	**1.1**	**1.2**
Ile 98	-1.68	***0.4***	-0.0	***0.2***	***0.4***	***0.0***	***0.2***
Val 99	1.81	**1.5**	**1.4**	**1.5**	**1.5**	**1.5**	**1.6**
Ser 100	-2.11	**-2.0**	**-1.6**	**-1.8**	**-2.0**	**-1.6**	**-1.9**
Gly 102	0.52	**0.5**	**0.6**	**0.6**	**0.6**	**0.5**	**0.7**
Gly 104	1.94	**1.5**	**1.4**	**1.5**	**1.6**	**1.5**	**1.6**
Met 105	0.41	**0.3**	**0.3**	**0.3**	**0.3**	**0.3**	**0.3**
Asn 106	-0.99	**-0.9**	**-1.2**	**-1.1**	**-1.0**	**-1.2**	**-1.1**
Ala 107	1.44	**1.3**	**1.3**	**1.3**	**1.3**	**1.3**	**1.4**
Ala 110	-1.26	**-0.5**	**-0.9**	**-0.8**	**-0.6**	**-0.8**	**-0.8**
Trp 111	1.12	**1.4**	**1.1**	**1.3**	**1.3**	**1.1**	**1.3**
Asn 113	-2.61	**-2.4**	**-1.8**	**-2.1**	**-2.3**	**-1.9**	**-2.2**
Arg 114	0.28	***-1.1***	***-1.1***	***-1.1***	***-1.1***	***-1.1***	***-1.2***
Cys 115	1.25	**1.5**	**1.1**	**1.3**	**1.4**	**1.2**	**1.3**
Lys 116	-0.35	**-0.0**	**-0.2**	**-0.1**	**-0.0**	**-0.2**	**-0.1**
Thr 118	-0.81	**-0.9**	**-0.8**	**-0.9**	**-1.0**	**-0.8**	**-1.0**
Val 120	-0.92	**-1.0**	**-0.8**	**-0.9**	**-1.1**	**-0.8**	**-1.0**
Gln 121	1.11	**0.6**	**0.8**	**0.8**	**0.7**	**0.8**	**0.8**
Ala 122	0.73	**1.0**	**0.5**	**0.7**	**0.9**	**0.5**	**0.8**
Trp 123	0.43	**0.5**	**0.6**	**0.6**	**0.5**	**0.5**	**0.5**
Ile 124	-3.36	**-2.7**	**-2.3**	**-2.4**	**-2.6**	**-2.3**	**-2.5**
Arg 125	0.76	**0.8**	**0.9**	**0.9**	**0.8**	**0.8**	**0.8**
Gly 126	0.95	**1.3**	**1.2**	**1.2**	**1.3**	**1.1**	**1.3**
Cys 127	0.43	**0.4**	**0.2**	**0.3**	**0.3**	**0.3**	**0.3**
Arg 128	-0.62	**-1.0**	**-0.7**	**-0.8**	**-0.9**	**-0.8**	**-0.8**
RMSD		**0.5**	**0.5**	**0.5**	**0.5**	**0.5**	**0.5**
rRMSD		-	-	-	-	-	-
urRMSD		**0.5**	**0.5**	**0.5**	**0.5**	**0.5**	**0.5**
N_{dev}		**0**	**0**	**0**	**0**	**0**	**0**
$N_{dev,s}$		**6**	**2**	**3**	**3**	**3**	**3**

Table S10. ^{13}C'-^{15}N RDC values (45) in Hz for HEWL, as obtained from NMR measurements at 308 K and pH = 3.8, Table 4.5 of Ref.[16], and as calculated using three different sets of RDC restraints (RDC_{CAH59}, RDC_{NH101}, $RDC_{NCAH160}$) for the X-ray structure ***1IEE*** by applying the alignment-tensor method (*AT*: $\tau_D^{RDC} = 0$, $\tau_{AT}^{RDC} = 0$)[13] or the *HRS* ($K^{RDC,msy} = 0$) method[14]. $K^{RDC,mfv} = 100$ kJmol^{-1}Hz^{-2}, $\tau_\theta^{RDC,mfv} = 10$ ns, in $t^{mfv} = 30$ ns SD simulations of the magnetic-field vector. The RDC restraint set RDC_{CAH59} contains the RDC values D_k^0 given in the fourth column of Table 1. The RDC restraint set RDC_{NH101} contains the RDC values D_k^0 given in the second column of Table 2. The RDC restraint set $RDC_{NCAH160}$ is obtained by combining the sets of RDC restraints RDC_{CAH59} and RDC_{NH101}. The values for the RDCs that are *not* part of the (sub)set of RDC restraints applied, are in bold. *RMSD*: Root-mean-square difference (RMSD) between calculated D_k and D_k^0 RDC values calculated over all, *mfv*-restrained and unrestrained, RDCs. *rRMSD*: RMSD-values calculated over the particular (sub)set of *mfv*-restrained RDCs. *urRMSD*: RMSD-values calculated over the unrestrained RDCs. Deviations of RDC-values $D_{k_1k_2}$ (*AT*) or averaged $< D_{k_1k_2} >_{t^{mfv}}$ (*HRS*) from the D_k^0 values larger than 3 Hz are in red. N_{dev}: Number of such deviations. $N_{dev,s}$: Number of RDCs for which the calculated D_k and the D_k^0 values have a different sign. These RDC values are in italics.

Residue	Experimental value (Hz)	*AT*			*HRS*		
		CAH59	*NH101*	*NCAH160*	*CAH59*	*NH101*	*NCAH160*
Gly 4	-0.55	**-0.4**	**-0.2**	**-0.3**	**-0.4**	**-0.2**	**-0.3**
Glu 7	0.20	**0.1**	***-0.0***	**0.0**	**0.1**	**0.0**	**0.1**
Leu 8	0.67	**0.6**	**0.7**	**0.7**	**0.7**	**0.7**	**0.7**
Ala 9	-0.38	***0.0***	**-0.1**	**-0.1**	***0.0***	**-0.1**	***0.0***
Ala 10	0.42	**0.4**	**0.6**	**0.6**	**0.5**	**0.6**	**0.6**
Ala 11	-0.95	**-0.8**	**-0.8**	**-0.8**	**-0.8**	**-0.7**	**-0.8**
Met 12	2.41	**1.7**	**1.5**	**1.6**	**1.7**	**1.5**	**1.7**
Lys 13	-0.78	**-0.8**	**-0.7**	**-0.8**	**-0.8**	**-0.7**	**-0.8**
Arg 14	1.07	**0.7**	**0.6**	**0.7**	**0.7**	**0.6**	**0.7**
Gly 16	-0.95	**-0.9**	**-0.9**	**-0.9**	**-0.9**	**-0.9**	**-1.0**
Trp 28	1.37	**1.7**	**1.4**	**1.5**	**1.7**	**1.4**	**1.6**
Ala 31	-0.23	**-0.1**	**-0.2**	**-0.2**	**-0.1**	**-0.2**	**-0.1**
Ala 32	0.38	**0.9**	**0.9**	**0.9**	**0.9**	**0.9**	**0.9**
Lys 33	-0.49	**-0.3**	**-0.4**	**-0.3**	**-0.3**	**-0.3**	**-0.3**

Asn 37	-1.12	**-0.9**	**-0.9**	**-1.0**	**-1.0**	**-1.0**	**-1.0**
Phe 38	1.72	**1.1**	**1.1**	**1.2**	**1.2**	**1.1**	**1.2**
Asn 39	-0.78	**-0.8**	**-0.8**	**-0.8**	**-0.8**	**-0.7**	**-0.8**
Thr 40	-1.41	**-1.0**	**-0.8**	**-0.9**	**-1.0**	**-0.9**	**-1.0**
Gln 41	-0.26	**-0.5**	**-0.3**	**-0.4**	**-0.5**	**-0.3**	**-0.4**
Thr 47	-1.13	**-0.9**	**-0.8**	**-0.9**	**-0.9**	**-0.9**	**-0.9**
Arg 61	1.21	**1.3**	**1.4**	**1.4**	**1.4**	**1.4**	**1.4**
Arg 68	-0.64	**-0.9**	**-0.8**	**-0.8**	**-0.9**	**-0.8**	**-0.9**
Leu 75	-0.57	**-0.2**	**-0.4**	**-0.3**	**-0.3**	**-0.4**	**-0.4**
Cys 76	1.35	**1.6**	**1.5**	**1.6**	**1.6**	**1.5**	**1.6**
Asn 77	-0.81	**-1.0**	**-0.9**	**-0.9**	**-1.0**	**-0.9**	**-1.0**
Ser 81	-1.23	**-1.0**	**-1.0**	**-1.0**	**-1.0**	**-1.0**	**-1.0**
Leu 83	-0.89	**-0.8**	**-0.6**	**-0.7**	**-0.8**	**-0.6**	**-0.7**
Leu 84	-0.63	**-1.0**	**-0.9**	**-0.9**	**-1.0**	**-0.9**	**-1.0**
Ala 90	-0.78	**-0.3**	**-0.4**	**-0.4**	**-0.3**	**-0.4**	**-0.4**
Asn 93	-0.28	**-0.5**	**-0.3**	**-0.4**	**-0.4**	**-0.4**	**-0.4**
Ala 95	-0.40	**-0.7**	**-0.6**	**-0.6**	**-0.6**	**-0.6**	**-0.6**
Lys 96	1.14	**0.9**	**1.0**	**1.0**	**1.0**	**1.0**	**1.0**
Lys 97	-1.40	**-0.9**	**-0.9**	**-0.9**	**-0.9**	**-0.9**	**-0.9**
Gly 104	0.60	**1.1**	**0.8**	**0.9**	**1.0**	**0.8**	**0.9**
Met 105	-0.05	**-0.1**	**-0.2**	**-0.2**	**-0.1**	**-0.2**	**-0.2**
Ala 110	-1.01	**-0.9**	**-0.9**	**-0.9**	**-0.9**	**-0.9**	**-0.9**
Trp 111	1.13	**1.3**	**1.0**	**1.2**	**1.3**	**1.0**	**1.2**
Asn 113	-0.28	**-0.1**	**-0.2**	**-0.2**	**-0.1**	**-0.2**	**-0.1**
Arg 114	-1.14	**-0.9**	**-0.9**	**-0.9**	**-0.9**	**-0.9**	**-1.0**
Cys 115	0.97	**1.1**	**0.8**	**0.9**	**1.0**	**0.8**	**0.9**
Gln 121	-0.78	**-0.9**	**-0.8**	**-0.9**	**-0.9**	**-0.8**	**-0.9**
Ala 122	1.68	**1.2**	**1.0**	**1.1**	**1.2**	**1.1**	**1.1**
Arg 125	1.26	**1.3**	**1.2**	**1.3**	**1.3**	**1.2**	**1.3**
Gly 126	0.52	**1.0**	**0.7**	**0.8**	**0.9**	**0.7**	**0.8**
Cys 127	-0.67	**-0.7**	**-0.7**	**-0.7**	**-0.7**	**-0.6**	**-0.7**
RMSD		**0.3**	**0.3**	**0.3**	**0.3**	**0.3**	**0.3**
rRMSD		-	-	-	-	-	-
urRMSD		**0.3**	**0.3**	**0.3**	**0.3**	**0.3**	**0.3**
N_{dev}		**0**	**0**	**0**	**0**	**0**	**0**
$N_{dev,s}$		**1**	**1**	**0**	**1**	**0**	**1**

Table S11. $^{13}C^{\alpha}$-$^{1}H^{\alpha}$ RDC values (38, 39) in Hz for HEWL, as obtained from NMR measurements at 308 K and pH = 3.8 using two different pulse sequences as given in Tables 4.2 and 4.3 of Ref.[16], and as calculated using three different sets of RDC restraints (RDC_{CAH59}, RDC_{NH101}, $RDC_{NCAH160}$) for the X-ray structure ***1AKI*** by applying the alignment-tensor method (*AT*: $\tau_D^{RDC} = 0$, $\tau_{AT}^{RDC} = 0$)[13] or the *HRS* ($K^{RDC,msy} = 0$) method[14], $K^{RDC,mfv} = 100$ kJmol^{-1}Hz^{-2}, $\tau_\theta^{RDC,mfv} = 10$ ns, in $t^{mfv} = 30$ ns SD simulations of the magnetic-field vector. The (RDC restraint) set RDC_{CAH59} contains the RDC values D_k^0 given in the fourth column. The RDC restraint set RDC_{NH101} contains the RDC values D_k^0 given in the second column of Table 2. The RDC restraint set $RDC_{NCAH160}$ is obtained by combining the sets of RDC restraints RDC_{CAH59} and RDC_{NH101}. In case two experimental values are available, the D_k^0 RDC values used in the calculations are the average of the two experimental values. The values for the RDCs that are *not* part of the (sub)set of RDC restraints applied, are in bold. *RMSD*: Root-mean-square difference (RMSD) between calculated D_k and D_k^0 RDC values calculated over all, *mfv*-restrained and unrestrained, RDCs. *rRMSD*: RMSD-values calculated over the particular (sub)set of *mfv*-restrained RDCs. *urRMSD*: RMSD-values calculated over the unrestrained RDCs. Deviations of RDC values $D_{k_1k_2}$ (*AT*) or averaged $< D_{k_1k_2} >_{t^{mfv}}$ (*HRS*) from the D_k^0 values larger than 3 Hz are in red. N_{dev}: Number of such deviations. $N_{dev,s}$: Number of RDCs for which the calculated D_k and the D_k^0 values have a different sign. These RDC values are in italics.

Residue	Experimental value (Hz)			*AT*			*HRS*		
	Table 4.2[16]	Table 4.3[16]	D_k^0	*CAH59*	*NH101*	*NCAH160*	*CAH59*	*NH101*	*NCAH160*
Val 2	-5.30	-	-5.30	-9.3	**-5.2**	-6.8	-8.9	**-4.8**	-7.6
Phe 3	-	8.85	8.85	7.1	**8.5**	8.1	7.1	**7.5**	6.6
Cys 6	-	15.30	15.30	13.5	**12.8**	13.4	13.7	**11.6**	14.0
Glu 7	-	-6.52	-6.52	-5.2	**-3.4**	-4.5	-6.0	**-3.4**	-5.6
Leu 8	-	3.17	3.17	1.4	***-1.0***	0.1	1.6	***-1.3***	0.1
Met 12	-20.12	-22.20	-21.16	-21.0	**-20.8**	-21.0	-21.1	**-19.8**	-21.1

Lys 13	10.26	10.33	10.30	12.2	**11.9**	12.3	12.3	**10.4**	12.3
His 15	15.60	-	15.60	15.3	**14.2**	14.8	16.1	**14.0**	14.4
Leu 17	-	-10.66	-10.66	-14.4	**-10.8**	-12.3	-13.4	**-9.0**	-12.1
Asp 18	-	19.61	19.61	15.8	**15.2**	15.4	16.5	**15.7**	15.6
Tyr 23	-	13.81	13.81	12.0	**9.5**	10.8	12.2	**8.3**	10.4
Leu 25	-18.58	-	-18.58	-16.5	**-17.2**	-17.0	-16.6	**-16.6**	-17.0
Trp 28	-28.74	-28.51	-28.62	-29.8	**-26.2**	-28.0	-30.7	**-25.3**	-29.3
Val 29	5.17	-	5.17	7.2	**3.5**	5.2	6.5	**1.7**	5.2
Cys 30	9.83	8.84	9.34	3.0	**4.6**	4.0	4.6	**6.2**	5.3
Lys 33	13.60	-	13.60	11.1	**11.2**	11.5	11.2	**9.8**	11.1
Phe 34	-	15.36	15.36	14.7	**14.4**	14.6	16.1	**15.6**	16.3
Glu 35	-	-5.65	-5.65	-2.4	***0.3***	-0.9	-2.8	**-0.0**	-2.6
Asn 39	-6.88	-4.05	-5.46	-6.4	**-2.7**	-4.2	-6.5	**-2.9**	-5.6
Thr 40	15.69	15.81	15.75	13.8	**12.9**	13.6	13.9	**11.6**	14.1
Ala 42	-	18.93	18.93	16.8	**15.1**	15.8	17.2	**15.3**	17.5
Asn 44	21.03	-	21.03	16.9	**15.6**	16.2	17.6	**16.0**	17.9
Thr 47	-	12.40	12.40	14.1	**11.2**	12.5	13.7	**10.0**	13.8
Asp 48	-17.97	-18.29	-18.13	-20.4	**-17.3**	-18.9	-21.5	**-16.9**	-20.4
Thr 51	16.96	-	16.96	15.8	**14.9**	15.4	17.0	**15.6**	15.7
Asp 52	-	-5.43	-5.43	*14.9*	***14.6***	*14.8*	*16.3*	***15.7***	*16.5*
Leu 56	-0.83	-	-0.83	*0.2*	**-1.4**	-0.7	*0.8*	**-1.0**	-0.5
Gln 57	6.72	-	6.72	8.0	**4.1**	5.8	7.0	**2.1**	6.5
Asn 59	17.60	-	17.60	16.6	**15.1**	15.8	17.1	**15.0**	17.5
Trp 62	-19.22	-22.42	-20.82	-24.9	**-21.5**	-23.0	-24.5	**-19.7**	-23.3
Trp 63	17.30	-	17.30	10.5	**11.0**	11.0	12.0	**12.4**	12.5
Cys 64	18.24	-	18.24	16.8	**16.1**	16.4	17.7	**16.8**	17.0
Asn 65	0.76	-	0.76	0.9	**3.4**	2.4	0.7	**2.8**	0.8
Pro 70	5.72	-	5.72	0.9	***-0.7***	0.1	1.6	***-0.3***	0.2
Ile 78	-	17.81	17.81	15.2	**14.0**	14.7	15.7	**13.4**	14.0
Ser 81	14.90	-	14.90	15.4	**14.7**	15.1	16.8	**15.9**	16.0
Leu 84	-	5.15	5.15	3.7	**1.2**	2.4	3.9	**0.6**	2.3
Ser 85	-	13.38	13.38	12.6	**9.2**	10.8	11.9	**7.2**	11.5
Ile 88	-14.50	-15.25	-14.88	-14.2	**-14.5**	-14.8	-15.8	**-14.7**	-15.0
Thr 89	-0.08	-3.00	-1.54	-6.8	**-10.2**	-8.9	-8.2	**-11.5**	-8.6
Ala 90	-	17.24	17.24	14.4	**14.1**	14.3	16.0	**15.5**	15.8
Val 92	14.69	-	14.69	14.3	**12.6**	13.5	14.7	**11.7**	12.9
Asn 93	-	10.98	10.98	*-3.3*	***-6.0***	*-5.1*	*-4.8*	***-6.9***	*-4.5*
Lys 96	-1.72	-	-1.72	-2.6	**-5.4**	-4.1	-2.8	**-6.0**	-4.1
Ile 98	-26.52	-27.59	-27.06	-28.5	**-24.6**	-26.4	-28.6	**-23.0**	-27.2
Val 99	-	18.49	18.49	12.2	**12.5**	12.4	12.5	**12.3**	11.5
Ser 100	-8.22	-	-8.22	-5.2	**-8.8**	-7.4	-6.6	**-10.1**	-7.1
Trp 108	-10.09	-	-10.09	-5.0	**-8.6**	-7.1	-6.4	**-9.9**	-6.8
Val 109	14.56	19.08	16.82	14.8	**13.2**	14.2	14.8	**11.9**	15.0
Trp 111	-22.31	-22.56	-22.44	-23.3	**-18.8**	-20.8	-22.9	**-17.3**	-21.4
Arg 112	-	12.73	12.73	6.9	**4.2**	5.1	5.9	**3.5**	6.2
Cys 115	-	-21.39	-21.39	-20.3	**-17.3**	-18.6	-19.5	**-15.3**	-18.6
Lys 116	8.87	3.30	6.08	6.5	**6.6**	6.3	6.2	**6.7**	6.1
Thr 118	12.69	-	12.69	8.0	**5.4**	6.7	8.3	**4.7**	6.5
Asp 119	17.09	15.66	16.37	16.7	**14.9**	15.7	17.1	**15.0**	17.4
Val 120	13.71	13.50	13.65	14.5	**12.4**	13.1	14.4	**12.3**	14.6

Gln 121	12.41	9.50	10.95	12.6	**10.9**	11.8	13.4	**10.8**	11.6
Ala 122	-	-30.56	-30.56	-31.3	**-28.8**	-30.2	-32.1	**-27.7**	-31.0
Cys 127	14.73	16.44	15.58	14.8	**13.5**	14.3	15.0	**12.5**	15.3
RMSD				4.3	**4.8**	4.4	4.3	**5.3**	4.4
rRMSD				4.3	-	4.4	4.3	-	4.4
urRMSD				-	**4.8**	-	-	**5.3**	-
N_{dev}				17	**24**	16	15	**31**	15
$N_{dev,s}$				2	**5**	2	2	**4**	2

Table S12. ^{15}N-$^{1}H^{N}$ RDC values (101) in Hz for HEWL, as obtained from NMR measurements at 308 K and pH = 3.8, Table 4.1 of Ref.[16], and as calculated using three different sets of RDC restraints (RDC_{CAH59}, RDC_{NH101}, $RDC_{NCAH160}$) for the X-ray structure ***1AKI*** by applying the alignment-tensor method (*AT*: $\tau_D^{RDC} = 0$, $\tau_{AT}^{RDC} = 0$)[13] or the *HRS* ($K^{RDC,msy} = 0$) method[14], $K^{RDC,mfv} = 100$ kJmol^{-1}Hz^{-2}, $\tau_\theta^{RDC,mfv} = 10$ ns, in $t^{mfv} = 30$ ns SD simulations of the magnetic-field vector. The RDC restraint set RDC_{CAH59} contains the RDC values D_k^0 given in the fourth column of Table 1. The RDC restraint set RDC_{NH101} contains the RDC values D_k^0 given in the second column. The RDC restraint set $RDC_{NCAH160}$ is obtained by combining the sets of RDC restraints RDC_{CAH59} and RDC_{NH101}. The values for the RDCs that are *not* part of the (sub)set of RDC restraints applied, are in bold. *RMSD*: Root-mean-square difference (RMSD) between calculated D_k and D_k^0 RDC values calculated over all, *mfv*-restrained and unrestrained, RDCs. *rRMSD*: RMSD-values calculated over the particular (sub)set of *mfv*-restrained RDCs. *urRMSD*: RMSD-values calculated over the unrestrained RDCs. Deviations of RDC values $D_{k_1k_2}$ (*AT*) or averaged $< D_{k_1k_2} >_{t^{mfv}}$ (*HRS*) from the D_k^0 values larger than 3 Hz are in red. N_{dev}: Number of such deviations. $N_{dev,s}$: Number of RDCs for which the calculated D_k and the D_k^0 values have a different sign. These RDC values are in italics.

Residue	Experimental value (Hz), target D_k^0	*AT*			*HRS*		
		CAH59	*NH101*	*NCAH160*	*CAH59*	*NH101*	*NCAH160*
Val 2	-1.78	**-2.4**	-3.4	-3.1	**-2.7**	-3.2	-3.1
Phe 3	7.52	**10.7**	8.2	9.3	**10.7**	7.7	9.9
Gly 4	-6.92	**-6.5**	-6.7	-6.6	**-6.7**	-6.4	-6.1
Arg 5	-6.47	**-7.4**	-7.3	-7.4	**-8.2**	-8.0	-8.1
Cys 6	-3.68	**-4.7**	-5.1	-5.0	**-5.5**	-5.8	-5.8
Glu 7	-8.56	**-9.0**	-8.5	-8.7	**-9.5**	-8.9	-9.4
Leu 8	-7.85	**-7.9**	-7.6	-7.8	**-8.7**	-8.3	-8.4
Ala 9	-4.27	**-6.2**	-6.1	-6.2	**-7.0**	-7.0	-6.9
Ala 11	-8.64	**-8.8**	-8.4	-8.6	**-9.5**	-9.0	-9.4
Lys 13	-3.36	**-3.4**	-4.1	-3.9	**-4.3**	-4.8	-4.6
Arg 14	-7.36	**-8.1**	-7.9	-8.0	**-8.8**	-8.3	-8.9
Gly 16	12.38	**10.5**	8.5	9.3	**10.1**	7.5	9.4

Leu 17	-4.59	**-3.4**	-4.2	-4.0	**-3.8**	-4.0	-4.2
Asn 19	-5.96	**-9.0**	-8.5	-8.7	**-9.5**	-9.0	-9.3
Tyr 20	0.61	***-5.8***	*-6.0*	*-6.1*	***-6.3***	*-6.1*	*-6.7*
Arg 21	-6.77	**-7.1**	-7.0	-7.0	**-7.3**	-7.2	-7.0
Gly 22	-2.70	**-2.6**	-3.5	-3.2	**-2.5**	-3.2	-2.3
Tyr 23	0.23	***-4.2***	*-3.1*	*-3.7*	***-4.6***	*-3.0*	*-3.6*
Ser24	-6.59	**-7.5**	-6.8	-7.2	**-7.5**	-5.9	-6.8
Gly 26	4.19	**2.2**	3.7	3.2	**3.0**	4.2	2.9
Asn 27	-5.76	**-4.9**	-3.8	-4.2	**-4.6**	-3.7	-4.7
Trp 28	5.29	**1.3**	1.8	1.7	**1.9**	1.9	1.6
Val 29	3.56	**1.8**	3.1	2.7	**2.6**	3.5	2.4
Ala 31	-3.83	**-4.8**	-3.9	-4.1	**-4.5**	-3.9	-4.6
Ala 32	4.34	***-0.1***	0.5	0.4	**0.5**	0.7	0.2
Lys 33	0.35	***-3.0***	*-1.3*	*-1.9*	***-2.4***	*-0.7*	*-2.5*
Phe 34	-4.22	**-7.9**	-6.7	-7.2	**-7.9**	-6.6	-8.0
Glu 35	3.73	**4.3**	4.2	4.5	**5.0**	4.2	4.7
Ser 36	7.51	**7.7**	8.4	8.3	**8.7**	8.6	8.3
Asn 37	-7.43	**-7.0**	-6.5	-6.9	**-7.0**	-5.6	-6.9
Phe 38	-0.76	***0.5***	-1.9	-1.4	**-0.8**	-1.9	-1.4
Asn 39	-2.69	**-1.0**	-2.3	-1.8	**-1.0**	-1.9	-1.1
Thr 40	6.48	**9.2**	6.8	7.8	**9.1**	6.3	8.3
Gln 41	6.78	**5.6**	5.1	5.3	**5.0**	4.2	5.1
Ala 42	10.72	**9.2**	9.5	9.6	**10.1**	9.6	9.7
Thr 43	-6.94	**-7.7**	-7.5	-7.6	**-8.4**	-8.1	-8.5
Asn 44	-8.88	**-7.7**	-7.5	-7.6	**-8.5**	-8.2	-8.4
Arg 45	-7.32	**-6.2**	-6.3	-6.3	**-7.0**	-7.0	-7.1
Asn 46	-4.82	**-4.7**	-4.0	-4.4	**-5.3**	-4.3	-4.5
Thr 47	4.71	**4.2**	5.8	5.1	**4.7**	6.2	5.1
Asp 48	14.01	**12.9**	13.1	13.2	**13.7**	13.1	13.4
Gly 49	1.82	***-2.2***	*-2.3*	*-2.3*	***-3.1***	*-3.2*	*-2.8*
Thr 51	-7.92	**-8.0**	-7.7	-7.8	**-8.7**	-8.3	-8.3
Gly 54	-6.58	**-6.0**	-4.8	-5.2	**-5.8**	-4.6	-5.9
Ile 55	-7.54	**-8.0**	-7.6	-7.8	**-8.2**	-7.3	-7.4
Leu 56	-0.14	**-3.8**	-2.6	-3.2	**-4.0**	-2.4	-3.1
Gln 57	-3.19	**-2.4**	-3.4	-3.1	**-2.6**	-3.0	-2.9
Ile 58	-3.83	**-2.7**	-3.7	-3.4	**-2.7**	-3.1	-2.8
Asn 59	-3.85	**-5.7**	-3.8	-4.6	**-5.3**	-2.9	-5.2
Ser 60	-7.82	**-8.8**	-8.4	-8.5	**-9.4**	-9.0	-9.3
Trp 63	-8.15	**-9.0**	-8.5	-8.7	**-9.5**	-8.9	-9.4
Asn 65	1.18	**0.6**	*-0.5*	0.0	**0.8**	*-0.5*	0.7
Asp 66	-0.81	**-2.2**	-3.3	-2.9	**-2.2**	-2.8	-2.2
Gly 67	-7.13	**-7.4**	-5.8	-6.5	**-7.1**	-5.2	-7.2
Arg 68	13.60	**12.7**	13.0	13.0	**13.5**	12.9	13.2
Thr 69	-3.43	**-8.1**	-7.9	-8.0	**-8.4**	-8.0	-7.9
Gly 71	2.44	**5.0**	6.0	5.6	**5.1**	6.1	5.6
Arg 73	-1.26	***5.2***	*4.6*	*4.8*	***4.6***	*3.6*	*4.6*
Asn 74	-7.40	**-9.0**	-8.5	-8.7	**-9.5**	-9.0	-9.2
Cys 76	-4.97	**-5.1**	-4.3	-4.5	**-4.8**	-4.3	-4.9
Ile 78	-1.37	**-4.3**	-4.9	-4.7	**-4.3**	-4.6	-4.0
Cys 80	16.06	**16.1**	14.8	15.5	**16.7**	14.3	16.0

Ser 81	14.37	**14.1**	13.4	13.8	**14.2**	12.7	13.9
Ala 82	7.74	**9.9**	7.7	8.6	**9.5**	6.8	8.7
Leu 83	15.17	**15.5**	14.2	14.9	**16.1**	13.8	15.5
Leu 84	5.43	**4.1**	4.8	4.5	**3.9**	4.5	4.4
Ser 86	-4.45	**-5.6**	-4.1	-4.9	**-5.5**	-3.2	-4.7
Asp 87	12.03	**6.6**	7.9	7.4	**7.1**	8.2	7.4
Thr 89	-2.93	**-2.9**	-3.8	-3.5	**-3.0**	-3.3	-3.2
Ser 91	-0.10	**-1.1**	-2.4	-2.0	**-1.5**	-2.3	-1.9
Val 92	-4.32	**-2.8**	-3.7	-3.5	**-2.9**	-3.3	-3.2
Asn 93	-6.06	**-6.9**	-6.5	-6.8	**-6.9**	-5.7	-6.9
Cys 94	-5.79	**-5.4**	-5.7	-5.7	**-5.7**	-5.5	-6.1
Ala 95	-2.42	**-1.7**	-2.9	-2.5	**-1.9**	-2.6	-2.3
Lys 96	-6.08	**-5.9**	-5.9	-6.1	**-5.9**	-5.1	-5.7
Lys 97	-7.62	**-7.6**	-6.8	-7.3	**-7.5**	-6.0	-7.6
Ile 98	-4.26	**-2.6**	-3.6	-3.3	**-2.9**	-3.3	-3.2
Ser 100	-4.10	**-7.1**	-5.6	-6.4	**-6.8**	-4.5	-6.4
Gly 102	8.96	**9.4**	7.1	8.1	**9.1**	6.4	8.4
Asn 103	-7.26	**-4.9**	-3.1	-4.0	**-4.6**	-2.1	-4.0
Gly 104	-4.67	**-3.8**	-3.8	-3.9	**-4.7**	-4.7	-4.4
Met 105	9.39	**3.6**	5.4	4.7	**4.3**	6.0	4.5
Asn 106	-7.82	**-8.8**	-8.0	-8.4	**-9.1**	-8.1	-9.3
Ala 107	-1.63	**-2.8**	-2.3	-2.3	**-2.4**	-2.3	-2.5
Trp 108	3.79	**0.9**	2.7	2.0	**1.7**	3.3	1.7
Val 109	7.54	**2.1**	3.9	3.1	**2.6**	4.6	3.1
Trp 111	9.71	**5.2**	5.9	5.6	**5.1**	5.6	5.6
Arg 112	8.40	**5.3**	6.3	5.9	**5.4**	6.3	5.9
Asn 113	1.87	***-2.7***	*-0.9*	*-1.8*	***-2.5***	*-0.1*	*-1.7*
Cys 115	14.32	**14.6**	13.1	13.8	**14.6**	12.3	14.1
Lys 116	12.40	**10.7**	11.3	11.3	**11.6**	11.5	11.4
Gly 117	-7.17	**-8.1**	-7.9	-7.9	**-8.4**	-8.1	-8.0
Val 120	-4.37	**-4.8**	-5.3	-5.2	**-5.2**	-5.2	-5.6
Gln 121	-6.83	**-7.3**	-6.5	-7.0	**-7.3**	-5.5	-6.9
Ala 122	-0.89	**-3.0**	-3.9	-3.7	**-3.1**	-3.4	-3.0
Ile 124	-5.73	**-7.3**	-6.1	-6.7	**-7.1**	-5.0	-6.7
Arg 125	1.26	**2.1**	0.6	1.3	**2.3**	0.7	2.2
Gly 126	-2.46	**-3.6**	-2.3	-2.8	**-3.2**	-2.1	-3.3
Cys 127	-3.28	**-5.7**	-5.8	-6.0	**-5.8**	-5.2	-5.9
Arg 128	-4.94	**-6.9**	-5.2	-6.0	**-6.6**	-4.5	-6.6
Leu 129	1.83	**1.4**	3.2	2.4	**1.8**	3.7	2.4
RMSD		**2.3**	2.0	2.0	**2.2**	2.1	2.1
rRMSD		-	2.0	2.0	-	2.1	2.1
urRMSD		**2.3**	-	-	**2.2**	-	-
N_{dev}		**18**	16	17	**16**	15	16
$N_{dev,s}$		**7**	7	5	**6**	7	6

Table S13. $^{13}C^{\alpha}$-$^{13}C'$ RDC values (97) in Hz for HEWL, as obtained from NMR measurements at 308 K and pH = 3.8, Table 4.4 of Ref.[16], and as calculated using three different sets of RDC restraints (RDC_{CAH59}, RDC_{NH101}, $RDC_{NCAH160}$) for the X-ray structure ***1AKI*** by applying the alignment-tensor method (*AT*: $\tau_D^{RDC} = 0$, $\tau_{AT}^{RDC} = 0$)[13] or the *HRS* ($K^{RDC,msy} = 0$) method[14]. $K^{RDC,mfv} = 100$ kJmol^{-1}Hz^{-2}, $\tau_\theta^{RDC,mfv} = 10$ ns, in $t^{mfv} = 30$ ns SD simulations of the magnetic-field vector. The RDC restraint set RDC_{CAH59} contains the RDC values D_k^0 given in the fourth column of Table 1. The RDC restraint set RDC_{NH101} contains the RDC values D_k^0 given in the second column of Table 2. The RDC restraint set $RDC_{NCAH160}$ is obtained by combining the sets of RDC restraints RDC_{CAH59} and RDC_{NH101}. The values for the RDCs that are *not* part of the (sub)set of RDC restraints applied, are in bold. *RMSD*: Root-mean-square difference (RMSD) between calculated D_k and D_k^0 RDC values calculated over all, *mfv*-restrained and unrestrained, RDCs. *rRMSD*: RMSD-values calculated over the particular (sub)set of *mfv*-restrained RDCs. *urRMSD*: RMSD-values calculated over the unrestrained RDCs. Deviations of RDC-values $D_{k_1k_2}$ (*AT*) or averaged $< D_{k_1k_2} >_{t^{mfv}}$ (*HRS*) from the D_k^0 values larger than 3 Hz are in red. N_{dev}: Number of such deviations. $N_{dev,s}$: Number of RDCs for which the calculated D_k and D_k^0 values have a different sign. These RDC values are in italics.

Residue	Experimental value (Hz)	*AT*			*HRS*		
		CAH59	*NH101*	*NCAH160*	*CAH59*	*NH101*	*NCAH160*
Lys 1	0.97	**1.3**	**1.1**	**1.2**	**1.3**	**0.9**	**1.1**
Val 2	-2.11	**-1.4**	**-1.4**	**-1.5**	**-1.6**	**-1.5**	**-1.5**
Phe 3	-0.50	**-0.4**	**-0.5**	**-0.4**	**-0.3**	**-0.4**	**-0.4**
Gly 4	1.95	**1.4**	**1.2**	**1.3**	**1.4**	**1.1**	**1.4**
Glu 7	-1.56	**-1.2**	**-0.8**	**-1.0**	**-1.1**	**-0.7**	**-1.0**
Leu 8	1.19	**1.2**	**0.9**	**1.0**	**1.1**	**0.8**	**1.1**
Ala 9	1.19	**1.3**	**1.3**	**1.3**	**1.3**	**1.3**	**1.2**
Ala 10	-1.30	**-1.3**	**-1.3**	**-1.3**	**-1.2**	**-1.2**	**-1.3**
Ala 11	0.13	**0.4**	**0.5**	**0.5**	**0.4**	**0.6**	**0.5**
Met 12	0.83	**0.7**	**0.5**	**0.5**	**0.6**	**0.4**	**0.6**
Lys 13	1.51	**1.3**	**1.2**	**1.2**	**1.4**	**1.2**	**1.2**
Arg 14	-1.94	**-2.2**	**-1.9**	**-2.0**	**-2.1**	**-1.7**	**-2.0**
His 15	1.01	**1.2**	**1.1**	**1.2**	**1.2**	**1.0**	**1.2**
Gly 16	0.55	**0.8**	**0.9**	**0.8**	**0.8**	**0.8**	**0.7**

Asp 18	1.71	**1.2**	**1.1**	**1.2**	**1.2**	**1.1**	**1.3**
Asn 19	-1.81	**-1.9**	**-1.8**	**-1.9**	**-1.9**	**-1.6**	**-1.9**
Tyr 23	-0.67	**-1.1**	**-0.7**	**-0.9**	**-1.1**	**-0.7**	**-1.0**
Ser24	1.34	**1.3**	**1.0**	**1.1**	**1.3**	**0.9**	**1.3**
Leu 25	0.76	**1.5**	**1.4**	**1.4**	**1.5**	**1.5**	**1.5**
Asn 27	-0.39	***0.3***	**-0.1**	***0.1***	***0.2***	**-0.2**	***0.1***
Trp 28	1.41	**1.3**	**1.3**	**1.3**	**1.4**	**1.3**	**1.4**
Cys 30	-2.86	**-2.6**	**-2.5**	**-2.6**	**-2.7**	**-2.5**	**-2.6**
Ala 31	1.21	**1.2**	**1.0**	**1.1**	**1.2**	**0.8**	**1.2**
Ala 32	1.67	**1.4**	**1.3**	**1.4**	**1.5**	**1.5**	**1.5**
Lys 33	-0.74	**-0.9**	**-0.7**	**-0.8**	**-1.0**	**-0.7**	**-0.9**
Phe 34	-2.53	**-2.2**	**-2.2**	**-2.2**	**-2.3**	**-2.2**	**-2.3**
Ser 36	-1.04	**-1.1**	**-0.7**	**-0.9**	**-0.9**	**-0.6**	**-0.8**
Asn 37	0.81	**0.5**	**0.6**	**0.6**	**0.5**	**0.6**	**0.4**
Phe 38	0.83	**0.7**	**0.3**	**0.5**	**0.6**	**0.1**	**0.5**
Asn 39	-0.43	**-0.4**	**-0.3**	**-0.4**	**-0.3**	**-0.2**	**-0.3**
Thr 40	-2.42	**-2.1**	**-2.2**	**-2.2**	**-2.3**	**-2.2**	**-2.2**
Gln 41	0.14	***-0.1***	**0.2**	**0.1**	***-0.1***	**0.1**	***-0.1***
Ala 42	-2.63	**-2.1**	**-1.8**	**-1.9**	**-2.0**	**-1.6**	**-1.9**
Asn 44	-1.67	**-2.4**	**-2.0**	**-2.2**	**-2.3**	**-1.9**	**-2.2**
Asn 46	-2.51	**-2.5**	**-2.3**	**-2.4**	**-2.6**	**-2.2**	**-2.5**
Thr 47	-0.95	**-0.5**	**-0.4**	**-0.4**	**-0.3**	**-0.2**	**-0.3**
Asp 48	1.48	**1.2**	**1.0**	**1.2**	**1.3**	**0.9**	**1.1**
Gly 49	-0.60	**-1.0**	**-0.8**	**-0.9**	**-1.1**	**-0.8**	**-1.1**
Ser 50	-1.22	**-1.1**	**-0.8**	**-0.9**	**-1.0**	**-0.6**	**-0.9**
Thr 51	-1.57	**-0.9**	**-1.0**	**-1.0**	**-1.0**	**-1.1**	**-1.0**
Tyr 53	-1.29	**-2.4**	**-2.3**	**-2.4**	**-2.6**	**-2.3**	**-2.5**
Leu 56	-2.63	**-2.3**	**-2.0**	**-2.1**	**-2.4**	**-1.9**	**-2.3**
Gln 57	-0.66	**-0.2**	***0.0***	**-0.1**	**-0.1**	***0.2***	***0.0***
Asn 59	-1.71	**-0.5**	**-0.6**	**-0.5**	**-0.4**	**-0.5**	**-0.5**
Ser 60	-2.19	**-1.3**	**-0.9**	**-1.1**	**-1.3**	**-0.8**	**-1.1**
Arg 61	1.21	**1.5**	**1.3**	**1.4**	**1.5**	**1.4**	**1.5**
Cys 64	-2.38	**-1.9**	**-1.9**	**-1.9**	**-2.0**	**-1.9**	**-2.0**
Asn 65	0.92	**1.4**	**1.3**	**1.3**	**1.4**	**1.2**	**1.5**
Asp 66	-2.07	**-2.1**	**-2.1**	**-2.1**	**-2.2**	**-2.2**	**-2.2**
Gly 67	0.08	**0.3**	**0.3**	**0.3**	**0.3**	**0.4**	**0.3**
Arg 68	1.42	**1.3**	**1.1**	**1.2**	**1.3**	**1.0**	**1.2**
Gly 71	-2.77	**-1.8**	**-1.6**	**-1.8**	**-2.0**	**-1.6**	**-1.9**
Asn 74	0.44	**0.6**	**0.4**	**0.5**	**0.5**	**0.3**	**0.6**
Leu 75	-2.30	**-1.7**	**-1.4**	**-1.5**	**-1.8**	**-1.3**	**-1.7**
Cys 76	1.80	**1.2**	**1.0**	**1.1**	**1.2**	**0.9**	**1.0**
Asn 77	0.62	**0.5**	**0.7**	**0.6**	**0.6**	**0.6**	**0.6**
Pro 79	0.26	***-0.4***	***-0.4***	***-0.4***	**-0.3**	**-0.3**	**-0.4**
Cys 80	-0.23	**-0.4**	**-0.1**	**-0.2**	**-0.4**	**-0.1**	**-0.3**
Ser 81	-1.71	**-1.6**	**-1.5**	**-1.5**	**-1.7**	**-1.4**	**-1.6**
Ala 82	-0.80	**-0.6**	**-0.8**	**-0.7**	**-0.6**	**-0.8**	**-0.7**
Leu 83	1.12	**0.5**	**0.6**	**0.6**	**0.6**	**0.8**	**0.7**
Leu 84	-0.72	**-0.8**	**-0.4**	**-0.6**	**-0.8**	**-0.5**	**-0.8**
Ser 85	-2.60	**-1.9**	**-1.9**	**-1.9**	**-1.9**	**-1.8**	**-1.9**
Ile 88	2.38	**0.9**	**1.0**	**1.0**	**1.1**	**1.1**	**1.1**

Thr 89	-1.36	**-2.1**	**-1.6**	**-1.8**	**-2.1**	**-1.6**	**-1.9**
Ala 90	1.77	**1.2**	**1.2**	**1.2**	**1.3**	**1.1**	**1.1**
Ser 91	1.06	**0.7**	**0.3**	**0.5**	**0.6**	**0.2**	**0.6**
Val 92	1.08	**0.0**	**0.2**	**0.2**	**0.2**	**0.4**	**0.2**
Asn 93	-1.11	**-1.3**	**-0.9**	**-1.1**	**-1.3**	**-0.9**	**-1.2**
Cys 94	0.75	**0.9**	**0.7**	**0.8**	**0.9**	**0.5**	**0.8**
Ala 95	1.20	**1.3**	**1.1**	**1.2**	**1.3**	**1.0**	**1.3**
Lys 96	-1.79	**-1.7**	**-1.3**	**-1.4**	**-1.6**	**-1.2**	**-1.5**
Lys 97	0.79	**1.0**	**1.0**	**1.0**	**1.0**	**1.0**	**0.9**
Ile 98	-1.68	***0.5***	***0.1***	***0.3***	***0.4***	**-0.1**	***0.3***
Val 99	1.81	**1.4**	**1.3**	**1.3**	**1.4**	**1.3**	**1.5**
Ser 100	-2.11	**-2.2**	**-1.7**	**-1.9**	**-2.2**	**-1.6**	**-2.0**
Gly 102	0.52	**1.0**	**0.9**	**1.0**	**1.1**	**1.0**	**1.0**
Gly 104	1.94	**1.5**	**1.3**	**1.4**	**1.5**	**1.3**	**1.6**
Met 105	0.41	**0.3**	**0.4**	**0.3**	**0.2**	**0.4**	**0.2**
Asn 106	-0.99	**-0.6**	**-0.9**	**-0.8**	**-0.7**	**-1.0**	**-0.7**
Ala 107	1.44	**1.1**	**1.2**	**1.2**	**1.2**	**1.2**	**1.3**
Ala 110	-1.26	**-0.9**	**-1.1**	**-1.1**	**-1.1**	**-1.2**	**-1.0**
Trp 111	1.12	**1.3**	**1.1**	**1.2**	**1.3**	**1.0**	**1.2**
Asn 113	-2.61	**-2.7**	**-2.4**	**-2.5**	**-2.7**	**-2.3**	**-2.6**
Arg 114	0.28	***-0.9***	***-0.8***	***-0.9***	***-1.0***	***-0.9***	***-0.9***
Cys 115	1.25	**1.4**	**1.1**	**1.2**	**1.3**	**0.9**	**1.3**
Lys 116	-0.35	***0.2***	***0.0***	**-0.1**	***0.3***	**-0.0**	***0.1***
Thr 118	-0.81	**-0.5**	**-0.3**	**-0.4**	**-0.6**	**-0.3**	**-0.5**
Val 120	-0.92	**-1.0**	**-0.6**	**-0.8**	**-1.0**	**-0.6**	**-1.0**
Gln 121	1.11	**0.7**	**0.8**	**0.8**	**0.8**	**0.9**	**0.9**
Ala 122	0.73	**0.9**	**0.6**	**0.8**	**0.9**	**0.5**	**0.8**
Trp 123	0.43	**0.3**	**0.5**	**0.4**	**0.3**	**0.5**	**0.3**
Ile 124	-3.36	**-2.9**	**-2.6**	**-2.7**	**-2.9**	**-2.5**	**-2.8**
Arg 125	0.76	**1.0**	**1.0**	**1.1**	**1.1**	**1.0**	**1.1**
Gly 126	0.95	**1.1**	**0.9**	**1.0**	**1.1**	**0.8**	**1.0**
Cys 127	0.43	**0.4**	**0.4**	**0.3**	**0.3**	**0.4**	**0.3**
Arg 128	-0.62	**-0.8**	**-0.7**	**-0.7**	**-0.7**	**-0.5**	**-0.7**
RMSD		**0.5**	**0.5**	**0.5**	**0.5**	**0.5**	**0.5**
rRMSD		-	-	-	-	-	-
urRMSD		**0.5**	**0.5**	**0.5**	**0.5**	**0.5**	**0.5**
N_{dev}		**0**	**0**	**0**	**0**	**0**	**0**
$N_{dev,s}$		**6**	**5**	**4**	**5**	**2**	**6**

Table S14. ^{13}C'-^{15}N RDC values (45) in Hz for HEWL, as obtained from NMR measurements at 308 K and pH = 3.8, Table 4.5 of Ref.[16], and as calculated using three different sets of RDC restraints (*RDC_{CAH59}*, ...) for the X-ray structure ***1AKI*** by applying the alignment-tensor method (*AT*: τ_D^{RDC} = 0, τ_{AT}^{RDC} = 0)[13] or the *HRS* ($K^{RDC,msy}$ = 0) method[14]. $K^{RDC,mfv}$ = 100 kJmol^{-1}Hz^{-2}, $\tau_\theta^{RDC,mfv}$ = 10 ns, in t^{mfv} = 30 ns SD simulations of the magnetic-field vector. The RDC restraint set RDC_{CAH59} contains the RDC values D_k^0 given in the fourth column of Table 1. The RDC restraint set RDC_{NH101} contains the RDC values D_k^0 given in the second column of Table 2. The RDC restraint set $RDC_{NCAH160}$ is obtained by combining the sets of RDC restraints RDC_{CAH59} and RDC_{NH101}. The values for the RDCs that are *not* part of the (sub)set of RDC restraints applied, are in bold. *RMSD*: Root-mean-square difference (RMSD) between calculated D_k and D_k^0 RDC values calculated over all, *mfv*-restrained and unrestrained, RDCs. *rRMSD*: RMSD-values calculated over the particular (sub)set of *mfv*-restrained RDCs. *urRMSD*: RMSD-values calculated over the unrestrained RDCs. Deviations of RDC-values $D_{k_1k_2}$ (*AT*) or averaged $< D_{k_1k_2} >_{t^{mfv}}$ (*HRS*) from the D_k^0 values larger than 3 Hz are in red. N_{dev}: Number of such deviations. $N_{dev,s}$: Number of RDCs for which the calculated D_k and the D_k^0 values have a different sign. These RDC values are in italics.

Residue	Experimental value (Hz)	*AT*			*HRS*		
		CAH59	*NH101*	*NCAH160*	*CAH59*	*NH101*	*NCAH160*
Gly 4	-0.55	**-0.6**	**-0.5**	**-0.6**	**-0.7**	**-0.4**	**-0.5**
Glu 7	0.20	**0.2**	**0.0**	**0.1**	**0.2**	**0.0**	**0.2**
Leu 8	0.67	**0.4**	**0.6**	**0.5**	**0.5**	**0.6**	**0.5**
Ala 9	-0.38	**-0.0**	**-0.2**	**-0.1**	**-0.1**	**-0.2**	**-0.1**
Ala 10	0.42	**0.6**	**0.7**	**0.6**	**0.7**	**0.7**	**0.6**
Ala 11	-0.95	**-0.8**	**-0.8**	**-0.8**	**-0.8**	**-0.7**	**-0.7**
Met 12	2.41	**1.8**	**1.6**	**1.7**	**1.8**	**1.5**	**1.7**
Lys 13	-0.78	**-0.8**	**-0.7**	**-0.8**	**-0.8**	**-0.6**	**-0.8**
Arg 14	1.07	**0.4**	**0.3**	**0.3**	**0.4**	**0.3**	**0.4**
Gly 16	-0.95	**-1.0**	**-0.9**	**-0.9**	**-1.0**	**-1.0**	**-1.0**
Trp 28	1.37	**1.6**	**1.4**	**1.5**	**1.6**	**1.3**	**1.5**
Ala 31	-0.23	**-0.2**	**-0.3**	**-0.3**	**-0.2**	**-0.3**	**-0.2**
Ala 32	0.38	**0.6**	**0.7**	**0.7**	**0.7**	**0.7**	**0.7**
Lys 33	-0.49	**-0.2**	**-0.4**	**-0.3**	**-0.3**	**-0.3**	**-0.3**

Asn 37	-1.12	**-0.9**	**-0.9**	**-0.9**	**-1.0**	**-1.0**	**-1.0**
Phe 38	1.72	**1.6**	**1.5**	**1.6**	**1.7**	**1.5**	**1.6**
Asn 39	-0.78	**-0.8**	**-0.8**	**-0.8**	**-0.8**	**-0.7**	**-0.7**
Thr 40	-1.41	**-1.0**	**-0.9**	**-0.9**	**-1.0**	**-0.9**	**-1.0**
Gln 41	-0.26	**-0.5**	**-0.3**	**-0.4**	**-0.5**	**-0.2**	**-0.4**
Thr 47	-1.13	**-0.8**	**-0.7**	**-0.7**	**-0.8**	**-0.7**	**-0.8**
Arg 61	1.21	**1.2**	**1.3**	**1.3**	**1.3**	**1.3**	**1.3**
Arg 68	-0.64	**-0.9**	**-0.7**	**-0.8**	**-0.8**	**-0.7**	**-0.9**
Leu 75	-0.57	**-0.4**	**-0.5**	**-0.4**	**-0.4**	**-0.5**	**-0.5**
Cys 76	1.35	**1.5**	**1.5**	**1.6**	**1.6**	**1.5**	**1.6**
Asn 77	-0.81	**-1.0**	**-0.9**	**-1.0**	**-1.1**	**-1.0**	**-1.0**
Ser 81	-1.23	**-1.0**	**-0.9**	**-0.9**	**-1.0**	**-0.9**	**-1.0**
Leu 83	-0.89	**-0.7**	**-0.5**	**-0.6**	**-0.7**	**-0.4**	**-0.6**
Leu 84	-0.63	**-0.8**	**-0.7**	**-0.7**	**-0.8**	**-0.7**	**-0.8**
Ala 90	-0.78	**-0.4**	**-0.4**	**-0.4**	**-0.5**	**-0.5**	**-0.4**
Asn 93	-0.28	**-0.5**	**-0.4**	**-0.4**	**-0.4**	**-0.4**	**-0.5**
Ala 95	-0.40	**-0.6**	**-0.6**	**-0.6**	**-0.6**	**-0.6**	**-0.5**
Lys 96	1.14	**0.9**	**1.1**	**1.0**	**1.0**	**1.1**	**1.0**
Lys 97	-1.40	**-0.9**	**-0.9**	**-0.9**	**-1.0**	**-1.0**	**-1.0**
Gly 104	0.60	**0.6**	**0.5**	**0.6**	**0.6**	**0.4**	**0.6**
Met 105	-0.05	***0.2***	**-0.0**	***0.1***	***0.2***	**-0.0**	***0.1***
Ala 110	-1.01	**-1.0**	**-1.0**	**-1.0**	**-1.1**	**-1.0**	**-1.0**
Trp 111	1.13	**1.4**	**1.1**	**1.2**	**1.4**	**1.0**	**1.3**
Asn 113	-0.28	**-0.2**	**-0.3**	**-0.2**	**-0.2**	**-0.3**	**-0.1**
Arg 114	-1.14	**-0.8**	**-0.8**	**-0.8**	**-0.9**	**-0.9**	**-0.9**
Cys 115	0.97	**1.0**	**0.7**	**0.8**	**1.0**	**0.7**	**0.9**
Gln 121	-0.78	**-0.9**	**-0.8**	**-0.9**	**-0.9**	**-0.9**	**-0.9**
Ala 122	1.68	**1.1**	**1.0**	**1.1**	**1.1**	**0.9**	**1.1**
Arg 125	1.26	**1.0**	**0.8**	**0.9**	**1.1**	**0.8**	**1.0**
Gly 126	0.52	**0.1**	***-0.0***	***-0.0***	***-0.0***	***-0.2***	***-0.1***
Cys 127	-0.67	**-0.7**	**-0.7**	**-0.7**	**-0.7**	**-0.6**	**-0.7**
RMSD		**0.3**	**0.3**	**0.3**	**0.3**	**0.3**	**0.3**
rRMSD		-	-	-	-	-	-
urRMSD		**0.3**	**0.3**	**0.3**	**0.3**	**0.3**	**0.3**
N_{dev}		**0**	**0**	**0**	**0**	**0**	**0**
$N_{dev,s}$		**1**	**1**	**2**	**2**	**1**	**2**

Table S15. $^{13}C^{\alpha}$-$^{1}H^{\alpha}$ RDC values (38, 39) in Hz for HEWL, as obtained from NMR measurements at 308 K and pH = 3.8 using two different pulse sequences as given in Tables 4.2 and 4.3 of Ref.[16], and as calculated using three different sets of RDC restraints (RDC_{CAH59}, RDC_{NH101}, $RDC_{NCAH160}$) for the X-ray structure ***1HF4*** by applying the alignment-tensor method (*AT*: $\tau_D^{RDC} = 0$, $\tau_{AT}^{RDC} = 0$)[13] or the *HRS* ($K^{RDC,msy} = 0$) method[14], $K^{RDC,mfv} = 100$ kJmol^{-1}Hz^{-2}, $\tau_{\theta}^{RDC,mfv} = 10$ ns, in $t^{mfv} = 30$ ns SD simulations of the magnetic-field vector. The (RDC restraint) set RDC_{CAH59} contains the RDC values D_k^0 given in the fourth column. The RDC restraint set RDC_{NH101} contains the RDC values D_k^0 given in the second column of Table 2. The RDC restraint set $RDC_{NCAH160}$ is obtained by combining the sets of RDC restraints RDC_{CAH59} and RDC_{NH101}. In case two experimental values are available, the D_k^0 RDC values used in the calculations are the average of the two experimental values. The values for the RDCs that are *not* part of the (sub)set of RDC restraints applied, are in bold. *RMSD*: Root-mean-square difference (RMSD) between calculated D_k and D_k^0 RDC values calculated over all, *mfv*-restrained and unrestrained, RDCs. *rRMSD*: RMSD-values calculated over the particular (sub)set of *mfv*-restrained RDCs. *urRMSD*: RMSD-values calculated over the unrestrained RDCs. Deviations of RDC values $D_{k_1k_2}$ (*AT*) or averaged $< D_{k_1k_2} >_{t^{mfv}}$ (*HRS*) from the D_k^0 values larger than 3 Hz are in red. N_{dev}: Number of such deviations. $N_{dev,s}$: Number of RDCs for which the calculated D_k and the D_k^0 values have a different sign. These RDC values are in italics.

Residue	Experimental value (Hz)			*AT*			*HRS*		
	Table 4.2[16]	Table 4.3[16]	D_k^0	*CAH59*	*NH101*	*NCAH160*	*CAH59*	*NH101*	*NCAH160*
Val 2	-5.30	-	-5.30	-9.0	**-3.6**	-5.6	-8.6	**-3.3**	-6.7
Phe 3	-	8.85	8.85	6.2	**7.6**	7.3	5.7	**7.9**	6.0
Cys 6	-	15.30	15.30	12.2	**12.9**	12.9	12.3	**12.3**	12.6
Glu 7	-	-6.52	-6.52	-6.0	**-4.2**	-5.2	-6.5	**-3.7**	-6.3
Leu 8	-	3.17	3.17	3.2	***-1.0***	0.9	2.7	***-1.0***	1.8
Met 12	-20.12	-22.20	-21.16	-20.7	**-21.7**	-21.5	-21.3	**-21.3**	-20.8

Lys 13	10.26	10.33	10.30	8.4	**10.1**	9.7	8.2	**9.8**	8.9
His 15	15.60	-	15.60	15.7	**13.9**	14.9	16.4	**14.1**	15.3
Leu 17	-	-10.66	-10.66	-14.0	**-8.9**	-10.8	-12.8	**-8.4**	-10.9
Asp 18	-	19.61	19.61	15.8	**15.1**	15.4	16.8	**15.2**	15.7
Tyr 23	-	13.81	13.81	12.8	**9.2**	10.9	12.4	**9.1**	11.4
Leu 25	-18.58	-	-18.58	-17.4	**-18.6**	-18.2	-17.7	**-18.2**	-17.3
Trp 28	-28.74	-28.51	-28.62	-28.6	**-24.1**	-26.2	-29.3	**-23.3**	-27.4
Val 29	5.17	-	5.17	8.6	**3.6**	5.8	7.4	**3.2**	6.3
Cys 30	9.83	8.84	9.34	3.6	**6.9**	5.8	5.5	**6.8**	6.3
Lys 33	13.60	-	13.60	12.3	**12.2**	12.6	11.8	**11.7**	12.0
Phe 34	-	15.36	15.36	14.1	**15.3**	15.0	15.9	**14.8**	15.7
Glu 35	-	-5.65	-5.65	-3.6	**-0.7**	-1.8	-4.0	**-0.1**	-3.4
Asn 39	-6.88	-4.05	-5.46	-8.0	**-3.3**	-5.0	-8.3	**-2.8**	-6.6
Thr 40	15.69	15.81	15.75	14.8	**13.8**	14.4	14.6	**12.9**	14.4
Ala 42	-	18.93	18.93	17.3	**16.7**	16.9	18.6	**16.0**	17.6
Asn 44	21.03	-	21.03	16.9	**17.0**	17.0	18.5	**16.4**	17.8
Thr 47	-	12.40	12.40	15.2	**12.9**	13.9	14.7	**11.9**	14.1
Asp 48	-17.97	-18.29	-18.13	-15.9	**-13.2**	-14.7	-16.8	**-12.6**	-16.0
Thr 51	16.96	-	16.96	16.0	**14.6**	15.4	17.1	**14.8**	15.9
Asp 52	-	-5.43	-5.43	*14.6*	***15.6***	*15.3*	*16.6*	***15.2***	*16.2*
Leu 56	-0.83	-	-0.83	-4.1	**-6.4**	-5.3	-3.9	**-6.1**	-4.2
Gln 57	6.72	-	6.72	11.2	**6.7**	8.5	9.9	**5.8**	8.9
Asn 59	17.60	-	17.60	16.5	**16.5**	16.6	17.8	**15.7**	17.2
Trp 62	-19.22	-22.42	-20.82	-25.7	**-24.5**	-25.1	-25.9	**-23.9**	-24.8
Trp 63	17.30	-	17.30	15.4	**16.2**	16.0	17.3	**15.7**	16.8
Cys 64	18.24	-	18.24	17.0	**16.3**	16.7	18.5	**16.4**	17.3
Asn 65	0.76	-	0.76	2.2	**4.5**	3.8	1.7	**4.9**	2.3
Pro 70	5.72	-	5.72	3.4	***-0.3***	1.4	3.2	***-0.2***	2.4
Ile 78	-	17.81	17.81	13.0	**9.8**	11.4	13.1	**9.9**	12.0
Ser 81	14.90	-	14.90	14.7	**14.0**	14.5	16.5	**14.2**	15.5
Leu 84	-	5.15	5.15	7.9	**3.6**	5.6	7.4	**3.6**	6.4
Ser 85	-	13.38	13.38	14.6	**11.8**	12.9	14.2	**10.7**	13.3
Ile 88	-14.50	-15.25	-14.88	-11.2	**-12.0**	-12.2	-12.3	**-12.0**	-12.6
Thr 89	-0.08	-3.00	-1.54	-3.5	**-8.4**	-6.7	-5.2	**-9.0**	-6.1
Ala 90	-	17.24	17.24	14.1	**14.8**	14.6	16.2	**14.6**	15.6
Val 92	14.69	-	14.69	14.6	**12.0**	13.3	14.8	**12.1**	13.7
Asn 93	-	10.98	10.98	0.3	***-3.6***	*-2.5*	*-1.0*	***-4.3***	*-2.1*
Lys 96	-1.72	-	-1.72	-5.3	**-9.3**	-7.7	-6.1	**-9.3**	-6.7
Ile 98	-26.52	-27.59	-27.06	-28.9	**-24.2**	-26.2	-28.9	**-23.4**	-26.8
Val 99	-	18.49	18.49	11.5	**11.4**	11.5	11.9	**11.7**	11.1
Ser 100	-8.22	-	-8.22	-2.6	**-7.8**	-5.8	-4.2	**-8.2**	-5.1
Trp 108	-10.09	-	-10.09	-7.8	**-12.4**	-10.9	-9.6	**-12.8**	-10.3
Val 109	14.56	19.08	16.82	15.0	**13.9**	14.5	14.8	**13.0**	14.5
Trp 111	-22.31	-22.56	-22.44	-20.1	**-14.4**	-16.7	-19.5	**-13.8**	-17.3
Arg 112	-	12.73	12.73	0.8	***-2.3***	*-1.5*	*-0.1*	***-2.9***	*-1.2*
Cys 115	-	-21.39	-21.39	-19.7	**-16.0**	-17.4	-18.8	**-15.4**	-17.2
Lys 116	8.87	3.30	6.08	11.3	**11.0**	11.0	11.9	**11.2**	10.9
Thr 118	12.69	-	12.69	11.9	**8.3**	10.0	11.7	**8.3**	10.6
Asp 119	17.09	15.66	16.37	16.9	**15.9**	16.3	17.8	**15.1**	16.9
Val 120	13.71	13.50	13.65	15.0	**13.5**	13.9	15.7	**12.8**	14.5

Gln 121	12.41	9.50	10.95	11.5	**9.2**	10.4	12.3	**9.4**	11.3
Ala 122	-	-30.56	-30.56	-30.8	**-28.1**	-29.6	-31.8	**-27.3**	-30.3
Cys 127	14.73	16.44	15.58	15.3	**13.9**	14.6	15.1	**13.0**	14.7
RMSD				4.3	**5.2**	4.6	4.4	**5.4**	4.5
rRMSD				4.3	-	4.6	4.4	-	4.5
urRMSD				-	**5.2**	-	-	**5.4**	-
N_{dev}				20	**22**	20	15	**26**	17
$N_{dev,s}$				1	**5**	3	3	**5**	3

Table S16. ^{15}N-^{1}H^{N} RDC values (101) in Hz for HEWL, as obtained from NMR measurements at 308 K and pH = 3.8, Table 4.1 of Ref.[16], and as calculated using three different sets of RDC restraints (*RDC*RDC_{CAH59}, RDC_{NH101}, $RDC_{NCAH160}$) for the X-ray structure ***1HF4*** by applying the alignment-tensor method (*AT*: $\tau_D^{RDC} = 0$, $\tau_{AT}^{RDC} = 0$)[13] or the *HRS* ($K^{RDC,msy} = 0$) method[14], $K^{RDC,mfv} = 100$ kJmol^{-1}Hz^{-2}, $\tau_\theta^{RDC,mfv} = 10$ ns, in $t^{mfv} = 30$ ns SD simulations of the magnetic-field vector. The RDC restraint set RDC_{CAH59} contains the RDC values D_k^0 given in the fourth column of Table 1. The RDC restraint set RDC_{NH101} contains the RDC values D_k^0 given in the second column. The RDC restraint set $RDC_{NCAH160}$ is obtained by combining the sets of RDC restraints RDC_{CAH59} and RDC_{NH101}. The values for the RDCs that are *not* part of the (sub)set of RDC restraints applied, are in bold. *RMSD*: Root-mean-square difference (RMSD) between calculated D_k and D_k^0 RDC values calculated over all, *mfv*-restrained and unrestrained, RDCs. *rRMSD*: RMSD-values calculated over the particular (sub)set of *mfv*-restrained RDCs. *urRMSD*: RMSD-values calculated over the unrestrained RDCs. Deviations of RDC values $D_{k_1k_2}$ (*AT*) or averaged $< D_{k_1k_2} >_{t^{mfv}}$ (*HRS*) from the D_k^0 values larger than 3 Hz are in red. N_{dev}: Number of such deviations. $N_{dev,s}$: Number of RDCs for which the calculated D_k and the D_k^0 values have a different sign. These RDC values are in italics.

Residue	Experimental value (Hz), target D_k^0	*AT*			*HRS*		
		CAH59	*NH101*	*NCAH160*	*CAH59*	*NH101*	*NCAH160*
Val 2	-1.78	**-2.2**	-3.8	-3.3	**-2.3**	-3.7	-2.9
Phe 3	7.52	**12.4**	9.4	10.6	**12.5**	9.0	11.3
Gly 4	-6.92	**-7.5**	-7.1	-7.3	**-7.7**	-7.2	-7.2
Arg 5	-6.47	**-7.4**	-7.8	-7.7	**-8.5**	-7.7	-8.2
Cys 6	-3.68	**-1.6**	-3.3	-2.7	**-2.6**	-3.2	-3.0
Glu 7	-8.56	**-9.1**	-9.0	-9.0	**-10.0**	-8.8	-9.5
Leu 8	-7.85	**-7.5**	-7.4	-7.6	**-8.5**	-7.4	-8.1
Ala 9	-4.27	**-3.9**	-4.8	-4.6	**-5.1**	-4.8	-5.1
Ala 11	-8.64	**-8.6**	-8.7	-8.7	**-9.6**	-8.6	-9.2
Lys 13	-3.36	**-2.8**	-4.4	-3.9	**-3.9**	-4.3	-4.2
Arg 14	-7.36	**-8.5**	-8.8	-8.7	**-9.4**	-8.5	-9.1
Gly 16	12.38	**13.1**	10.1	11.3	**13.0**	9.7	11.8

Leu 17	-4.59	**-2.9**	-4.5	-4.0	**-3.1**	-4.3	-3.7
Asn 19	-5.96	**-8.9**	-8.6	-8.7	**-9.6**	-8.5	-9.0
Tyr 20	0.61	***-6.4***	*-7.3*	*-7.0*	***-7.1***	*-7.0*	*-7.2*
Arg 21	-6.77	**-7.6**	-7.3	-7.4	**-8.1**	-7.4	-7.5
Gly 22	-2.70	**-3.2**	-3.9	-3.7	**-3.1**	-4.1	-3.1
Tyr 23	0.23	***-2.4***	*-0.8*	*-1.6*	***-2.5***	*-0.9*	*-2.1*
Ser24	-6.59	**-7.5**	-6.3	-6.9	**-7.1**	-6.2	-6.8
Gly 26	4.19	**0.6**	2.5	2.0	**1.2**	2.8	1.8
Asn 27	-5.76	**-5.9**	-4.9	-5.1	**-6.0**	-4.6	-5.3
Trp 28	5.29	**2.3**	2.9	2.9	**2.7**	3.0	3.0
Val 29	3.56	**2.0**	3.9	3.4	**2.7**	4.2	3.2
Ala 31	-3.83	**-4.6**	-3.8	-4.0	**-4.7**	-3.6	-4.1
Ala 32	4.34	**1.3**	2.1	2.1	**1.7**	2.3	2.1
Lys 33	0.35	***-2.8***	*-0.9*	*-1.5*	***-2.3***	*-0.5*	*-1.7*
Phe 34	-4.22	**-7.1**	-5.8	-6.2	**-7.1**	-5.4	-6.5
Glu 35	3.73	**3.9**	3.7	4.0	**4.3**	3.6	4.4
Ser 36	7.51	**7.9**	9.0	8.8	**8.7**	9.0	8.9
Asn 37	-7.43	**-7.6**	-7.0	-7.4	**-7.4**	-6.6	-7.3
Phe 38	-0.76	**-3.1**	-4.6	-4.1	**-3.3**	-4.5	-3.8
Asn 39	-2.69	**-3.0**	-3.9	-3.7	**-2.8**	-4.0	-3.0
Thr 40	6.48	**8.6**	5.5	6.7	**8.6**	5.2	7.4
Gln 41	6.78	**8.3**	6.5	7.1	**7.7**	6.2	6.9
Ala 42	10.72	**7.3**	7.9	7.9	**8.0**	7.9	8.1
Thr 43	-6.94	**-7.1**	-7.6	-7.5	**-8.2**	-7.5	-8.0
Asn 44	-8.88	**-8.0**	-8.2	-8.2	**-9.1**	-8.1	-8.7
Arg 45	-7.32	**-6.0**	-7.0	-6.7	**-7.0**	-6.8	-7.0
Asn 46	-4.82	**-7.8**	-7.1	-7.5	**-8.5**	-7.2	-7.9
Thr 47	4.71	**0.4**	3.1	2.1	**1.2**	3.4	1.7
Asp 48	14.01	**14.4**	14.1	14.5	**15.1**	13.9	14.7
Gly 49	1.82	***-0.6***	*-1.3*	*-1.2*	***-1.6***	*-1.5*	*-1.7*
Thr 51	-7.92	**-8.8**	-8.6	-8.7	**-9.7**	-8.6	-9.2
Gly 54	-6.58	**-6.5**	-5.1	-5.5	**-6.4**	-4.7	-5.8
Ile 55	-7.54	**-7.9**	-7.0	-7.5	**-8.0**	-7.1	-7.5
Leu 56	-0.14	**-2.1**	-0.0	-1.0	**-1.9**	-0.1	-1.5
Gln 57	-3.19	**-1.7**	-3.4	-2.8	**-1.7**	-3.3	-2.3
Ile 58	-3.83	**-0.4**	-2.1	-1.5	**-0.2**	-2.2	-0.8
Asn 59	-3.85	**-6.1**	-4.0	-4.8	**-5.6**	-3.5	-5.0
Ser 60	-7.82	**-9.0**	-8.9	-9.0	**-10.0**	-8.8	-9.4
Trp 63	-8.15	**-9.0**	-8.8	-9.0	**-10.0**	-8.8	-9.4
Asn 65	1.18	***-0.6***	*-1.4*	*-1.0*	***-0.5***	*-1.7*	*-0.5*
Asp 66	-0.81	**-0.3**	-2.0	-1.4	**-0.1**	-2.1	-0.7
Gly 67	-7.13	**-8.0**	-6.9	-7.3	**-8.1**	-6.4	-7.6
Arg 68	13.60	**9.7**	11.0	10.7	**10.6**	11.0	10.6
Thr 69	-3.43	**-7.3**	-7.0	-7.2	**-7.6**	-7.2	-7.1
Gly 71	2.44	**3.2**	5.6	4.7	**3.9**	5.7	4.2
Arg 73	-1.26	**-2.4**	-0.7	-1.5	**-2.5**	-0.8	-2.1
Asn 74	-7.40	**-0.8**	-2.1	-1.7	**-1.8**	-2.2	-2.1
Cys 76	-4.97	**-6.2**	-5.6	-5.7	**-6.5**	-5.5	-5.8
Ile 78	-1.37	**-5.0**	-5.2	-5.2	**-4.8**	-5.4	-4.7
Cys 80	16.06	**16.0**	14.6	15.3	**16.5**	14.2	15.8

Ser 81	14.37	**11.4**	11.1	11.3	**11.4**	10.8	11.0
Ala 82	7.74	**11.2**	8.4	9.5	**10.8**	8.0	9.7
Leu 83	15.17	**14.8**	13.7	14.3	**15.4**	13.3	14.8
Leu 84	5.43	**0.7**	2.3	1.6	**0.7**	2.2	1.0
Ser 86	-4.45	**-6.2**	-3.9	-4.9	**-5.6**	-3.6	-5.1
Asp 87	12.03	**4.7**	7.1	6.2	**5.5**	7.2	5.8
Thr 89	-2.93	**-2.7**	-4.1	-3.7	**-2.7**	-4.0	-3.2
Ser 91	-0.10	**-1.2**	-3.2	-2.5	**-1.5**	-3.1	-2.2
Val 92	-4.32	**-3.5**	-4.6	-4.3	**-3.4**	-4.5	-3.8
Asn 93	-6.06	**-7.0**	-6.7	-7.0	**-6.8**	-6.4	-6.8
Cys 94	-5.79	**-4.9**	-6.1	-5.7	**-5.3**	-5.8	-5.6
Ala 95	-2.42	**-0.5**	-2.4	-1.8	**-0.5**	-2.4	-1.2
Lys 96	-6.08	**-6.9**	-6.3	-6.8	**-6.6**	-6.2	-6.5
Lys 97	-7.62	**-7.9**	-7.0	-7.4	**-7.7**	-6.5	-7.5
Ile 98	-4.26	**-3.2**	-4.5	-4.2	**-3.2**	-4.4	-3.7
Ser 100	-4.10	**-6.7**	-4.5	-5.5	**-6.1**	-4.2	-5.6
Gly 102	8.96	**11.0**	9.4	10.2	**11.5**	9.0	10.9
Asn 103	-7.26	**-6.6**	-6.2	-6.5	**-6.2**	-6.1	-6.1
Gly 104	-4.67	**-3.2**	-3.4	-3.4	**-4.1**	-3.5	-4.0
Met 105	9.39	**2.9**	5.4	4.6	**3.8**	5.7	4.2
Asn 106	-7.82	**-8.8**	-8.4	-8.6	**-9.3**	-8.0	-8.9
Ala 107	-1.63	**-1.1**	-0.7	-0.7	**-1.0**	-0.7	-0.5
Trp 108	3.79	**2.0**	3.9	3.4	**2.7**	4.2	3.2
Val 109	7.54	**4.8**	7.1	6.3	**5.6**	7.2	5.8
Trp 111	9.71	**6.1**	7.0	6.6	**6.1**	6.8	6.1
Arg 112	8.40	**6.7**	8.6	7.9	**7.3**	8.5	7.5
Asn 113	1.87	***-3.1***	*-0.5*	*-1.6*	***-2.5***	*-0.3*	*-2.0*
Cys 115	14.32	**14.9**	12.5	13.6	**14.9**	12.1	13.8
Lys 116	12.40	**8.4**	9.7	9.5	**9.2**	9.8	9.4
Gly 117	-7.17	**-8.6**	-8.1	-8.4	**-9.2**	-8.2	-8.6
Val 120	-4.37	**-4.4**	-5.7	-5.3	**-4.9**	-5.5	-5.2
Gln 121	-6.83	**-7.5**	-6.2	-6.8	**-7.0**	-5.9	-6.7
Ala 122	-0.89	**-0.4**	-2.0	-1.5	**-0.2**	-2.1	-0.7
Ile 124	-5.73	**-6.9**	-4.8	-5.8	**-6.3**	-4.5	-5.9
Arg 125	1.26	**2.9**	1.8	2.3	**3.2**	1.5	3.0
Gly 126	-2.46	**-1.7**	*0.6*	-0.1	**-1.0**	*1.1*	-0.4
Cys 127	-3.28	**-4.5**	-5.1	-5.0	**-4.3**	-5.0	-4.5
Arg 128	-4.94	**-4.5**	-2.3	-3.0	**-3.9**	-1.8	-3.3
Leu 129	1.83	***-3.3***	*-0.8*	*-1.9*	***-2.8***	*-0.7*	*-2.3*
RMSD		**2.4**	1.9	2.0	**2.4**	2.0	2.1
rRMSD		-	1.9	2.0	-	2.0	2.1
urRMSD		**2.4**	-	-	**2.4**	-	-
N_{dev}		**20**	12	14	**18**	13	17
$N_{dev,s}$		**7**	8	7	**6**	7	7

Table S17. $^{13}C^{\alpha}$-$^{13}C'$ RDC values (97) in Hz for HEWL, as obtained from NMR measurements at 308 K and pH = 3.8, Table 4.4 of Ref.[16], and as calculated using three different sets of RDC restraints (RDC_{CAH59}, RDC_{NH101}, $RDC_{NCAH160}$) for the X-ray structure ***1HF4*** by applying the alignment-tensor method (*AT*: $\tau_D^{RDC} = 0$, $\tau_{AT}^{RDC} = 0$)[13] or the *HRS* ($K^{RDC,msy} = 0$) method[14]. $K^{RDC,mfv} = 100$ kJmol^{-1}Hz^{-2}, $\tau_\theta^{RDC,mfv} = 10$ ns, in $t^{mfv} = 30$ ns SD simulations of the magnetic-field vector. The RDC restraint set RDC_{CAH59} contains the RDC values D_k^0 given in the fourth column of Table 1. The RDC restraint set RDC_{NH101} contains the RDC values D_k^0 given in the second column of Table 2. The RDC restraint set $RDC_{NCAH160}$ is obtained by combining the sets of RDC restraints RDC_{CAH59} and RDC_{NH101}. The values for the RDCs that are *not* part of the (sub)set of RDC restraints applied, are in bold. *RMSD*: Root-mean-square difference (RMSD) between calculated D_k and D_k^0 RDC values calculated over all, *mfv*-restrained and unrestrained, RDCs. *rRMSD*: RMSD-values calculated over the particular (sub)set of *mfv*-restrained RDCs. *urRMSD*: RMSD-values calculated over the unrestrained RDCs. Deviations of RDC-values $D_{k_1k_2}$ (*AT*) or averaged $< D_{k_1k_2} >_{t^{mfv}}$ (*HRS*) from the D_k^0 values larger than 3 Hz are in red. N_{dev}: Number of such deviations. $N_{dev,s}$: Number of RDCs for which the calculated D_k and D_k^0 values have a different sign. These RDC values are in italics.

Residue	Experimental value (Hz)	*AT*			*HRS*		
		CAH59	*NH101*	*NCAH160*	*CAH59*	*NH101*	*NCAH160*
Lys 1	0.97	**1.1**	**0.7**	**0.9**	**1.0**	**0.7**	**0.9**
Val 2	-2.11	**-1.6**	**-1.7**	**-1.7**	**-1.8**	**-1.7**	**-1.8**
Phe 3	-0.50	**-0.5**	**-0.7**	**-0.6**	**-0.5**	**-0.7**	**-0.5**
Gly 4	1.95	**1.4**	**1.2**	**1.3**	**1.4**	**1.1**	**1.3**
Glu 7	-1.56	**-1.5**	**-1.0**	**-1.2**	**-1.4**	**-1.0**	**-1.3**
Leu 8	1.19	**1.3**	**1.0**	**1.1**	**1.2**	**0.9**	**1.1**
Ala 9	1.19	**1.2**	**1.2**	**1.2**	**1.3**	**1.2**	**1.2**
Ala 10	-1.30	**-1.3**	**-1.3**	**-1.3**	**-1.2**	**-1.3**	**-1.2**
Ala 11	0.13	**0.1**	**0.5**	**0.4**	**0.2**	**0.5**	**0.3**
Met 12	0.83	**0.7**	**0.5**	**0.5**	**0.7**	**0.4**	**0.5**
Lys 13	1.51	**1.4**	**1.2**	**1.3**	**1.4**	**1.2**	**1.3**
Arg 14	-1.94	**-2.1**	**-1.7**	**-1.8**	**-2.0**	**-1.6**	**-1.9**
His 15	1.01	**1.2**	**1.2**	**1.2**	**1.2**	**1.1**	**1.2**
Gly 16	0.55	**0.9**	**0.9**	**0.9**	**0.8**	**0.9**	**0.8**

Asp 18	1.71	1.3	1.4	1.4	1.4	1.3	1.4
Asn 19	-1.81	-1.7	-1.5	-1.5	-1.6	-1.4	-1.5
Tyr 23	-0.67	-1.3	-0.8	-1.0	-1.3	-0.7	-1.1
Ser24	1.34	1.3	1.0	1.1	1.2	0.9	1.2
Leu 25	0.76	1.5	1.5	1.5	1.7	1.5	1.6
Asn 27	-0.39	*0.1*	-0.4	-0.2	-0.0	-0.4	-0.1
Trp 28	1.41	1.2	1.3	1.3	1.3	1.3	1.3
Cys 30	-2.86	-2.6	-2.5	-2.6	-2.7	-2.5	-2.6
Ala 31	1.21	1.3	1.0	1.1	1.2	0.9	1.1
Ala 32	1.67	1.3	1.4	1.4	1.5	1.3	1.4
Lys 33	-0.74	-0.8	-0.6	-0.7	-0.8	-0.6	-0.8
Phe 34	-2.53	-2.4	-2.4	-2.4	-2.4	-2.4	-2.4
Ser 36	-1.04	-1.3	-0.9	-1.0	-1.2	-0.8	-1.1
Asn 37	0.81	0.6	0.7	0.6	0.5	0.7	0.5
Phe 38	0.83	0.6	0.2	0.4	0.5	0.1	0.4
Asn 39	-0.43	-0.5	-0.4	-0.4	-0.4	-0.4	-0.4
Thr 40	-2.42	-2.1	-2.2	-2.2	-2.2	-2.2	-2.2
Gln 41	0.14	0.3	0.5	0.4	0.3	0.5	0.3
Ala 42	-2.63	-2.3	-1.9	-2.1	-2.3	-1.8	-2.1
Asn 44	-1.67	-2.6	-2.2	-2.4	-2.6	-2.1	-2.4
Asn 46	-2.51	-2.3	-2.1	-2.2	-2.4	-2.1	-2.3
Thr 47	-0.95	-1.0	-0.7	-0.8	-0.9	-0.7	-0.8
Asp 48	1.48	1.1	0.8	1.0	1.1	0.8	1.0
Gly 49	-0.60	-0.8	-0.6	-0.7	-0.9	-0.6	-0.8
Ser 50	-1.22	-1.3	-0.8	-1.0	-1.2	-0.8	-1.0
Thr 51	-1.57	-1.1	-1.2	-1.2	-1.3	-1.2	-1.3
Tyr 53	-1.29	-2.3	-2.2	-2.3	-2.4	-2.2	-2.4
Leu 56	-2.63	-2.2	-1.9	-2.1	-2.3	-1.9	-2.2
Gln 57	-0.66	-0.7	-0.3	-0.4	-0.5	-0.2	-0.4
Asn 59	-1.71	-0.4	-0.6	-0.5	-0.4	-0.6	-0.4
Ser 60	-2.19	-1.5	-1.0	-1.2	-1.4	-0.9	-1.2
Arg 61	1.21	1.6	1.6	1.6	1.7	1.5	1.6
Cys 64	-2.38	-1.5	-1.7	-1.7	-1.7	-1.7	-1.7
Asn 65	0.92	1.4	1.4	1.4	1.5	1.4	1.5
Asp 66	-2.07	-1.9	-2.1	-2.1	-2.0	-2.1	-2.0
Gly 67	0.08	0.5	0.5	0.5	0.5	0.5	0.4
Arg 68	1.42	1.3	1.1	1.2	1.2	1.1	1.2
Gly 71	-2.77	-2.6	-2.4	-2.6	-2.7	-2.4	-2.6
Asn 74	0.44	0.4	0.1	0.2	0.3	0.1	0.2
Leu 75	-2.30	-1.7	-1.3	-1.5	-1.7	-1.2	-1.6
Cys 76	1.80	1.2	0.8	1.0	1.1	0.8	1.0
Asn 77	0.62	0.4	0.7	0.6	0.5	0.7	0.6
Pro 79	0.26	*-0.4*	*-0.5*	*-0.5*	*-0.4*	-0.5	-0.4
Cys 80	-0.23	-0.5	-0.0	-0.2	-0.4	-0.6	-0.3
Ser 81	-1.71	-1.5	-1.4	-1.5	-1.6	-1.4	-1.6
Ala 82	-0.80	-0.5	-0.9	-0.7	-0.6	-0.9	-0.6
Leu 83	1.12	0.6	0.8	0.8	0.8	0.8	0.8
Leu 84	-0.72	-1.1	-0.6	-0.8	-1.1	-0.6	-0.9
Ser 85	-2.60	-2.2	-2.3	-2.3	-2.3	-2.2	-2.2
Ile 88	2.38	0.9	1.1	1.0	1.1	1.1	1.1

Thr 89	-1.36	**-2.2**	**-1.7**	**-1.9**	**-2.2**	**-1.6**	**-2.0**
Ala 90	1.77	**1.3**	**1.1**	**1.2**	**1.2**	**1.1**	**1.2**
Ser 91	1.06	**0.7**	**0.4**	**0.5**	**0.6**	**0.3**	**0.5**
Val 92	1.08	**0.2**	**0.5**	**0.4**	**0.4**	**0.5**	**0.5**
Asn 93	-1.11	**-1.5**	**-1.1**	**-1.3**	**-1.6**	**-1.0**	**-1.4**
Cys 94	0.75	**1.1**	**0.7**	**0.9**	**1.0**	**0.7**	**0.9**
Ala 95	1.20	**1.4**	**1.2**	**1.2**	**1.4**	**1.1**	**1.3**
Lys 96	-1.79	**-1.6**	**-1.1**	**-1.3**	**-1.6**	**-1.0**	**-1.4**
Lys 97	0.79	**1.0**	**1.0**	**1.1**	**1.1**	**1.1**	**1.0**
Ile 98	-1.68	***0.4***	**-0.1**	***0.1***	***0.2***	**-0.2**	***0.1***
Val 99	1.81	**1.3**	**1.4**	**1.4**	**1.4**	**1.3**	**1.4**
Ser 100	-2.11	**-2.5**	**-2.1**	**-2.3**	**-2.6**	**-2.0**	**-2.4**
Gly 102	0.52	**1.1**	**1.0**	**1.1**	**1.3**	**1.0**	**1.2**
Gly 104	1.94	**1.5**	**1.5**	**1.5**	**1.6**	**1.4**	**1.6**
Met 105	0.41	**0.3**	**0.3**	**0.3**	**0.3**	**0.4**	**0.2**
Asn 106	-0.99	**-0.7**	**-1.1**	**-1.0**	**-0.9**	**-1.2**	**-0.9**
Ala 107	1.44	**1.3**	**1.4**	**1.4**	**1.4**	**1.3**	**1.4**
Ala 110	-1.26	**-0.9**	**-1.2**	**-1.1**	**-1.0**	**-1.2**	**-1.1**
Trp 111	1.12	**1.3**	**1.1**	**1.2**	**1.2**	**1.1**	**1.2**
Asn 113	-2.61	**-2.4**	**-2.0**	**-2.2**	**-2.4**	**-2.0**	**-2.2**
Arg 114	0.28	***-0.7***	***-0.7***	***-0.7***	***-0.7***	***-0.7***	***-0.8***
Cys 115	1.25	**1.5**	**1.3**	**1.4**	**1.5**	**1.2**	**1.4**
Lys 116	-0.35	***0.0***	**-0.4**	**-0.2**	**-0.0**	**-0.4**	**-0.1**
Thr 118	-0.81	**-0.9**	**-0.7**	**-0.8**	**-0.9**	**-0.6**	**-0.9**
Val 120	-0.92	**-1.1**	**-0.8**	**-0.9**	**-1.2**	**-0.7**	**-1.1**
Gln 121	1.11	**0.8**	**1.0**	**0.9**	**0.9**	**1.0**	**1.0**
Ala 122	0.73	**0.8**	**0.4**	**0.6**	**0.7**	**0.3**	**0.6**
Trp 123	0.43	**0.5**	**0.6**	**0.6**	**0.5**	**0.7**	**0.5**
Ile 124	-3.36	**-2.6**	**-2.2**	**-2.4**	**-2.6**	**-2.2**	**-2.4**
Arg 125	0.76	**0.9**	**1.0**	**1.0**	**0.8**	**0.9**	**0.9**
Gly 126	0.95	**1.0**	**0.7**	**0.8**	**1.0**	**0.7**	**0.9**
Cys 127	0.43	**0.3**	**0.3**	**0.2**	**0.3**	**0.3**	**0.2**
Arg 128	-0.62	**-0.6**	**-0.5**	**-0.5**	**-0.5**	**-0.5**	**-0.4**
RMSD		**0.5**	**0.5**	**0.5**	**0.5**	**0.5**	**0.5**
rRMSD		-	-	-	-	-	-
urRMSD		**0.5**	**0.5**	**0.5**	**0.5**	**0.5**	**0.5**
N_{dev}		**0**	**0**	**0**	**0**	**0**	**0**
$N_{dev,s}$		**5**	**2**	**3**	**3**	**1**	**2**

Table S18. $^{13}C'$-^{15}N RDC values (45) in Hz for HEWL, as obtained from NMR measurements at 308 K and pH = 3.8, Table 4.5 of Ref.[16], and as calculated using three different sets of RDC restraints (RDC_{CAH59}, RDC_{NH101}, $RDC_{NCAH160}$) for the X-ray structure ***1HF4*** by applying the alignment-tensor method (*AT*: $\tau_D^{RDC} = 0$, $\tau_{AT}^{RDC} = 0$)[13] or the *HRS* ($K^{RDC,msy} = 0$) method[14]. $K^{RDC,mfv} = 100$ kJmol^{-1}Hz^{-2}, $\tau_\theta^{RDC,mfv} = 10$ ns, in $t^{mfv} = 30$ ns SD simulations of the magnetic-field vector. The RDC restraint set RDC_{CAH59} contains the RDC values D_k^0 given in the fourth column of Table 1. The RDC restraint set RDC_{NH101} contains the RDC values D_k^0 given in the second column of Table 2. The RDC restraint set $RDC_{NCAH160}$ is obtained by combining the sets of RDC restraints RDC_{CAH59} and RDC_{NH101}. The values for the RDCs that are *not* part of the (sub)set of RDC restraints applied, are in bold. *RMSD*: Root-mean-square difference (RMSD) between calculated D_k and D_k^0 RDC values calculated over all, *mfv*-restrained and unrestrained, RDCs. *rRMSD*: RMSD-values calculated over the particular (sub)set of *mfv*-restrained RDCs. *urRMSD*: RMSD-values calculated over the unrestrained RDCs. Deviations of RDC-values $D_{k_1k_2}$ (*AT*) or averaged $< D_{k_1k_2} >_{t^{mfv}}$ (*HRS*) from the D_k^0 values larger than 3 Hz are in red. N_{dev}: Number of such deviations. $N_{dev,s}$: Number of RDCs for which the calculated D_k and the D_k^0 values have a different sign. These RDC values are in italics.

Residue	Experimental value (Hz)	*AT*			*HRS*		
		CAH59	*NH101*	*NCAH160*	*CAH59*	*NH101*	*NCAH160*
Gly 4	-0.55	**-0.8**	**-0.5**	**-0.6**	**-0.7**	**-0.5**	**-0.7**
Glu 7	0.20	**0.1**	***-0.0***	**0.0**	**0.1**	***-0.1***	**0.1**
Leu 8	0.67	**0.5**	**0.8**	**0.7**	**0.6**	**0.8**	**0.6**
Ala 9	-0.38	***0.0***	**-0.2**	**-0.1**	***0.0***	**-0.2**	**-0.0**
Ala 10	0.42	**0.4**	**0.5**	**0.5**	**0.4**	**0.6**	**0.5**
Ala 11	-0.95	**-0.8**	**-0.7**	**-0.7**	**-0.8**	**-0.7**	**-0.7**
Met 12	2.41	**1.8**	**1.6**	**1.7**	**1.8**	**1.6**	**1.7**
Lys 13	-0.78	**-0.8**	**-0.7**	**-0.7**	**-0.8**	**-0.6**	**-0.7**
Arg 14	1.07	**0.5**	**0.1**	**0.5**	**0.5**	**0.4**	**0.5**
Gly 16	-0.95	**-0.9**	**-0.9**	**-0.9**	**-1.0**	**-0.9**	**-1.0**
Trp 28	1.37	**1.6**	**1.4**	**1.5**	**1.6**	**1.4**	**1.5**
Ala 31	-0.23	**-0.1**	**-0.2**	**-0.2**	**-0.1**	**-0.2**	**-0.1**
Ala 32	0.38	**0.7**	**0.9**	**0.8**	**0.7**	**0.8**	**0.7**
Lys 33	-0.49	**-0.3**	**-0.4**	**-0.4**	**-0.3**	**-0.4**	**-0.3**

Asn 37	-1.12	**-1.0**	**-0.9**	**-1.0**	**-1.1**	**-0.9**	**-1.0**
Phe 38	1.72	**1.2**	**1.1**	**1.2**	**1.2**	**1.1**	**1.2**
Asn 39	-0.78	**-0.8**	**-0.7**	**-0.8**	**-0.8**	**-0.7**	**-0.7**
Thr 40	-1.41	**-1.0**	**-0.9**	**-0.9**	**-1.0**	**-0.9**	**-1.0**
Gln 41	-0.26	**-0.5**	**-0.2**	**-0.4**	**-0.4**	**-0.2**	**-0.4**
Thr 47	-1.13	**-0.8**	**-0.8**	**-0.8**	**-0.9**	**-0.7**	**-0.8**
Arg 61	1.21	**1.2**	**1.2**	**1.2**	**1.3**	**1.2**	**1.3**
Arg 68	-0.64	**-0.8**	**-0.7**	**-0.8**	**-0.9**	**-0.7**	**-0.8**
Leu 75	-0.57	**-0.2**	**-0.4**	**-0.4**	**-0.3**	**-0.4**	**-0.4**
Cys 76	1.35	**1.5**	**1.6**	**1.6**	**1.6**	**1.5**	**1.6**
Asn 77	-0.81	**-0.9**	**-0.9**	**-1.0**	**-1.1**	**-0.9**	**-1.0**
Ser 81	-1.23	**-1.0**	**-1.0**	**-1.0**	**-1.1**	**-0.9**	**-1.0**
Leu 83	-0.89	**-0.8**	**-0.6**	**-0.7**	**-0.7**	**-0.5**	**-0.7**
Leu 84	-0.63	**-0.8**	**-0.7**	**-0.7**	**-0.8**	**-0.7**	**-0.8**
Ala 90	-0.78	**-0.4**	**-0.4**	**-0.4**	**-0.5**	**-0.4**	**-0.5**
Asn 93	-0.28	**-0.4**	**-0.3**	**-0.3**	**-0.4**	**-0.3**	**-0.3**
Ala 95	-0.40	**-0.6**	**-0.6**	**-0.6**	**-0.7**	**-0.6**	**-0.6**
Lys 96	1.14	**1.1**	**1.2**	**1.2**	**1.1**	**1.2**	**1.1**
Lys 97	-1.40	**-0.9**	**-0.9**	**-0.9**	**-1.0**	**-0.9**	**-1.0**
Gly 104	0.60	**0.8**	**0.7**	**0.7**	**0.7**	**0.7**	**0.7**
Met 105	-0.05	***0.0***	**-0.2**	**-0.1**	***0.0***	**-0.2**	**-0.0**
Ala 110	-1.01	**-1.0**	**-0.9**	**-1.0**	**-1.1**	**-0.9**	**-1.0**
Trp 111	1.13	**1.4**	**1.1**	**1.2**	**1.4**	**1.0**	**1.3**
Asn 113	-0.28	**-0.1**	**-0.2**	**-0.2**	**-0.1**	**-0.2**	**-0.1**
Arg 114	-1.14	**-0.8**	**-0.8**	**-0.8**	**-0.9**	**-0.8**	**-0.9**
Cys 115	0.97	**1.3**	**0.9**	**1.1**	**1.3**	**0.9**	**1.1**
Gln 121	-0.78	**-0.9**	**-0.8**	**-0.9**	**-0.9**	**-0.8**	**-0.9**
Ala 122	1.68	**1.2**	**1.1**	**1.2**	**1.2**	**1.1**	**1.2**
Arg 125	1.26	**1.4**	**1.2**	**1.3**	**1.4**	**1.1**	**1.3**
Gly 126	0.52	**0.5**	**0.2**	**0.3**	**0.4**	**0.2**	**0.3**
Cys 127	-0.67	**-0.8**	**-0.7**	**-0.7**	**-0.7**	**-0.7**	**-0.7**
RMSD		**0.3**	**0.3**	**0.3**	**0.3**	**0.3**	**0.3**
rRMSD		-	-	-	-	-	-
urRMSD		**0.3**	**0.3**	**0.3**	**0.3**	**0.3**	**0.3**
N_{dev}		**0**	**0**	**0**	**0**	**0**	**0**
$N_{dev,s}$		**2**	**1**	**0**	**2**	**1**	**0**

Table S19. Largest variation in ^{15}N-$^{1}H^{N}$ RDC values (101) in Hz for HEWL between three different sets of RDC restraints (CAH59, NH101, NCAH160) using the alignment-tensor (*AT*) method or the magnetic-field rotation (*HRS*) method, for five X-ray structures, *4LZT*, *2VB1*, *1IEE*, *1AKI* and *1HF4*, obtained from the data in Tables 2, S2, S6, S10 and S14, respectively. Variations larger than 3 Hz are in red. N_{var}: Number of such variations.

Residue	*4LZT*		*2VB1*		*1IEE*		*1AKI*		*1HF4*	
	AT	*HRS*	*AT*	*HRS*	*AT*	*HRS*	*AT*	*HRS*	*AT*	*HRS*
Val 2	1.1	0.9	1.1	0.7	0.5	0.4	1.0	0.5	1.6	1.4
Phe 3	3.1	3.5	3.0	2.7	3.1	2.6	2.5	3.0	3.0	3.5
Gly 4	0.7	0.6	0.7	0.9	0.3	0.6	0.2	0.6	0.4	0.5
Arg 5	0.5	0.4	0.3	0.8	1.5	0.8	0.1	0.2	0.4	0.8
Cys 6	0.8	0.7	0.5	0.8	1.7	0.9	0.4	0.3	1.7	0.6
Glu 7	0.5	0.3	0.4	0.7	0.2	0.6	0.5	0.6	0.1	1.2
Leu 8	0.4	0.7	0.4	0.7	0.3	0.7	0.3	0.4	0.2	1.1
Ala 9	0.7	0.5	0.6	0.8	1.3	0.7	0.1	0.1	0.9	0.3
Ala 11	0.4	0.4	0.3	0.7	0.2	0.6	0.4	0.5	0.1	1.0
Lys 13	1.1	0.9	1.0	0.9	1.6	0.9	0.7	0.5	1.6	0.4
Arg 14	0.1	0.3	0.0	0.7	0.1	0.5	0.2	0.6	0.3	0.9
Gly 16	2.9	2.9	3.0	2.9	3.1	2.4	2.0	2.6	3.0	3.3
Leu 17	0.4	0.2	0.4	0.3	0.6	0.4	0.8	0.4	1.6	1.2
Asn 19	0.6	0.4	0.5	0.6	0.1	0.7	0.5	0.5	0.3	1.1
Tyr 20	0.1	0.2	0.1	0.9	0.2	0.2	0.3	0.6	0.9	0.2
Arg 21	0.5	0.2	0.5	0.7	0.3	0.8	0.1	0.3	0.3	0.7
Gly 22	0.4	1.0	0.4	1.2	0.1	0.1	0.9	0.7	0.7	1.0
Tyr 23	1.5	2.5	1.8	1.7	0.6	1.0	1.1	1.6	1.6	1.6
Ser24	1.7	2.3	1.9	1.8	0.9	1.3	0.7	1.6	1.2	0.9
Gly 26	1.4	1.4	1.2	1.0	2.4	0.8	1.5	1.3	1.9	1.6
Asn 27	1.0	0.9	0.8	0.9	1.8	0.9	1.1	1.0	1.0	1.4
Trp 28	0.2	1.2	0.3	0.8	1.1	0.4	0.5	0.3	0.6	0.3
Val 29	1.1	1.0	0.7	0.5	2.0	0.9	1.3	1.1	1.9	1.5
Ala 31	0.8	0.5	0.4	0.6	1.5	0.7	0.9	0.7	0.8	1.1
Ala 32	0.2	1.0	0.2	0.8	1.3	0.5	0.6	0.5	0.8	0.6
Lys 33	1.5	1.4	1.2	1.0	2.5	1.3	1.7	1.8	1.9	1.8
Phe 34	1.2	1.1	1.0	1.3	1.9	1.1	1.2	1.4	1.3	1.7
Glu 35	0.3	1.6	0.5	1.1	0.7	0.4	0.3	0.8	0.3	0.8
Ser 36	0.2	0.8	0.5	0.9	1.2	0.5	0.7	0.4	1.1	0.3
Asn 37	1.5	2.1	1.8	1.9	1.1	1.0	0.5	1.4	0.6	0.8
Phe 38	2.4	1.3	1.0	0.5	1.7	1.2	2.4	1.1	1.5	1.2
Asn 39	0.5	0.9	0.1	1.1	0.5	0.3	1.3	0.9	0.9	1.2
Thr 40	2.9	3.3	2.8	2.6	2.7	2.3	2.4	2.8	3.1	3.4
Gln 41	1.8	1.6	1.9	1.9	2.6	1.8	0.5	0.9	1.8	1.5
Ala 42	0.3	1.1	0.7	1.1	0.9	0.6	0.4	0.5	0.6	0.2
Thr 43	0.1	0.2	0.1	0.6	0.3	0.5	0.2	0.4	0.5	0.7
Asn 44	0.2	0.3	0.1	0.6	0.4	0.5	0.2	0.3	0.2	1.0
Arg 45	0.6	0.5	0.5	0.5	0.1	0.5	0.1	0.1	1.0	0.2

Asn 46	0.7	0.7	0.7	0.8	0.2	1.0	0.7	1.0	0.7	1.3
Thr 47	1.9	3.0	1.9	1.9	1.6	1.0	1.6	1.5	2.7	2.2
Asp 48	1.6	2.2	1.7	2.0	0.3	1.0	0.3	0.6	0.4	1.2
Gly 49	0.9	0.8	0.8	0.9	1.7	1.0	0.1	0.4	0.7	0.2
Thr 51	0.4	0.4	0.3	0.6	0.2	0.7	0.3	0.4	0.2	1.1
Gly 54	1.1	1.1	0.9	1.0	1.6	0.9	1.2	1.3	1.4	1.7
Ile 55	1.2	1.3	1.2	1.2	0.7	1.0	0.4	0.9	0.9	0.9
Leu 56	1.6	2.7	1.8	1.7	0.8	1.1	1.2	1.6	2.1	1.8
Gln 57	0.2	0.1	0.1	0.4	0.6	0.3	1.0	0.4	1.7	1.6
Ile 58	0.4	0.6	0.2	0.8	0.5	0.3	1.0	0.4	1.7	2.0
Asn 59	2.1	2.5	2.2	2.3	2.2	1.4	1.9	2.4	2.1	2.1
Ser 60	0.5	0.4	0.5	0.6	0.3	0.6	0.4	0.4	0.1	1.2
Trp 63	0.6	0.5	0.5	0.7	0.5	0.6	0.5	0.6	0.2	1.2
Asn 65	0.9	2.1	1.0	1.8	0.7	0.7	1.1	1.3	0.8	1.2
Asp 66	0.4	0.7	0.2	0.7	1.1	0.9	1.1	0.6	1.7	2.0
Gly 67	1.7	1.8	1.7	1.9	1.8	1.1	1.6	2.0	1.1	1.7
Arg 68	0.4	0.7	0.2	0.7	0.5	0.6	0.3	0.6	1.3	0.4
Thr 69	0.2	0.8	0.4	0.8	0.4	0.8	0.2	0.5	0.3	0.5
Gly 71	0.3	1.0	0.5	1.0	1.6	1.5	1.0	1.0	2.4	1.8
Arg 73	0.9	0.7	0.8	1.1	0.7	0.4	0.6	1.0	1.7	1.7
Asn 74	0.5	0.4	0.5	0.6	0.2	0.7	0.5	0.5	1.3	0.4
Cys 76	0.5	0.5	0.2	0.6	0.7	0.4	0.8	0.6	0.6	1.0
Ile 78	0.1	0.7	0.2	1.1	0.6	0.6	0.6	0.6	0.2	0.7
Cys 80	2.1	2.9	2.5	2.6	1.6	1.7	1.3	2.4	1.4	2.3
Ser 81	1.2	1.1	1.3	1.8	1.2	1.0	0.7	1.5	0.3	0.6
Ala 82	2.9	2.8	2.8	2.6	3.1	2.4	2.2	2.7	2.8	2.8
Leu 83	2.3	3.2	2.8	2.9	1.1	1.5	1.3	2.3	1.1	2.1
Leu 84	0.7	1.8	0.8	0.9	0.4	0.8	0.7	0.6	1.6	1.5
Ser 86	2.5	3.5	2.8	2.4	2.4	2.0	1.5	2.3	2.3	2.0
Asp 87	1.5	2.3	2.2	2.0	1.6	1.1	1.3	1.1	2.4	1.7
Thr 89	0.6	0.9	0.5	0.8	0.1	0.1	0.9	0.3	1.4	1.3
Ser 91	1.0	1.0	0.8	0.4	1.2	0.8	1.3	0.8	2.0	1.6
Val 92	0.1	0.0	0.3	0.6	0.4	0.2	0.9	0.4	1.1	1.1
Asn 93	1.2	1.7	1.6	1.8	1.0	0.9	0.4	1.2	0.3	0.4
Cys 94	0.1	0.2	0.1	0.8	0.4	0.3	0.3	0.6	1.2	0.5
Ala 95	1.0	1.0	0.6	0.3	1.0	0.7	1.2	0.7	1.9	1.9
Lys 96	0.9	1.3	1.1	1.2	0.8	0.9	0.2	0.8	0.6	0.4
Lys 97	1.1	1.7	1.4	1.7	1.5	1.2	0.8	1.6	0.9	1.2
Ile 98	1.0	0.7	0.5	0.2	0.5	0.3	1.0	0.4	1.3	1.2
Ser 100	1.1	1.7	1.6	1.8	2.5	2.0	1.5	2.3	2.2	1.9
Gly 102	2.4	3.7	2.7	3.0	3.1	2.5	2.3	2.7	1.6	2.5
Asn 103	1.4	1.9	2.5	2.2	2.5	2.0	1.8	2.5	0.4	0.1
Gly 104	0.7	1.4	1.5	2.1	1.1	0.6	0.1	0.3	0.2	0.6
Met 105	1.3	1.3	0.3	0.1	2.1	1.1	1.8	1.7	2.5	1.9
Asn 106	1.2	1.3	1.4	1.6	1.5	1.0	0.8	1.2	0.4	1.3
Ala 107	0.3	0.8	0.1	0.7	1.1	0.5	0.5	0.2	0.4	0.5
Trp 108	1.1	1.1	0.8	0.5	1.8	0.6	1.8	1.6	1.9	1.5
Val 109	1.8	2.6	1.7	1.6	2.3	1.7	1.8	2.0	2.3	1.6
Trp 111	0.5	1.4	0.2	0.8	0.1	0.5	0.7	0.5	0.9	0.7
Arg 112	0.8	1.6	0.5	0.6	0.5	0.4	1.0	0.9	1.9	1.2

Asn 113	2.5	3.6	2.7	2.4	2.1	1.8	1.8	2.4	2.6	2.2
Cys 115	2.8	3.0	3.1	2.9	2.8	2.3	1.5	2.3	2.4	2.8
Lys 116	0.1	0.3	0.0	0.5	0.8	0.5	0.6	0.2	1.3	0.6
Gly 117	0.6	0.4	0.5	0.7	0.3	0.7	0.2	0.4	0.5	1.0
Val 120	0.1	0.2	0.4	0.4	0.4	0.3	0.5	0.4	1.3	0.6
Gln 121	2.1	2.9	2.3	2.2	1.8	1.5	0.8	1.8	1.3	1.1
Ala 122	0.5	0.8	0.2	0.8	0.7	0.5	0.9	0.4	1.6	1.9
Ile 124	2.2	3.0	2.4	2.2	2.0	1.7	1.2	2.1	2.1	1.8
Arg 125	1.2	2.5	1.2	2.0	0.7	1.0	1.5	1.6	1.1	1.7
Gly 126	1.5	1.5	1.5	1.7	2.7	1.7	1.3	1.2	2.3	2.1
Cys 127	0.8	0.7	0.3	0.2	0.9	0.5	0.3	0.7	0.6	0.7
Arg 128	1.6	1.7	1.4	1.7	2.4	1.5	1.7	2.1	2.2	2.1
Leu 129	0.5	0.4	0.4	0.4	2.0	1.5	1.8	1.9	2.5	2.1
N_{var}	1	6	1	0	4	0	0	0	1	3

Table S20. Hydrogen bonds and secondary-structure assignments for the five X-ray structures *4LZT*, *2VB1*, *1IEE*, *1AKI* and *1HF4*. Hydrogen bonds (HB) were identified according to a geometric criterion: a hydrogen bond was assumed to exist if the hydrogen-acceptor distance was smaller than 0.25 nm and the donor-hydrogen-acceptor angle was larger than 135°. $3_{10:}$-helical backbone hydrogen bonds are indicated as *i-3*, α-helical ones as *i-4*. Secondary-structure assignments (DSSP) were determined using the criteria of the program DSSP[32]. $3_{10:}$-helical backbone assignments are indicated as 3_{10}, α-helical ones as *α*.

Residue donor N-H	Residue acceptor O	*4LZT*		*2VB1*		*1IEE*		*1AKI*		*1HF4*	
		HB	DSSP	HB	DSSP	HB	DSSP	HB	DSSP	HB	DSSP
Leu 8	Gly 4	*i-4*	*α*	*i-4*	*α*	*i-4*	*α*	*i-4*	*α*	*i-4*	*α*
Ala 9	Arg 5	*i-4*	*α*	*i-4*	*α*	*i-4*	*α*	*i-4*	*α*	*i-4*	*α*
Ala 10	Cys 6	*i-4*	*α*	*i-4*	*α*	*i-4*	*α*	*i-4*	*α*	*i-4*	*α*
Ala 11	Glu 7	*i-4*	*α*	*i-4*	*α*	*i-4*	*α*	*i-4*	*α*	*i-4*	*α*
Met 12	Leu 8	*i-4*	*α*	*i-4*	*α*	*i-4*	*α*	*i-4*	*α*	*i-4*	*α*
Lys 13	Ala 9	*i-4*	*α*	*i-4*	*α*	*i-4*	*α*	*i-4*	*α*	*i-4*	*α*
Arg 14	Ala 10	*i-4*	*α*	*i-4*	*α*	*i-4*	*α*	*i-4*	*α*	*i-4*	*α*
His 15	Ala 11	*i-4*		*i-4*		*i-4*				*i-4*	
Gly 16	Lys 13	*i-3*		*i-3*		*i-3*		*i-3*		*i-3*	
Tyr 20	Leu 17	*i-3*		*i-3*		*i-3*		*i-3*		*i-3*	
Gly 22	Asn 19	*i-3*	3_{10}	*i-3*	3_{10}	*i-3*	3_{10}	*i-3*	3_{10}	*i-3*	3_{10}
Tyr 23	Tyr 20	*i-3*		*i-3*		*i-3*		*i-3*		*i-3*	
Asn 27	Ser 24							*i-3*			
Trp 28	Ser 24			*i-4*	*α*						
Val 29	Leu 25	*i-4*	*α*	*i-4*	*α*	*i-4*	*α*	*i-4*	*α*	*i-4*	*α*
Cys 30	Gly 26	*i-4*	*α*	*i-4*	*α*	*i-4*	*α*	*i-4*	*α*	*i-4*	*α*
Ala 31	Asn 27	*i-4*	*α*	*i-4*	*α*	*i-4*	*α*	*i-4*	*α*	*i-4*	*α*
Ala 32	Trp 28	*i-4*	*α*	*i-4*	*α*	*i-4*	*α*	*i-4*	*α*	*i-4*	*α*
Lys 33	Val 29	*i-4*	*α*	*i-4*	*α*	*i-4*	*α*	*i-4*	*α*	*i-4*	*α*
Phe 34	Cys 30	*i-4*	*α*	*i-4*	*α*	*i-4*	*α*	*i-4*	*α*	*i-4*	*α*
Glu 35	Ala 31	*i-4*	*α*	*i-4*	*α*	*i-4*	*α*	*i-4*	*α*	*i-4*	*α*
Ser 36	Ala 32	*i-4*	*α*	*i-4*	*α*	*i-4*	*α*	*i-4*	*α*	*i-4*	*α*
Asn 39	Ser 36	*i-3*		*i-3*		*i-3*				*i-3*	
Ala 42	Asn 39	*i-3*		*i-3*		*i-3*				*i-3*	
Gly 49	Asn 46	*i-3*		*i-3*		*i-3*		*i-3*			
Gln 57	Gly 54	*i-3*		*i-3*		*i-3*		*i-3*		*i-3*	
Trp 63	Asn 59	*i-4*		*i-4*		*i-4*		*i-4*		*i-4*	
Ser 72	Thr 69					*i-3*					
Asn 77	Asn 74	*i-3*		*i-3*		*i-3*		*i-3*		*i-3*	
Ala 82	Pro 79	*i-3*	3_{10}	*i-3*	3_{10}	*i-3*	3_{10}	*i-3*	3_{10}	*i-3*	3_{10}
Leu 83	Cys 80	*i-3*	3_{10}	*i-3*	3_{10}	*i-3*	3_{10}	*i-3*	3_{10}	*i-3*	3_{10}
Leu 84	Ser 81	*i-3*	3_{10}	*i-3*	3_{10}	*i-3*	3_{10}	*i-3*	3_{10}	*i-3*	3_{10}
Ser 85	Ala 82			*i-3*							

Val 92	Ile 88	*i-4*	*α*	*i-4*	*α*	*i-4*	*α*	*i-4*	*α*	*i-4*	*α*
Asn 93	Thr 89	*i-4*	*α*	*i-4*	*α*	*i-4*	*α*	*i-4*	*α*	*i-4*	*α*
Cys 94	Ala 90	*i-4*	*α*	*i-4*	*α*	*i-4*	*α*	*i-4*	*α*	*i-4*	*α*
Ala 95	Ser 91	*i-4*	*α*	*i-4*	*α*	*i-4*	*α*	*i-4*	*α*	*i-4*	*α*
Lys 96	Val 92	*i-4*	*α*	*i-4*	*α*	*i-4*	*α*	*i-4*	*α*	*i-4*	*α*
Lys 97	Asn 93	*i-4*	*α*	*i-4*	*α*						
Ile 98	Cys 94	*i-4*	*α*	*i-4*	*α*	*i-4*	*α*	*i-4*	*α*	*i-4*	*α*
Val 99	Ala 95	*i-4*	*α*	*i-4*	*α*	*i-4*	*α*	*i-4*	*α*	*i-4*	*α*
Ser 100	Lys 96	*i-4*	*α*	*i-4*	*α*						
Ser 100	Lys 97					*i-3*					
Asp 101	Ile 97	*i-4*									
Asp 101	Ile 98					*i-3*		*i-3*	3_{10}	*i-3*	
Gly 102	Val 99	*i-3*		*i-3*							
Asn 106	Asn 103					*i-3*	3_{10}	*i-3*	3_{10}	*i-3*	3_{10}
Ala 107	Gly 104	*i-3*	3_{10}	*i-3*	3_{10}	*i-3*	3_{10}				
Trp 108	Met 105	*i-3*		*i-3*		*i-3*		*i-3*		*i-3*	
Arg 112	Trp 108	*i-4*	*α*	*i-4*	*α*	*i-4*	*α*	*i-4*	*α*	*i-4*	*α*
Asn 113	Val 109	*i-4*	*α*	*i-4*	*α*	*i-4*	*α*	*i-4*	*α*	*i-4*	*α*
Arg 114	Ala 110							*i-4*	*α*	*i-4*	*α*
Cys 115	Trp 111	*i-4*		*i-4*		*i-4*		*i-4*		*i-4*	
Thr 118	Cys 115	*i-3*		*i-3*		*i-3*		*i-3*		*i-3*	
Ala 122	Asp 119	*i-3*	3_{10}	*i-3*	3_{10}	*i-3*	3_{10}	*i-3*	3_{10}	*i-3*	3_{10}
Trp 123	Val 120	*i-3*	3_{10}	*i-3*	3_{10}	*i-3*	3_{10}	*i-3*	3_{10}	*i-3*	3_{10}
Ile 124	Gln 121	*i-3*		*i-3*		*i-3*	3_{10}	*i-3*		*i-3*	3_{10}
Arg 125	Ala 122			*i-3*		*i-3*				*i-3*	3_{10}
Cys 127	Ile 124	*i-3*		*i-3*		*i-3*		*i-3*		*i-3*	

Table S21. $^{13}C^{\alpha}$-$^{13}C'$ RDC values (97) in Hz for HEWL, as obtained from NMR measurements at 308 K and pH = 3.8, Table 4.4 of Ref.[16], and as calculated using three different sets of RDC restraints (*RDC_{CAH59I}*, *RDC_{NH101}*, *$RDC_{NCAH160I}$*) for the X-ray structure ***4LZT*** by applying the alignment-tensor method (*AT*: τ_D^{RDC} = 0, τ_{AT}^{RDC} = 0)[13] or the *HRS* ($K^{RDC,msy}$ = 0) method[14]. $K^{RDC,mfv}$ = 100 kJmol^{-1}Hz^{-2}, $\tau_\theta^{RDC,mfv}$ = 10 ns, in t^{mfv} = 30 ns SD simulations of the magnetic-field vector. The RDC restraint set *RDC_{CAH59I}* is obtained by inverting the sequence of RDC values D_k^0 as given in the fourth column of Table 1. The RDC restraint set *RDC_{NH101}* contains the RDC values D_k^0 given in the second column of Table 2. The RDC restraint set *$RDC_{NCAH160I}$* is obtained by combining the sets of RDC restraints *RDC_{CAH59I}* and *RDC_{NH101}*. The values for the RDCs that are *not* part of the (sub)set of RDC restraints applied, are in bold. *RMSD*: Root-mean-square difference (RMSD) between calculated D_k and D_k^0 RDC values calculated over all, *mfv*-restrained and unrestrained, RDCs. *rRMSD*: RMSD-values calculated over the particular (sub)set of *mfv*-restrained RDCs. *urRMSD*: RMSD-values calculated over the unrestrained RDCs. Deviations of RDC values $D_{k_1k_2}$ (*AT*) or averaged $< D_{k_1k_2} >_{t^{mfv}}$ (*HRS*) from the D_k^0 values larger than 3 Hz are in red. N_{dev}: Number of such deviations. $N_{dev,s}$: Number of RDCs for which the calculated D_k and the D_k^0 values have a different sign. These RDC values are in italics.

Residue	Experimental value (Hz), set *RDC_{CAC97}*	*AT* Restraint set			*HRS* Restraint set		
	D_k^0	*CAH59I*	*NH101*	*NCAH160I*	*CAH59I*	*NH101*	*NCAH160I*
Lys 1	0.97	**0.0**	**1.0**	**0.6**	***-0.2***	**0.9**	**0.6**
Val 2	-2.11	***0.0***	**-1.7**	**-1.1**	***0.0***	**-1.6**	**-1.0**
Phe 3	-0.50	**-0.1**	**-0.6**	**-0.5**	**-0.2**	**-0.7**	**-0.5**
Gly 4	1.95	**0.3**	**1.2**	**0.9**	**0.4**	**1.2**	**0.8**
Glu 7	-1.56	***0.1***	**-1.1**	**-0.5**	***0.6***	**-1.0**	**-0.7**
Leu 8	1.19	**0.2**	**1.0**	**0.6**	**0.1**	**0.9**	**0.6**
Ala 9	1.19	***-0.3***	**1.2**	**0.6**	***-0.8***	**1.3**	**0.7**

Ala 10	-1.30	-0.1	-1.3	-0.9	-0.0	-1.4	-1.0
Ala 11	0.13	0.3	0.3	0.4	0.6	0.4	0.3
Met 12	0.83	-0.0	0.4	0.2	-0.2	0.4	0.2
Lys 13	1.51	-0.3	1.2	0.6	-0.7	1.2	0.7
Arg 14	-1.94	0.0	-2.0	-1.1	0.5	-1.9	-1.3
His 15	1.01	0.4	1.2	0.9	0.5	1.1	0.8
Gly 16	0.55	-0.1	1.0	0.6	-0.4	1.1	0.7
Asp 18	1.71	0.2	1.2	0.8	0.4	1.2	0.8
Asn 19	-1.81	-0.1	-1.6	-1.0	0.2	-1.6	-1.1
Tyr 23	-0.67	0.2	-0.8	-0.3	0.6	-0.7	-0.4
Ser24	1.34	0.3	1.0	0.7	0.3	0.9	0.7
Leu 25	0.76	-0.4	1.5	0.8	-0.7	1.6	0.8
Asn 27	-0.39	0.2	-0.3	-0.2	0.1	-0.5	-0.2
Trp 28	1.41	0.1	1.2	0.8	0.3	1.2	0.7
Cys 30	-2.86	0.1	-2.5	-1.6	0.3	-2.5	-1.6
Ala 31	1.21	0.3	0.9	0.6	0.3	0.8	0.6
Ala 32	1.67	-0.3	1.3	0.7	-0.4	1.3	0.7
Lys 33	-0.74	-0.1	-0.6	-0.4	-0.2	-0.5	-0.3
Phe 34	-2.53	0.0	-2.3	-1.5	0.2	-2.4	-1.5
Ser 36	-1.04	0.1	-0.9	-0.4	0.6	-0.8	-0.5
Asn 37	0.81	-0.1	0.8	0.5	-0.3	0.9	0.6
Phe 38	0.83	0.2	0.2	0.1	0.1	0.0	0.1
Asn 39	-0.43	-0.2	-0.4	-0.3	-0.1	-0.4	-0.4
Thr 40	-2.42	0.1	-2.2	-1.4	0.2	-2.2	-1.4
Gln 41	0.14	-0.1	0.4	0.3	-0.2	0.5	0.4
Ala 42	-2.63	0.1	-1.9	-1.1	0.5	-1.8	-1.2
Asn 44	-1.67	0.0	-2.3	-1.3	0.4	-2.2	-1.4
Asn 46	-2.51	0.0	-2.4	-1.4	0.2	-2.3	-1.5
Thr 47	-0.95	-0.0	-0.1	-0.0	0.3	-0.1	-0.2
Asp 48	1.48	-0.2	1.0	0.5	-0.6	0.9	0.6
Gly 49	-0.60	-0.1	-0.7	-0.4	-0.3	-0.5	-0.3
Ser 50	-1.22	0.1	-0.8	-0.4	0.5	-0.7	-0.5
Thr 51	-1.57	0.0	-1.1	-0.7	-0.0	-1.1	-0.7
Tyr 53	-1.29	0.0	-2.3	-1.4	0.2	-2.2	-1.4
Leu 56	-2.63	0.0	-1.8	-1.1	0.2	-1.7	-1.1
Gln 57	-0.66	0.1	-0.2	0.0	0.5	-0.1	-0.1
Asn 59	-1.71	-0.2	-0.5	-0.4	-0.3	-0.6	-0.5
Ser 60	-2.19	0.2	-1.0	-0.4	0.7	-0.9	-0.5
Arg 61	1.21	-0.2	1.3	0.7	-0.4	1.4	0.8
Cys 64	-2.38	0.1	-2.0	-1.3	0.1	-2.0	-1.2
Asn 65	0.92	0.2	1.3	0.9	0.3	1.3	0.9
Asp 66	-2.07	0.1	-2.4	-1.5	0.2	-2.4	-1.5
Gly 67	0.08	-0.3	0.4	0.2	-0.6	0.6	0.3
Arg 68	1.42	0.2	1.1	0.8	0.2	1.0	0.8
Gly 71	-2.77	-0.1	-1.1	-0.6	-0.1	-0.9	-0.6
Asn 74	0.44	-0.0	0.2	0.1	-0.2	0.2	0.1
Leu 75	-2.30	0.0	-1.3	-0.7	0.3	-1.1	-0.7
Cys 76	1.80	-0.0	0.8	0.4	-0.3	0.6	0.5
Asn 77	0.62	0.3	0.7	0.6	0.6	0.7	0.5
Pro 79	0.26	-0.2	-0.4	-0.4	-0.2	-0.5	-0.5

Cys 80	-0.23	*0.2*	-0.1	*0.1*	*0.7*	-0.0	*0.0*
Ser 81	-1.71	-0.1	-1.5	-0.9	-0.1	-1.4	-0.9
Ala 82	-0.80	-0.1	-0.7	-0.6	-0.1	-0.9	-0.6
Leu 83	1.12	*-0.1*	0.8	0.5	0.1	0.9	0.5
Leu 84	-0.72	*0.1*	-0.2	*0.0*	*0.2*	-0.0	*0.1*
Ser 85	-2.60	-0.0	-1.8	-1.2	*0.2*	-1.9	-1.3
Ile 88	2.38	*-0.1*	0.9	0.6	0.0	1.0	0.5
Thr 89	-1.36	*0.1*	-1.7	-0.9	*0.5*	-1.6	-1.0
Ala 90	1.77	*-0.0*	1.2	0.7	*-0.3*	1.1	0.8
Ser 91	1.06	0.2	0.3	0.2	0.1	0.2	0.2
Val 92	1.08	*-0.1*	0.3	0.2	0.2	0.3	0.1
Asn 93	-1.11	*0.1*	-0.8	-0.4	*0.3*	-0.7	-0.4
Cys 94	0.75	0.1	0.6	0.3	*-0.2*	0.4	0.4
Ala 95	1.20	0.1	1.2	0.7	0.0	1.1	0.7
Lys 96	-1.79	*0.1*	-1.5	-0.8	*0.6*	-1.3	-0.9
Lys 97	0.79	*-0.1*	1.0	0.6	*-0.4*	1.1	0.7
Ile 98	-1.68	*0.3*	*0.4*	*0.3*	*0.2*	*0.2*	*0.3*
Val 99	1.81	*-0.2*	1.2	0.7	*-0.2*	1.2	0.7
Ser 100	-2.11	*0.1*	-2.0	-1.1	*0.5*	-1.8	-1.1
Gly 102	0.52	*-0.4*	1.3	0.7	*-0.7*	1.3	0.7
Gly 104	1.94	0.1	1.3	0.9	0.2	1.3	0.8
Met 105	0.41	*-0.3*	0.6	0.3	*-0.6*	0.7	0.4
Asn 106	-0.99	*0.2*	-1.2	-0.8	*0.1*	-1.4	-0.8
Ala 107	1.44	0.1	1.2	0.8	0.2	1.3	0.8
Ala 110	-1.26	*0.1*	-0.9	-0.6	*0.0*	-1.0	-0.6
Trp 111	1.12	0.2	1.1	0.7	0.1	1.0	0.8
Asn 113	-2.61	*0.1*	-1.9	-1.1	*0.6*	-1.8	-1.2
Arg 114	0.28	*-0.1*	*-0.9*	*-0.6*	*-0.2*	*-0.8*	*-0.5*
Cys 115	1.25	0.3	1.1	0.8	0.4	1.0	0.8
Lys 116	-0.35	-0.1	-0.2	-0.2	-0.3	-0.3	-0.2
Thr 118	-0.81	-0.2	-0.5	-0.3	-0.3	-0.4	-0.2
Val 120	-0.92	-0.0	-0.6	-0.3	*0.0*	-0.4	-0.2
Gln 121	1.11	0.1	0.8	0.6	0.4	0.8	0.5
Ala 122	0.73	0.2	0.5	0.3	0.1	0.3	0.3
Trp 123	0.43	*-0.2*	0.7	0.4	*-0.4*	0.8	0.5
Ile 124	-3.36	*0.0*	-2.5	-1.5	*0.4*	-2.5	-1.6
Arg 125	0.76	0.3	0.7	0.6	0.6	0.7	0.6
Gly 126	0.95	*-0.1*	0.7	0.3	*-0.4*	0.6	0.4
Cys 127	0.43	*-0.3*	0.9	0.5	*-0.7*	1.0	0.5
Arg 128	-0.62	-0.1	-0.9	-0.6	*0.2*	-0.9	-0.7
RMSD		1.5	0.6	0.9	1.7	0.6	0.8
rRMSD		-	-	-	-	-	-
urRMSD		1.5	0.6	0.9	1.7	0.6	0.8
N_{dev}		1	0	0	4	0	0
$N_{dev,s}$		*58*	*3*	*6*	*60*	*3*	*5*

Table S22. $^{13}C'$-^{15}N RDC values (45) in Hz for HEWL, as obtained from NMR measurements at 308 K and pH = 3.8, Table 4.5 of Ref.[16], and as calculated using three different sets of RDC restraints (RDC_{CAH59I}, RDC_{NH101}, $RDC_{NCAH160I}$) for the X-ray structure ***4LZT*** by applying the alignment-tensor method (*AT*: τ_D^{RDC} = 0, τ_{AT}^{RDC} = 0)[13] or the *HRS* ($K^{RDC,msy}$ = 0) method[14]. $K^{RDC,mfv}$ = 100 kJmol^{-1}Hz^{-2}, $\tau_\theta^{RDC,mfv}$ = 10 ns, in t^{mfv} = 30 ns SD simulations of the magnetic-field vector. The RDC restraint set RDC_{CAH59I} is obtained by inverting the sequence of RDC values D_k^0 as given in the fourth column of Table 1. The RDC restraint set RDC_{NH101} contains the RDC values D_k^0 given in the second column of Table 2. The RDC restraint set $RDC_{NCAH160I}$ is obtained by combining the sets of RDC restraints RDC_{CAH59I} and RDC_{NH101}. The values for the RDCs that are *not* part of the (sub)set of RDC restraints applied, are in bold. *RMSD*: Root-mean-square difference (RMSD) between calculated D_k and D_k^0 RDC values calculated over all, *mfv*-restrained and unrestrained, RDCs. *rRMSD*: RMSD-values calculated over the particular (sub)set of *mfv*-restrained RDCs. *urRMSD*: RMSD-values calculated over the unrestrained RDCs. Deviations of RDC values $D_{k_1k_2}$ (*AT*) or averaged $< D_{k_1k_2} >_{t^{mfv}}$ (*HRS*) from the D_k^0 values larger than 3 Hz are in red. N_{dev}: Number of such deviations. $N_{dev,s}$: Number of RDCs for which the calculated D_k and the D_k^0 values have a different sign. These RDC values are in italics.

Residue	Experimental value (Hz), set RDC_{CN45}	*AT* Restraint set			*HRS* Restraint set		
	D_k^0	*CAH59I*	*NH101*	*NCAH160I*	*CAH59I*	*NH101*	*NCAH160I*
Gly 4	-0.55	**-0.0**	**-0.2**	**-0.1**	***0.1***	**-0.1**	**-0.1**
Glu 7	0.20	**0.1**	***-0.0***	***-0.0***	**0.1**	***-0.1***	**-0.1**
Leu 8	0.67	***-0.0***	**0.7**	**0.5**	***-0.0***	**0.8**	**0.5**
Ala 9	-0.38	**-0.2**	**-0.1**	**-0.2**	**-0.3**	**-0.1**	**-0.2**
Ala 10	0.42	***-0.0***	**0.6**	**0.4**	**0.0**	**0.6**	**0.4**
Ala 11	-0.95	***0.0***	**-0.7**	**-0.4**	***0.2***	**-0.7**	**-0.5**
Met 12	2.41	***-0.0***	**1.7**	**1.0**	***-0.2***	**1.6**	**1.1**

Lys 13	-0.78	**-0.2**	**-0.7**	**-0.5**	**-0.2**	**-0.6**	**-0.5**
Arg 14	1.07	**0.1**	**0.4**	**0.3**	**0.1**	**0.3**	**0.2**
Gly 16	-0.95	***0.2***	**-0.9**	**-0.5**	***0.4***	**-1.0**	**-0.5**
Trp 28	1.37	***-0.0***	**1.3**	**0.8**	***-0.3***	**1.3**	**0.9**
Ala 31	-0.23	**-0.0**	**-0.2**	**-0.2**	***0.0***	**-0.3**	**-0.2**
Ala 32	0.38	**0.0**	**0.9**	**0.6**	***-0.0***	**1.0**	**0.6**
Lys 33	-0.49	**-0.2**	**-0.4**	**-0.4**	**-0.4**	**-0.4**	**-0.4**
Asn 37	-1.12	***0.2***	**-0.9**	**-0.5**	***0.3***	**-0.9**	**-0.5**
Phe 38	1.72	***-0.0***	**1.2**	**0.7**	***-0.0***	**1.1**	**0.7**
Asn 39	-0.78	***0.1***	**-0.7**	**-0.4**	***0.3***	**-0.7**	**-0.5**
Thr 40	-1.41	***0.1***	**-0.8**	**-0.5**	***0.2***	**-0.8**	**-0.5**
Gln 41	-0.26	**-0.1**	**-0.3**	**-0.1**	**-0.0**	**-0.1**	**-0.2**
Thr 47	-1.13	***0.0***	**-0.5**	**-0.3**	***0.2***	**-0.5**	**-0.3**
Arg 61	1.21	***-0.1***	**1.2**	**0.8**	***-0.1***	**1.3**	**0.8**
Arg 68	-0.64	**-0.1**	**-0.3**	**-0.1**	**-0.0**	**-0.2**	**-0.2**
Leu 75	-0.57	**-0.1**	**-0.4**	**-0.3**	**-0.3**	**-0.4**	**-0.3**
Cys 76	1.35	***-0.0***	**1.4**	**0.9**	***-0.2***	**1.5**	**0.9**
Asn 77	-0.81	***0.2***	**-0.9**	**-0.5**	***0.3***	**-0.9**	**-0.5**
Ser 81	-1.23	***0.1***	**-0.9**	**-0.5**	***0.2***	**-0.9**	**-0.5**
Leu 83	-0.89	**-0.2**	**-0.5**	**-0.3**	**-0.2**	**-0.4**	**-0.3**
Leu 84	-0.63	***0.2***	**-0.6**	**-0.3**	***0.4***	**-0.6**	**-0.3**
Ala 90	-0.78	***0.2***	**-0.3**	**-0.1**	***0.2***	**-0.3**	**-0.1**
Asn 93	-0.28	***0.1***	**-0.3**	**-0.2**	***0.2***	**-0.4**	**-0.2**
Ala 95	-0.40	***0.2***	**-0.6**	**-0.3**	***0.4***	**-0.6**	**-0.4**
Lys 96	1.14	***-0.1***	**0.9**	**0.6**	***-0.1***	**1.0**	**0.6**
Lys 97	-1.40	***0.2***	**-0.9**	**-0.5**	***0.3***	**-0.9**	**-0.5**
Gly 104	0.60	**0.0**	**1.1**	**0.7**	***-0.2***	**1.1**	**0.7**
Met 105	-0.05	**-0.2**	**-0.3**	**-0.3**	**-0.3**	**-0.3**	**-0.3**
Ala 110	-1.01	***0.2***	**-0.9**	**-0.5**	***0.3***	**-0.9**	**-0.5**
Trp 111	1.13	***-0.1***	**1.1**	**0.6**	***-0.3***	**1.0**	**0.6**
Asn 113	-0.28	***0.0***	**-0.3**	**-0.2**	***0.1***	**-0.4**	**-0.3**
Arg 114	-1.14	***0.2***	**-0.8**	**-0.4**	***0.2***	**-0.8**	**-0.4**
Cys 115	0.97	***-0.1***	**0.6**	**0.3**	***-0.4***	**0.6**	**0.3**
Gln 121	-0.78	***0.2***	**-0.8**	**-0.4**	***0.5***	**-0.9**	**-0.5**
Ala 122	1.68	**0.0**	**1.0**	**0.6**	***-0.1***	**1.0**	**0.7**
Arg 125	1.26	**0.0**	**0.8**	**0.5**	***-0.0***	**0.8**	**0.5**
Gly 126	0.52	**0.0**	**0.8**	**0.5**	***-0.1***	**0.7**	**0.5**
Cys 127	-0.67	**-0.1**	**-0.7**	**-0.4**	**-0.0**	**-0.6**	**-0.5**
RMSD		**1.0**	**0.3**	**0.6**	**1.1**	**0.4**	**0.5**
rRMSD		-	-	-	-	-	-
urRMSD		**1.0**	**0.3**	**0.6**	**1.1**	**0.4**	**0.5**
N_{dev}		**0**	**0**	**0**	**0**	**0**	**0**
$N_{dev,s}$		***27***	***1***	***1***	***33***	***1***	***0***
